# Fungi vs. Fungi in Biocontrol: An Overview of Fungal Antagonists Applied Against Fungal Plant Pathogens

**DOI:** 10.3389/fcimb.2020.604923

**Published:** 2020-11-30

**Authors:** Kasun M. Thambugala, Dinushani A. Daranagama, Alan J. L. Phillips, Sagarika D. Kannangara, Itthayakorn Promputtha

**Affiliations:** ^1^ Department of Plant and Molecular Biology, Faculty of Science, University of Kelaniya, Kelaniya, Sri Lanka; ^2^ Genetics and Molecular Biology Unit, Faculty of Applied Sciences, University of Sri Jayewardenepura, Nugegoda, Sri Lanka; ^3^ Faculdade de Ciências, Biosystems and Integrative Sciences Institute (BioISI), Universidade de Lisboa, Lisbon, Portugal; ^4^ Department of Biology, Faculty of Science, Chiang Mai University, Chiang Mai, Thailand; ^5^ Research Center in Bioresources for Agriculture, Industry and Medicine, Chiang Mai University, Chiang Mai, Thailand

**Keywords:** biocontrol agents, disease control, fungicides, plant diseases, plant pathogens, phylogeny, *Trichoderma*

## Abstract

Plant pathogens cause severe losses or damage to crops worldwide and thereby significantly reduce the quality and quantity of agricultural commodities. World tendencies are shifting towards reducing the usage of chemically synthesized pesticides, while various biocontrol methods, strategies and approaches are being used in plant disease management. Fungal antagonists play a significant role in controlling plant pathogens and diseases and they are used as Biocontrol Agents (BCAs) throughout the world. This review provides a comprehensive list of fungal BCAs used against fungal plant pathogens according to modern taxonomic concepts, and clarifies their phylogenetic relationships because thewrong names are frequently used in the literature of biocontrol. Details of approximately 300 fungal antagonists belonging to 13 classes and 113 genera are listed together with the target pathogens and corresponding plant diseases. *Trichoderma* is identified as the genus with greatest potential comprising 25 biocontrol agents that have been used against a number of plant fungal diseases. In addition to *Trichoderma*, nine genera are recognized as significant comprising five or more known antagonistic species, namely, *Alternaria*, *Aspergillus*, *Candida*, *Fusarium*, *Penicillium*, *Pichia*, *Pythium*, *Talaromyces*, and *Verticillium*. A phylogenetic analysis based on partial sequences of the 28S nrRNA gene (LSU) of fungal antagonists was performed to establish their phylogenetic relationships.

## Introduction

Plant pathogens including fungi, bacteria, viruses and nematodes cause serious losses or damage to crops worldwide and significantly reduce the quality and quantity of agricultural commodities. These losses pose a major threat to global food production annually (El Ghaouth et al., 2002; Dean et al., 2012; Singh, 2014; O’Brien, 2017). Moreover, pathogenic infection in the field or in post-harvest storage can affect the health of humans and livestock, especially if the pathogen produces toxins in or on consumable products (Brimner and Boland, 2003; Menzler-Hokkanen, 2006).

Various methods, strategies, and approaches are used in the management of plant diseases. These encompass the development of resistant varieties through plant breeding, genetically engineered plants, use of agrochemicals and physical methods (i.e., heat treatments, UV irradiation, modified or controlled atmosphere, cold storage, and inducing resistance by applying elicitors), application of biological control agents and good agronomic and horticultural practices (Stevens et al., 1997; Wisniewski et al., 2000; Droby, 2006; Singh and Chawla, 2012; Gupta and Sharma, 2014; Singh, 2014; O’Brien, 2017). These approaches have contributed significantly to the remarkable improvements in crop productivity and quality over the past few decades (Punja, 1997; Droby, 2006; Chandrashekara et al., 2012).

### Biological Control: Overview and Significance

Biological control approaches of plant diseases include any reduction in the amount or the effect of pathogens (disease-producing activity) that is achieved through the induction of biological mechanisms or the action of naturally occurring or introduced antagonists, that occurs by manipulating the microenvironment to favour the activity of antagonists (Baker, 1987; Stirling and Stirling, 1997). Microbial biocontrol agents (BCAs) for plant diseases are usually fungal or bacterial strains isolated from the phyllosphere, endosphere or rhizosphere and they play an important role in controlling plant-pathogenic organisms. Biocontrol agents or microbial antagonists prevent infection of the host plant by the pathogen, or establishment of the pathogen in the host plant. The principal mechanisms for the control have been assumed to be those that act primarily upon the pathogens. The antagonists can exhibit several direct or indirect mechanisms of action involved in biological disease control. These mechanisms include; antibiosis (where an inhibitory metabolite or antibiotic is produced by the antagonist), mycoparasitism (where the antagonist derives some or all of its nutrients from the fungal host), induced resistance (induction of plant defense response against plant pathogens) and growth enhancement (BCAs promote plant growth while the effects of the disease are being reduced and also through microbial hormones such as indoleacetic acidand gibberellic acid). Secretion of extracellular hydrolytic enzymes by the antagonist, competition for space and nutrients between organisms and detoxification of virulence factors are other actions involved in biological disease control (Wilson et al., 1991; Punja, 1997; Heydari and Pessarakli, 2010; Chandrashekara et al., 2012; Singh, 2014; Zhang et al., 2014; Deketelaere et al., 2017). Recent studies have demonstrated that effects such as induced systemic or localized resistance by microbial BCAs on plants are also crucial. These fungi or bacteria can colonize the root epidermis and outer cortical layers and release bioactive molecules that cause walling-off of the fungal thallus or bacterial colonies (Harman, 2006). Consequently, they will alter the transcriptome and the proteome machinery of plants substantially. This alteration in the plant’s genetic material will provide certain additional advantages to the plant, such as increased plant growth and nutrient uptake in addition to induction of pathways for resistance in plants.

World trends are shifting towards reducing the use of agrochemicals in the management of plant diseases. Considerable research effort today is focused on seeking safe, eco-friendly and effective alternatives to synthetic, chemical fungicides to reduce the decay loss in harvested commodities and to control crop diseases in the field that lead to significant economic losses (Stirling and Stirling, 1997; Wisniewski et al., 2000; Droby, 2006; Chandrashekara et al., 2012). Due to the aforementioned mechanisms, biological control agents for plant diseases are gaining stature as viable alternatives to synthetic pesticides given their perceived increased level of safety and minimal negative environmental impacts. It is imperative to continue this line of research, since regulations on the use of new and existing fungicides are becoming more and more stringent. In particular, this has led to extensive researches on the use of microbial antagonists as protective agents and many fungal diseases can now be controlled by microbial antagonists. As a result, commercial products containing microbial BCAs have been successfully exploited in modern agriculture (e.g., *Trichoderma* based products and biopesticides based on *Bacillus thuringiensis*) (Menzler-Hokkanen, 2006).

A significant amount of harvested fruits and vegetables is lost annually due to microbial spoilage and this loss can range from 10%–50% depending on the commodity and country (El Ghaouth et al., 2002; Janisiewicz and Korsten, 2002). Developing countries experience greater losses due to inadequate storage and transportation facilities, and improper handling methods that are employed during harvesting and transit (Pathak, 1997; El Ghaouth et al., 2002; Nabi et al., 2017). The harvested yield might have been infected by one or several pathogens prior to harvest or they may become infected during transit and storage. (Punja, 1997; Janisiewicz and Korsten, 2002; Nabi et al., 2017). Several researches have been carried out to identify effective biocontrol agents for post-harvest disease management and as a result, biocontrol antagonists are now employed to control postharvest diseases worldwide. A few examples of these applications are indicated here. Mohamed and Saad (2009) found that the application of specific strains of *Pichia anomala* was a safe and effective biocontrol agent against Diplodia postharvest rot of guava fruit caused by *Lasiodiplodia theobromae* (Pat.) Griffon & Maubl. Alvindia and Natsuaki (2008) and Sangeetha et al. (2009) demonstrated the superior biocontrol potential of *Trichoderma* species for the management of the postharvest crown rot complex of banana caused by a variety of fungal pathogens including *Colletotrichum musae*, *Fusarium verticillioides*, and *Lasiodiplodia theobromae*. The search for suitable biological-control systems has largely taken place in the last fifty years and there has been considerable interest in the use of antagonistic microorganisms for the control of postharvest diseases. (Droby, 2006; Heydari and Pessarakli, 2010; Wisniewski et al., 2016). Bacteria associated with plants are known to develop biofilms on plant surfaces and within intercellular spaces of plant tissues, which act as microniches. It has been reported that the conditions within these microniches created because of the biofilm formation are markedly different from those of the ambient environment, which will eventually lead the microbial cells to effect functions that are not possible alone. This may influence the ecology of the bacteria they harbor and the relationship of bacteria with plants, which directly influence the development of strategies for biological control of plant disease and for assuring food safety (Morris and Monier, 2003).

### Fungal Antagonists

**Figure 1:**
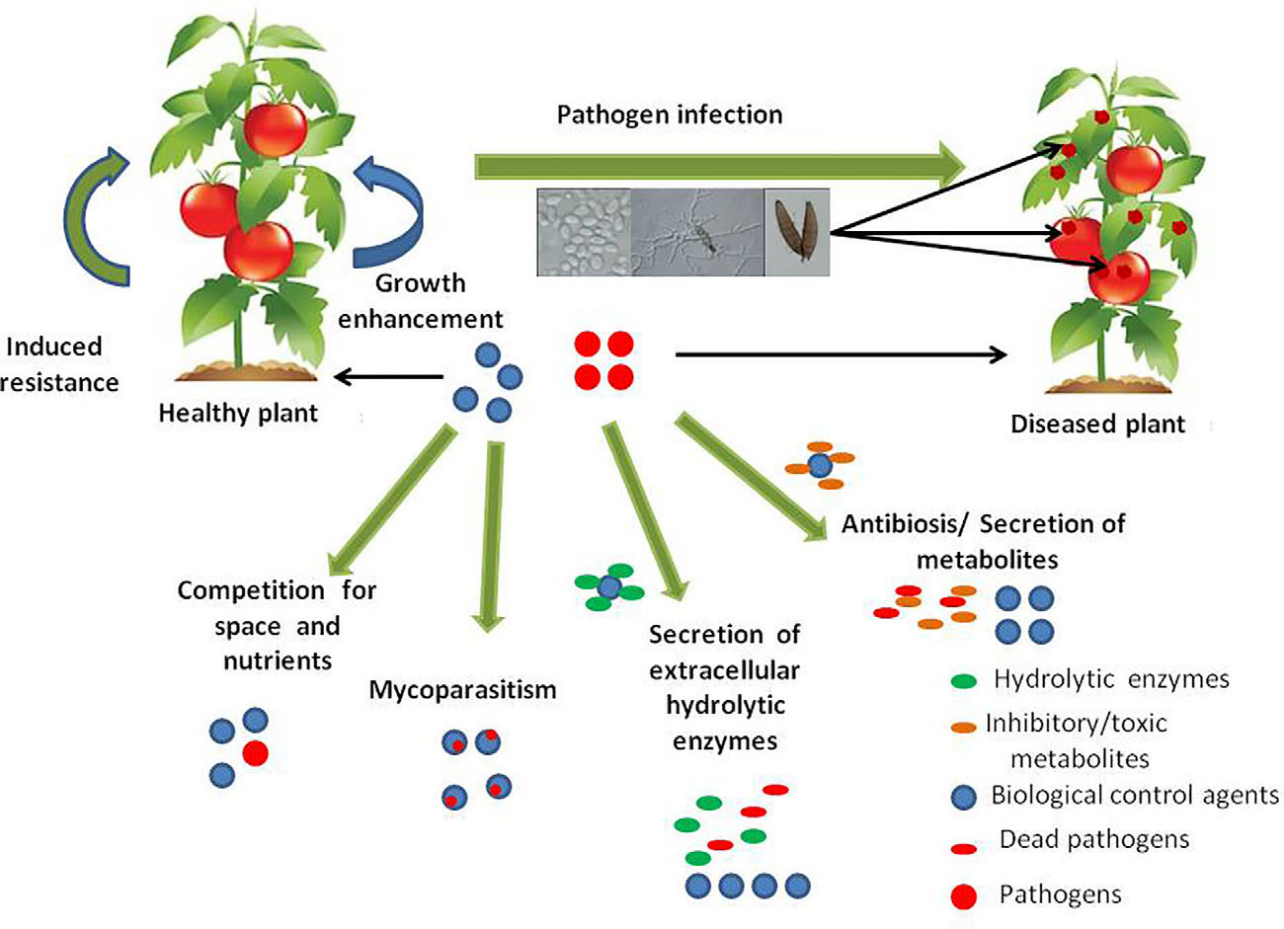
Key mechanisms of action involved in biological control of plant fungal diseases by fungal antagonists.

The potential for the application of fungal biological control agents against plant pathogens has largely increased because fungi have a comparatively high reproductive rate (sexually as well as asexually), a short generation time and they are target specific. Furthermore, in the absence of the host, they can survive in the environment shifting their mode of parasitism to saprotrophism thus maintaining sustainability. Many fungal species possess mechanisms that allow them to efficiently protect plants from diseases caused by plant pathogenic fungi (**Figure 1**).

### History of Fungal Biological Control Applications

Since ancient times man has attempted to increase crop production and control disease severity of crop plants by altering cultivation practices, which reduce both initial inoculum as well as infection rate (Singh and Chawla, 2012; Gupta and Sharma, 2014). With the finding of microorganisms and their interactions, many methods have been employed to control pathogens through the use of fungal antagonists.

Roberts (1874) showed the antagonistic action of microorganisms in liquid cultures between *Penicillium glaucum* and bacteria, introducing the term antagonism as used in microbiology. Hartley (1921) made the first attempt at direct application of biological control of plant pathogens by inoculating soil with microorganisms that were thought to have antagonistic potential. He inoculated forest nursery soils with thirteen antagonistic fungi to control damping-off caused by *Pythium debaryanum*. (Baker, 1987; Gupta and Sharma, 2014). Weindling (1932, 1934) described the potential of *Trichoderma lignorum* (*T. viride*) to control plant-pathogenic fungi by mycoparasitism and reported the first use of a known antimycotic-producing antagonist in plant disease control (Baker, 1987). Later, Weindling (1941) noted that *Trichoderma* species excrete an antimycotic that was toxic to plant pathogens including *Rhizoctonia solani* and *Sclerotinia americana*, and named it gliotoxin. This was the first record of the use of a known antimycotic-producing antagonist in plant disease control (Baker, 1987; Howell, 2003). The discovery of penicillin by A. Fleming in 1928, and its purification and use in pharmaceutical production, significantly stimulated studies on antagonists of plant pathogens (Baker, 1987).

Development of modern biotechnological approaches lead to increase the potential usage of fungal antagonists against a wide range of plant diseases. Numerous researches and experiments have been carried out during the past few decades to identify new fungal BCAs and evaluate their effectiveness under different environmental conditions.

### Commercialization of Fungal Biological Control Agents

Commercial uses and applications of biological control of plant diseases have been slow mainly due to their variable performances under different environmental conditions in the field as well as due to their host specificity. To overcome this problem, it is essential to develop new formulations of BCAs with a higher degree of stability, efficiency and survival using new biotechnological practices (Heydari and Pessarakli, 2010). Several criteria have to be satisfied for upscaling a particular BCA to reach the stage of commercialization (Punja, 1997). Commercialization of biological control agents is expensive as it involves many steps such as isolation in pure culture or enrichment of the microorganism, identification and characterization, the development of a suitable formulation, mass production, efficacy testing of the product, inspection of storage stability, finding an industrial partner, attention to human and environmental safety matters, registration and marketing (Punja, 1997; Stirling and Stirling, 1997; Janisiewicz and Korsten, 2002; Montesinos, 2003). A number of biologically based products are being sold worldwide for the control of fungal plant pathogens and generally they are produced as granules, wettable powders, dusts, and aqueous or oil-based liquid products using different mineral and organic carriers (Ardakani et al., 2009; Nega, 2014). Several microbial antagonists have been patented and evaluated for commercial uses (Wisniewski et al., 2000; El Ghaouth et al., 2002; Schena et al., 2004; Nabi et al., 2017) and these agents are frequently recommended for plants (Albajes et al., 2000; Fravel, 2005; O’Brien, 2017). Some commercialized fungal BCAs used to control plant fungal diseases and their particulars are listed in **Table 1**.

**Table 1:** Some commercialized fungal Biocontrol Agents (BCAs) for plant fungal diseases and their specifications.

Biocontrol agent	Product	Target Pathogen(s) or crop disease	Manufacturer or distributor
*Ampelomyces quisqualis*	AQ10^®^ Bio Fungicide	Powdery mildew	Ecogen Inc, USA, Israel
*Anthracocystis flocculosa* (*Pseudozyma flocculosa*)	Sporodex L	Powdery mildew	Plant Products Co., Canada
*Candida oleophila*	Aspire	Post-harvest diseases	Ecogen Inc, USA, Israel
*Paraphaeosphaeria minitans* (*Coniothyrium minitans*)	Contans WG; KONI	*Sclerotinia sclerotiorum* and *S. minor*	Prophyta Biologischer Pflanzenschutz GmbH; Germany, Bioved Ltd, Hungary
*Clonostachys rosea* (*Gliocladium catenulatum*)	Primastop	Damping-off, seed rot, root and stem rot, and wilt diseases	Kemira Agro OY, Finland; RegWest Co., USA
	Prestop	Soil-Borne and foliar diseases of greenhouse vegetables, herbs and ornamentals	Danstar Ferment Ag., Switzerland; AgBio, Inc., USA
*Fusarium oxysporum* (non-pathogenic)	Fusaclean; Biofox C	Wilt diseases	SIAPA, Italy; Natural Plant Protection, France
*Phlebiopsis gigantea*	Rotstop^®^	Root rot diseases	Kemira Agro Oy, Finland
*Trichoderma virens* (*Gliocladium virens*)	Soilgard^®^	Soil-borne pathogens; *Rhizoctonia* and *Pythium* species	Certis USA
*Trichoderma harzianum*	RootShield^®^	Root rot diseases; *Pythium*, *Fusarium*, *Rhizoctonia*, *Thielaviopsis* and *Cylindrocladium* species	BioWorks, Inc., USA
*Trichoderma harzianum*	Trichodex	Grey mould (*Botrytis cinerea*); *Rhizoctonia*, *Sclerotinia* and *Colletotrichum* species	Makhteshim Agan Industries, Israel
*Trichoderma harzianum* and *T. polysporum*	Binab T	Root rot diseases, pruning wounds in ornamental, shade, and forest trees	BINAB Bio-Innovation AB, Sweden
*Trichoderma viride*	*Trichoderma Viride* Trieco	Soil-borne fungal diseases	Ecosense Lab (I) Pvt. Ltd., India

### Integrated Applications of BCAs With Synthetic Fungicides for the Control of Plant Fungal Pathogens

Synthetic fungicides consisting of inorganic or organic compounds are commonly used in developed agricultural systems to control plant diseases, including post-harvest diseases, and to safeguard crop yield and quality mainly due to their relatively low cost, ease of application, and effectiveness. Chemical agents such as Captan, dithiocarbamates, thiabendazole (TBZ) and imazalil (IMZ) are widely used in the control of plant fungal pathogens (Lucas et al., 2015; Perez et al., 2016; Gupta, 2018). However, the massive and indiscriminate use of synthetic fungicides in crop protection and post-harvest food preservation has resulted in resistance to some fungicides and also led to severe effects on humans, animals, and wildlife resulting in widespread adverse ecological effects (Gupta, 2018; Gupta, 2019). Significant biocontrol of postharvest diseases of fruits and vegetables can be achieved with both field and postharvest applications (Janisiewicz and Korsten, 2002). The combined or integrated applications of a BCA with a synthetic fungicide or physical additives, either simultaneously or in rotation, would be expected to result in an enhanced degree of disease suppression, provided that the biocontrol agent is compatible with the fungicide used (Punja, 1997; Janisiewicz and Korsten, 2002; Droby, 2006; Eshel et al., 2009).

### Modern Biotechnological Approaches Used in Plant Fungal Pathogen Biocontrol

Biological control of plant diseases using fungal BCAs has developed considerably in recent years with the application of genomics, genetic engineering and recombinant DNA techniques. These techniques have been developed to a high degree of precision and have been applied to the improvement of fungal strains for agro-industrial processes. Development of new crop varieties or clones that are resistant to plant pathogens offers a commonly acceptable and potentially long-term control option. Furthermore, many studies have been conducted to identify genetic traits of fungal antagonists and determine their potential to enhance biocontrol activity (Janisiewicz and Korsten, 2002; Droby, 2006; O’Brien, 2017). A few examples are pointed out here; (a). the introduction of multiple lytic enzyme‐encoding genes into *Trichoderma virens* genome resulted in a strain that secreted a mixture of glucanases and showed greatly enhanced inhibition of the pathogens *Pythium ultimum* (Oomycota, Chromista), *Rhizoctonia solani*, and *Rhizopus oryzae* (Djonovic et al., 2007); (b). McDougal et al. (2012) presented a method for the genetic transformation of *Cyclaneusma minus*, the causal agent of Cyclaneusma needle-cast, using protoplasts generated by incubation with Glucanex™ enzyme. *Cyclaneusma minus* was transformed with a gene encoding green fluorescent protein (GFP), which was allowed to identify several *Trichoderma* strains with potential for biocontrol of the disease. The interaction between *C. minus* and the *Trichoderma* strains, in the interaction zone where GFP expression was lost, was determined by a dual culture technique to be fungicidal; (c). Yakoby et al. (2001) generated reduced-pathogenicity mutants of the avocado fruit pathogen *Colletotrichum gloeosporioides* using insertional mutagenesis by restriction enzyme mediated integration (REMI) transformation and these isolates can be used for the biological control of anthracnose caused by *C. gloeosporioides*.

### Aims of This Study

The present study aims to provide a comprehensive list of fungal BCAs that are used against fungal pathogens of crop plants, and clarify their phylogenetic relationships as these are often wrongly mentioned and interpreted in the literature of biocontrol. In this review, the main researches conducted during past the fifty years to evaluate the interactions between fungal antagonists and fungal plant pathogens were highlighted. Thus, this review is meant to serve as an updated database for applications of potential fungal antagonists against particular plant fungal diseases employed by different researchers worldwide. This can also serve as a useful tool to select and compare suitable and most applicable fungal antagonistic applications for fungal disease management in ongoing practices and researches. Many fungal antagonists and causative agents of plant diseases are presently identified and described based on the traditional classification and taxonomic systems. This review is the first comprehensive study of potential fungal antagonists applied against fungal plant pathogens based on a phylogenetic analysis and provides an extensive list of fungal BCAs used against fungal plant pathogens according to modern taxonomic concepts.Therefore, in this review the fungal antagonists and fungal pathogens are presented and listed using updated taxonomic nomenclature, which helps to avoid complications and misunderstandings. Also, the phylogenies of potential fungal BCAs are shown and discussed.

A variety of biological control methods are available nowadays, however they should be further developed before they can be effectively adopted. Therefore, this study makes a significant contribution for a greater understanding in developing such approaches with the help of the detailed information presented.

## Materials and Methods

### Data Collection and Presentation

Research data on fungal antagonists used against fungal plant pathogens were collected from resources published during the past fifty years. The collected data are summarized in the **Supplementary Table 1**, which includes information on *Fungal* *biocontrol agent*, *Disease and host* as well as *Pathogen*. The disease or the pathogen suspension/control rate equal to or greater than 50% in each case is indicated with an asterisk (*) after the pathogen. Several potential fungal-like taxa causing plant diseases have also been taken into account to show the broad spectrum of activity of fungal BCAs and they are indicated with ^@^. Different applications and treatment methods (*in vitro* and *in vivo* or both) have often been used when evaluating the effect of fungal BCAs in the research papers we considered. Thus, the disease/pathogen suppression percentage was accounted based on the maximum inhibition shown (if different conditions were applied).

### Phylogenetic Analysis of Fungal Antagonists

Sequence data of the 28S nrRNA gene (LSU) from ex-type, ex-epitype, or ex-neotype strains of fungal antagonists listed in this study were downloaded from the NCBI’s GenBank nucleotide database (**Supplementary Table 2**). If no ex-type strains were available, sequences from voucher, authentic or reference strains were included in the analysis. *Hyphochytrium catenoides* (EF594059) was chosen as the outgroup taxon.

Sequences were aligned with Bioedit 7.1.3.0 (Hall, 1999), and the consensus sequences were further improved with MUSCLE implemented in MEGA 5v (Tamura et al., 2011). Alignments were checked and optimized manually when necessary. The phylogenetic tree was generated by maximum likelihood (ML) criterion using RAxML-HPC2 BlackBox (8.2.10) (Stamatakis, 2006; Stamatakis et al., 2008) on the CIPRES Science gateway portal V 3.3 (Miller et al., 2010) and a Bayesian analysis was performed with MrBayes v. 3.2.6 (Ronquist and Huelsenbeck, 2003). The general time-reversible model of evolution including estimation of invariable sites and assuming a discrete gamma distribution with default parameters was used for the ML analysis. The model of evolution (GTR + I + G) was determined with MrModeltest 2.2 (Nylander, 2004) under the Akaike Information Criterion (AIC) implemented in PAUP v. 4.0b10. Bayesian inference (Rannala and Yang, 1996; Zhaxybayeva and Gogarten, 2002) was determined by Markov Chain Monte Carlo sampling (MCMC) in MrBayes v. 3.0b4 (Huelsenbeck and Ronquist, 2001). Six simultaneous Markov chains were run for 7,000,000 generations and trees were sampled every 100^th^ generation. The first 20% of the trees (14000), representing the burn-in phase of the analysis, were discarded, while the remaining trees were used to calculate posterior probabilities (PP) in the majority rule consensus tree. The best scoring RAxML tree was selected and visualized with MEGA v. 5 (Tamura et al., 2011) and the graphical layout of the tree was created using PowerPoint 2010 version. ML Bootstrap support values (MLBS) greater than or equal to 50% and Bayesian posterior probabilities (PP) greater than or equal to 0.90 are indicated at the nodes of the branches. Alignments were deposited in TreeBASE (www.treebase.org) under the submission number 26869.

## Results

### Phylogenetic Analysis and Taxonomy of Fungal Antagonists

**Figure 2:**
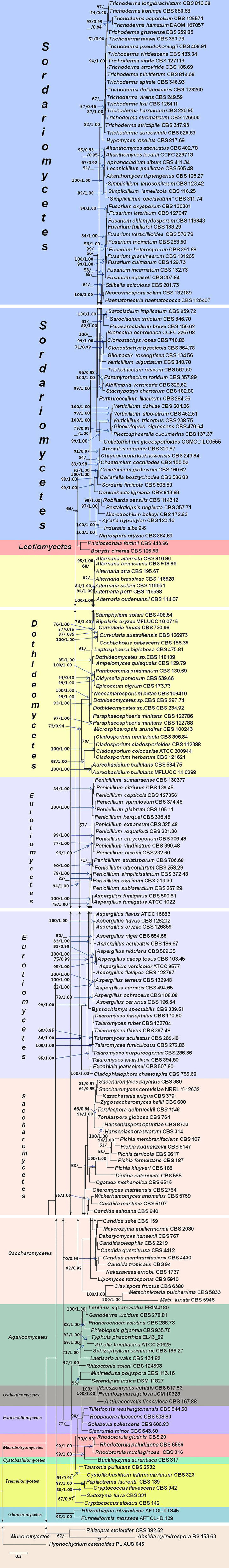
Phylogram resulting from maximum likelihood (RAxML) analysis of sequence alignment of the 28S nrRNA gene (LSU) sequences of fungal antagonists. ML bootstrap values (MLBS) ≥ 50% and Bayesian posterior probabilities (PP) ≥ 0.90 are at each node. The tree was rooted to *Hyphochytrium catenoides* (PL AUS 045). Classes are indicated with coloured blocks to the left of the tree.

After alignment the LSU dataset consisted of 1,030 characters (including alignment gaps) for 218 ingroup taxa and the outgroup taxon. The Bayesian tree had had a topology identical to the ML tree presented. (data not shown). Fungal BCAs are distributed in four phyla (Ascomycota, Basidiomycota, Glomeromycota and Mucoromycota) and thirteen classes *viz*. Sordariomycetes, Dothideomycetes, Eurotiomycetes, Leotiomycetes, Saccharomycetes (Ascomycota), Agaricomycetes, Exobasidiomycetes, Ustilaginomycetes, Microbotryomycetes, Cystobasidiomycetes, Tremellomycetes (Basidiomycota), Glomeromycetes (Glomeromycota), Mucoromycetes (Mucoromycota) in the kingdom fungi (**Figure 2**). The results show the phylogenetic placement of the various fungal BCAs within the kingdom fungi and confirm the current taxonomic placement of those BCAs. Most of the fungal BCAs belong to the class Sordariomycetes.

### Fungal Antagonists and Their Potential Against Plant Pathogens

Approximately 300 species or varieties belonging to 113 fungal genera are identified as BCAs for plant fungal pathogens based on the previous studies (**Supplementary Table 1**). Nine genera are recognized as potential genera, which consist of five or more known antagonistic species (**Table 2**). They are *Alternaria*, *Aspergillus*, *Candida*, *Fusarium*, *Penicillium*, *Pichia*, *Talaromyces*, *Trichoderma*, and *Verticillium*. *Trichoderma* is the most prominent genus comprising 25 BCAs and they are widely used in controlling plant diseases caused by fungi. *Alternaria*, *Botrytis*, *Colletotrichum*, *Fusarium*, *Lasiodiplodia*, *Penicillium*, *Phytophthora*, *Sclerotinia*, and *Verticillium* are identified as common and most abundant plant pathogenic genera, to which frequently BCAs are applied for disease control.

**Table 2:** Number of known fungal species in each genus with a potential Biocontrol Agent (BCA) activity against plant fungal pathogens.

Phylum	Class	Genus	Number of known species with a potential BC activity against plant fungal pathogens
**Ascomycota**	Dothideomycetes	***Alternaria***	**08**
		*Ampelomyces*	01
		*Aureobasidium*	01
		*Bipolaris*	01
		*Cladosporium*	04
		*Curvularia*	03
		*Didymella*	01
		*Epicoccum*	01
		*Leptosphaeria*	01
		*Microsphaeropsis*	02
		*Neocamarosporium*	01
		*Paraboeremia*	01
		*Paraphaeosphaeria*	01
		*Phaeotheca*	01
		*Stemphylium*	01
	Eurotiomycetes	***Aspergillus***	**16**
		*Cladophialophora*	01
		*Exophiala*	01
		*Paecilomyces*	01
		***Penicillium***	**17**
		***Talaromyces***	**08**
	*Incertae sedis*	*Gonatobotryum*	01
		*Teratosperma*	01
	Leotiomycetes	*Botrytis*	01
		*Phialocephala*	01
		*Cadophora*	01
	Saccharomycetes	***Candida***	**08**
		*Citeromyces*	01
		*Debaryomyces*	01
		*Diutina*	01
		*Hanseniaspora*	02
		*Kazachstania*	01
		*Lipomyces*	01
		*Metschnikowia*	03
		*Meyerozyma*	02
		*Nakazawaea*	01
		*Ogataea*	01
		***Pichia***	**05**
		*Saccharomyces*	02
		*Torulaspora*	02
		*Wickerhamomyces*	01
		*Zygosaccharomyces*	01
	Sordariomycetes	*Acremonium*	02
		*Akanthomyces*	03
		*Albifimbria*	01
		*Aphanocladium*	01
		*Arcopilus*	01
		*Bionectria*	01
		*Coniochaeta*	01
		*Chaetomium*	03
		*Clonostachys*	02
		*Collariella*	01
		*Colletotrichum*	02
		***Fusarium***	**12**
		*Gibellulopsis*	01
		*Gliomastix*	01
		*Haematonectria*	01
		*Hypomyces*	01
		*Lecanicillium*	01
		*Metapochonia*	01
		*Metarhizium*	01
		*Microdochium*	01
		*Muscodor*	03
		*Neocosmospora*	01
		*Nigrospora*	01
		*Paramyrothecium*	01
		*Parasarocladium*	01
		*Pestalotiopsis*	01
		*Plectosphaerella*	01
		*Purpureocillium*	01
		*Robillarda*	01
		*Sarocladium*	01
		*Simplicillium*	02
		*Sordaria*	01
		*Stachybotrys*	01
		*Stilbella*	01
		***Trichoderma***	**25**
		*Trichothecium*	01
		***Verticillium***	**05**
		*Xylaria*	01
**Basidiomycota**	Agaricomycetes	*Athelia*	01
		*Ganoderma*	01
		*Laetisaria*	01
		*Lentinus*	01
		*Minimedusa*	01
		*Phlebiopsis*	01
		*Rhizoctonia*	01
		*Schizophyllum*	01
		*Serendipita*	01
		*Trametes*	01
		*Typhula*	01
		*Waitea*	01
	Cystobasidiomycetes	*Buckleyzyma*	01
	Exobasidiomycetes	*Gjaerumia*	01
		*Robbauera*	01
		*Tilletiopsis*	02
	Microbotryomycetes	*Rhodotorula*	03
	Tremellomycetes	*Cystofilobasidium*	01
		*Naganishia*	01
		*Papiliotrema*	02
		*Saitozyma*	01
		*Tausonia*	01
	Ustilaginomycetes	*Anthracocystis*	01
		*Moesziomyces*	02
**Glomeromycota**	Glomeromycetes	*Claroideoglomus*	01
		*Diversispora*	01
		*Funneliformis*	01
		*Gigaspora*	01
		*Rhizophagus*	03
		*Septoglomus*	01
		*Simiglomus*	01
**Mucoromycota**	Mucoromycetes	*Absidia*	01
		*Rhizopus*	01

Potential antagonistic genera are highlighted in boldface.

## Discussion

The present study provides a comprehensive list of fungal BCAs used against a wide range of fungal plant pathogens, and establishes their phylogenetic relationships using a phylogenetic analysis based on the available authentic 28S nrRNA gene (LSU) sequence data. This will help clarify the currently correct names for the species since they have often been wrongly quoted and interpreted in the literature of biocontrol. It will further help with searches for the species in earlier literature where the old names were used.

In the phylogenetic analysis presented in this review, fungal BCAs were distributed in thirteen classes *viz*. Sordariomycetes, Dothideomycetes, Eurotiomycetes, Leotiomycetes, Saccharomycetes (Ascomycota), Agaricomycetes, Exobasidiomycetes, Ustilaginomycetes, Microbotryomycetes, Cystobasidiomycetes, Tremellomycetes (Basidiomycota), Glomeromycetes (Glomeromycota), Mucoromycetes (Mucoromycota) in the kingdom fungi (**Figure 2**). The results confirmed the taxonomic placement of the various fungal BCAs and most of the fungal BCAs belong to the class Sordariomycetes. However, DNA sequences for some of the species are not currently available and therefore it is essential to have sequence data from authentic strains to confirm their taxonomy.

The main and common issue that we found when preparing this review was the method used by the authors to identify the fungal antagonists. Most of the publications have not used an appropriate identification method and they mainly followed the traditional identification methods and resources. However, molecular DNA sequencing and phylogenetic tools have been used in some of the recent publications to identify the antagonists and the pathogen correctly (Grondona et al., 1997; Rubini et al., 2005; Sriram and Poornadchanddra, 2013; Hung et al., 2015; Kheireddine et al., 2018). In addition, the nomenclature and classification of several fungal species have been subjected to change during the past few decades due to the modern taxonomic approaches and views. Therefore, corrections on those changes are mandatory in order to avoid misinterpretations. In this study, we have given the current names of the fungal species and the names commonly used in each particular research paper are mentioned in the brackets where applicable (**Table 2**).

*Trichoderma* is a species rich, asexual genus in the family *Hypocreaceae* (Hypocreales, Sordariomycetes) and currently includes 433 species epithets in Index Fungorum, but DNA sequence data for most of the species are not available in GenBank. The sexual morphs of *Trichoderma* species are linked to *Hypocrea*. Members of the genus *Trichoderma* are biotrophic, hemibiotrophic, saprobic or hypersaprobic on various plants, or other fungi (Maharachchikumbura et al., 2016) and have been identified as the antagonists with greatest potential. They have been employed in several applications in plant fungal disease control. Twenty-five known *Trichoderma* species are included in **Table 2** and these species have great potential for significantly controlling more than 100 fungal plant pathogens worldwide. Out of these species *Trichoderma harzianum* can be considered as the most common and commercially developed BCA used for a wide range of plant fungal diseases. *Trichoderma* species produce a number of metabolites and these metabolites play a major role in biological control mechanisms (Reino et al., 2008). Apart from that, *Aspergillus* and *Penicillium* species also play a vital role as BCAs next to *Trichoderma* (**Supplementary Table 1** and **Table 2**).

Some *fungus-like* genera (*Globisporangium*, *Hyphochytrium* and *Pythium*) belonging to Oomycota, Chromista, are also important as BCAs and have been used to control plant fungal diseases (**Table 3**). Considering the activity of *fungus-like* BCAs, *Pythium oligandrum* may be considered as the one with the greatest potential as a BCA.

**Table 3:** Fungal-like species (Oomycota, Chromista) used as Biocontrol Agents (BCAs) in the past few decades against fungal pathogens/diseases of different host plants.

Biocontrol agent	Disease and host	Pathogen	References
*Hyphochytrium catenoides*	Phytophthora root rot of soybean	*Phytophthora megasperma* f. sp. *glycines*	Filonow and Lockwood, 1985
*Globisporangium ultimum* (*Pythium ultimum*)	Barley powdery mildew	*Blumeria graminis* f. sp. *hordei*	Haugaard et al., 2001
*Pythium acanthicum*	Damping-off of cucumber	*Globisporangium ultimum* (*Pythium ultimum*)	Ali-Shtayeh and Saleh, 1999
	Fusarium ear blight of wheat	*Fusarium culmorum* & *Microdochium nivale*	Diamond and Cooke, 2003
*Pythium nunn*	Damping-off disease of cucumber	*Globisporangium ultimum* (*Pythium ultimum*)	Paulitz and Baker, 1987
*Pythium oligandrum*	Seedling and taproot diseases of sugar beet	*Aphanomyces cochlioides*	Takenaka and Ishikawa, 2013
*** ***	Seedling disease of sugar beet	*Aphanomyces cochlioides*	Takenaka et al., 2006
*** ***	Grey mould of grapevine	*Botrytis cinerea*	Mohamed et al., 2007
*** ***	Grey mould of tomato	*Botrytis cinerea*	Le Floch et al., 2003
*** ***	Grey mould of strawberry	*Botrytis cinerea*	Meszka and Bielenin, 2010
*** ***	Cercospora leaf spot in sugar beet	*Cercospora beticola*	Takenaka and Tamagake, 2009
*** ***	Foot rot pathogens of pea	*Didymella pinodella* (*Phoma medicaginis* var. *pinodella*)	Bradshaw-Smith et al., 1991
*** ***	Fusarium head blight	*Fusarium graminearum*	Takenaka et al., 2003
*** ***	Fusarium crown and root rot of tomato	*Fusarium oxysporum* f. sp. *radicis-lycopersici*	Benhamou et al., 1997; Gerbore et al., 2014
*** ***	Seed rot of tomato	*Globisporangium ultimum* (*Pythium ultimum*)	He et al., 1992; Gerbore et al., 2014
*** ***	Pythium damping-off in cress and sugar-beet	*Globisporangium ultimum* (*Pythium ultimum*)	Vesely, 1977; McQuilken et al., 1990; McQuilken et al., 1992
*** ***	Damping-off disease of cress	*Globisporangium ultimum* (*Pythium ultimum*)	Al-Hamdani et al., 1983
*** ***	Damping-off of cucumber	*Globisporangium ultimum* (*Pythium ultimum*)	Ali-Shtayeh and Saleh, 1999
	Seedling diseases of cotton	*Globisporangium ultimum* (*Pythium ultimum*)	Martin and Hancock, 1986; Gerbore et al., 2014
*** ***	Pre-emergence damping-off disease of sugar beet	*Globisporangium ultimum* (*Pythium ultimum*)	Martin and Hancock, 1987
*** ***	Pythium root rot of tomato	*Pythium dissotocum*	Vallance et al., 2009; Gerbore et al., 2014
*** ***	Crown and root rot of tomato	*Phytophthora parasitica* (*Phytophthora nicotianae*)	Picard et al., 2000
*** ***	Damping-off of wheat	*Pythium ultimum* var. *ultimum*	Abdelzaher et al., 1997
*** ***	Leaf spot of strawberry	*Ramularia grevilleana* (*Mycosphaerella fragariae*)	Meszka and Bielenin, 2010
*** ***	Damping off disease of tomato	*Rhizoctonia solani*	He et al., 1992; Gerbore et al., 2014
*** ***	Black scurf of potato	*Rhizoctonia solani*	Ikeda et al., 2012
*** ***	Seedling disease of sugar beet	*Rhizoctonia solani*	Takenaka et al., 2003
*** ***	Powdery mildew of strawberry	*Sphaerotheca macularis* (*Podosphaera aphanis*)	Meszka and Bielenin, 2010
*** ***	Verticillium wilt of pepper	*Verticillium dahliae*	Al-Rawahi and Hancock, 1998; Rekanovic et al., 2007
	Verticillium wilt of olive	*Verticillium dahliae*	Varo et al., 2016
*Pythium periplocum*	Grey mould of grape-vine	*Botrytis cinerea*	Paul, 1999a
* *	Damping-off of cucumber	*Globisporangium ultimum* (*Pythium ultimum*)	Ali-Shtayeh and Saleh, 1999
*Pythium radiosum*	Grey mould of grape-vine	*Botrytis cinerea*	Paul, 1999b

Application of one or more biocontrol agents combined with physical and/or chemical control treatments can be considered as a useful strategy in achieving an enhanced performance against plant diseases. Sometimes, it is difficult to select individual strains with a broad spectrum of activity against several plant pathogens that cause severe damages and infections on plants or fruits. Mixed cultures of the microbial antagonists appear to provide better control of plant diseases over individual strains (Guetsky et al., 2001; Freeman et al., 2004; Xu et al., 2011) and these BCAs should be compatible and applied properly with a correct formulation. The application of antagonist mixtures improves the efficacy of biocontrol and are used in many agricultural fields including post-harvest disease control systems. (Datnoff et al., 1995; Nagtzaam et al., 1998; Guetsky et al., 2001; Janisiewicz and Korsten, 2002; Freeman et al., 2004; Droby, 2006; Xu et al., 2011; Doley et al., 2017; Nabi et al., 2017). In a study of the biological control of Armillaria root rot of strawberry plants by Raziq and Fox (2005), all the strawberry plants treated with *Trichoderma* *harzianum* isolates or *T. viride* isolate alone died by the end of the experiment, while 50% of them survived when treated with a combination of any of the antagonists with *Dactylium dendroides*. Calvo et al. (2003) improved the biocontrol efficiency of postharvest diseases of apples (*Penicillium expansum* and *Botrytis cinerea*) by using mixtures of yeasts (*Rhodotorula glutinis*, *Cryptococcus albidus* and *C. laurentii*) without increasing the amount of antagonists applied. Haggag and Nofal (2006) revealed that the multi-biocontrol agents namely *Trichoderma koningii*, *T. hamatum*, *Pseudomonas fluorescens*, *P. putida*, *Tilletiopsis minor*, and *T. washingtonensis* were more effective against *Botryodiplodia* disease (*Lasiodiplodia theobromae* = Botryodiplodia theobromae) on some Annona cultivars when applied in combination than when applied individually or even when applied in any combination of two agents. Furthermore, applications of multi-biocontrol agents resulted in a significant increase of fruit yield. Doley et al. (2017) showed that the incidence of stem-rot in groundnut caused by *Sclerotium* *rolfsii* was significantly lowered by combined inoculation of both *Glomus* *fasciculatum* along with *Trichoderma* *viride* as compared to when either *G. fasciculatum* or *T. viride* was applied alone.

Application of BCAs with inorganic and organic substances and chemicals such as bicarbonates and chlorides of alkali metals, minerals and other elements (Piano et al., 1997; Droby et al., 1997; Tian et al., 2002a; Gamagae et al., 2003; Tian et al., 2005; Karabulut et al., 2005; Cao et al., 2012) or low dosage of fungicides (Chand-Goyal and Spotts, 1996; Spotts et al., 1998; Qin and Tian, 2004; De Cal et al., 2009) are widely used to control plant pathogenic diseases including post-harvest diseases. A few potential examples of these applications are indicated here. Thus Tian et al. (2005) investigated the synergistic biocontrol effects of *Cryptococcus laurentii* and *Rhodotorula glutinis* combined with silicon (Si) against *Alternaria alternata* and *Penicillium expansum* moulds in jujube fruit stored at different temperatures and found that combinations of these two biocontrol agents with 2% Si is most effective in controlling the diseases on jujube fruit stored at 20°C. The studies of Karabulut et al. (2005) demonstrated that postharvest applications of sodium bicarbonate within a hydrocooler significantly controlled postharvest diseases of sweet cherries. Liu et al. (2010) revealed that tea polyphenol alone or in combination with biocontrol agents has great potential in commercial management of postharvest diseases in fruits. Larena et al. (2010) enhanced the adhesion of *Epicoccum nigrum* conidia to peach surfaces by adding 2.5% methylcellulose to the conidial formulation of *E. nigrum* and this improved the biocontrol of brown rot caused by *Monilinia laxa*. Cao et al. (2012) found that Boron improves biocontrol activity of *Cryptococcus laurentii* against *Penicillium expansum* in jujube fruit. The study by Gamagae et al. (2003) showed that the use of sodium bicarbonate at 2% with the biocontrol agent *Candida oleophila* reduces anthracnose caused by *Colletotrichum gloeosporioides* on papaya during storage. Sometimes, these integrated applications increase the efficiency of the particular BCA and sometimes indirectly improve plant productivity. Ordentlich et al. (1990) showed that integrated treatment of the fungicide captan and the biocontrol agent *Trichoderma harzianum* resulted in a reduction of *Verticillium dahlia* colonization of potato stems (Verticillium wilt), increasing marketable and total potato yield of cultivars Draga by 84% and 46% respectively, and total yield of cultivar Desiree by 80%. However, only BCA alone applications (without combining with organic or inorganic chemical substances) are considered in this review.

Fungal biological control agents act through several mechanisms (*see the introduction*) when controlling plant diseases. However, these mechanisms may cause risks to non-target species including mycorrhizal and saprophytic fungi, soil bacteria, other plants, insects, aquatic and terrestrial animals, and humans. Possible non-target effects of any BCAs or method used in the field should be determined before their applications. Continuous monitoring and the use of molecular techniques to identify and follow the movement of BCAs are also necessary and negative biological impacts can then be avoided (Brimner and Boland, 2003). Cross-protection can be defined as the protection conferred on a host by infection with one strain of a microorganism that prevents infection by a closely related strain of that microorganism. This method has been widely used to control the plant diseases caused by *Fusarium* and *Verticillium* species (Matta and Garibaldi, 1977; Larkin and Fravel, 1998; Nel et al., 2006; Zhu et al., 2013).

The efficiency of a particular BCA against plant diseases can be altered by many factors such as; environmental factors, time of treatment, season of the application, nature or technique of the treatment and the frequency of the application (McLaughlin et al., 1990; Wu and Hsiang, 1998; Ali-Shtayeh and Saleh, 1999; Schisler et al., 2002; Bhagat and Pan, 2007; Lal et al., 2009; Perelló et al., 2009; Padder and Sharma, 2011). The same BCA can show different efficiencies at *in vitro*, *in vivo*, and greenhouse/field conditions (Wokocha et al., 1986; Larena et al., 2003; Campanile et al., 2007; Padder and Sharma, 2011; Ommati et al., 2013). The dual culture method is the most widely used and simplest *in vitro* technique to determine the activity of BCAs against pathogens and sometimes the results obtained from this method may differ from results found in the *in vivo*, field or greenhouse conditions (Padder and Sharma, 2011; Zheng et al., 2011; Kheireddine et al., 2018). Therefore, it is hard to expect the same result for the same BCA in the field compared to the laboratory or greenhouse applications and it is essential to carry out tests in both *in vitro* and *in vivo* before scaling up the particular BCA. However, in this review, the maximum disease control situations are stipulated in **Table 2**, in case where different environmental conditions have been used in the respective publication.

## Future Aspects

An assay based on modern taxonomic approaches is highly recommended to identify the antagonists and the pathogens correctly. Also, verified antagonistic cultures must be obtained from reputable culture collections in a case when researchers are planning to use known strains. These practices will largely minimize the confusion related to this field in the future. Moreover, modern biotechnological and genetic engineering tools have contributed immensely towards the development of new fungal strains with high capacity and efficiency of biocontrolling. Last, but not least, fungal extracts and secondary metabolites produced by various fungal species (Mathivanan et al., 1998; Park et al., 2005; Pal and McSpadden Gardener, 2006; Koitabashi and Tsushima, 2007; Reino et al., 2008; Stoppacher et al., 2010; Lou et al., 2011; Cimmino et al., 2013) will also play a significant role in future bio-control methods of plant pathogens.

## Author Contributions

KT contributed to conception and design of the study. DD and KT performed the phylogenetic analysis. AP, SK, and IP wrote sections of the manuscript. All authors contributed to the article and approved the submitted version.

## Conflict of Interest

The authors declare that the research was conducted in the absence of any commercial or financial relationships that could be construed as a potential conflict of interest. 
