# Supplementary material for: Fungi vs. Fungi in Biocontrol: An Overview of Fungal Antagonists Applied Against Fungal Plant Pathogens
Source: Front Cell Infect Microbiol. 2020 Nov 30;10:604923. doi: 10.3389/fcimb.2020.604923 (PMC7734056; doi:10.3389/fcimb.2020.604923)
Supplement: Supplementary file 1 [file Table_1.doc]

**Supplementary Table 1.** Fungal BCAs used in the past few decades against fungal pathogens/diseases of different host plants.

| **Biocontrol agent** | **Disease and host** | **Pathogen** | **References** |
| --- | --- | --- | --- |
| *Absidia cylindrospora* | Karnal bunt of wheat | *Tilletia indica** | Sharma and Basandrai 2000 |
| *Acremonium obclavatum* | Rust of groundnut | *Puccinia arachidis** | Gowdu et al. 1993 |
| *Acremonium sp.*CEF-193 | Verticillium wilt of cotton | *Verticillium dahliae* | Li et al. 2014 |
| *Akanthomyces attenuatus* (*Lecanicillium attenuatum*) | Downy mildew of cucumber | *Podosphaera fuliginea* (*Sphaerotheca fuliginea*)*** | Kim et al. 2007a |
| *Akanthomyces dipterigenus* (*Lecanicillium longisporum*) | Downy mildew of cucumber | *Podosphaera fuliginea* (*Sphaerotheca fuliginea*)*** | Kim et al. 2007a |
| *Akanthomyces lecanii* (*Lecanicillium lecanii*) | Coffee leaf rust | *Hemileia vastatrix* | Vandermeer et al. 2009, Jackson et al. 2012 |
|  | Microcyclic rust of chrysanthemum/Chrysanthemum white rust | *Puccinia horiana* | Srivastava et al. 1985 |
|  | Powdery mildew of cucumber | *Podosphaera fuliginea* (*Sphaerotheca fuliginea*)*** | Verhaar and Hijwegen 1993, Askary et al. 1998 |
|  | Powdery mildew of rose | *Podosphaera pannosa* (*Sphaerotheca pannosa* var. *rosae*)* | Verhaar et al. 1999 |
| *Albifimbria verrucaria* (*Myrothecium verrucaria*) | White mould of bean | *Sclerotinia sclerotiorum** | Boland and Inglis 1989 |
| *Alternaria alternata* | Tan spot of wheat | *Pyrenophora tritici-repentis** | Li and Sutton 1995 |
|  | Verticillium wilt of cotton | *Verticillium dahliae* | Li et al. 2014 |
|  | White mould of bean | *Sclerotinia sclerotiorum* | Boland and Inglis 1989 |
| *Alternaria atra* (*Ulocladium atrum*) | Neck rot of onion | *Botrytis aclada** | Köhl 2004 |
|  | Grey mould of strawberry, pot roses & cyclamen | *Botrytis cinerea** | Kessel et al. 2001, Köhl 2004 |
|  | Leaf spots of onion | *Botrytis cinerea** | Köhl 2004 |
|  | Leaf blight of onion | *Botrytis squamosa** | Köhl 2004 |
|  | Crown rot of banana | *Lasiodiplodia theobromae* | Alvindia and Natsuaki 2008 |
| *Alternaria brassicae* | Verticillium wilt of cotton | *Verticillium dahliae* | Li et al. 2014 |
| *Alternaria compacta* | Verticillium wilt of cotton | *Verticillium dahliae* | Li et al. 2014 |
| *Alternaria oudemansii* (*Ulocladium oudemansii*) | Botrytis bunch rot of grape | *Botrytis cinerea** | Reglinski et al. 2005, 2010 |
| *Alternaria porri* | Verticillium wilt of cotton | *Verticillium dahliae* | Li et al. 2014 |
| *Alternaria solani* | Verticillium wilt of cotton | *Verticillium dahliae* | Li et al. 2014 |
| *Alternaria* sp. CEF-833 | Verticillium wilt of cotton | *Verticillium dahliae* | Li et al. 2014 |
| *Alternaria tenuissima* | Verticillium wilt of cotton | *Verticillium dahliae** | Li et al. 2014 |
| *Ampelomyces quisqualis* | Powdery mildew of grape | *Erysiphe necator* (*Uncinula necator*)*** | Falk et al. 1995a, b |
|  | Powdery mildew of spindle (*Euonymus* species) | *Erysiphe euonymi-japonici* (*Oidium euonymi-japonici*)*** | Ahanger et al. 2018 |
|  | Powdery mildew of mango | *Oidium mangiferae** | Sztejnberg et al. 1989, Kiss et al. 2004 |
|  | Powdery mildew of strawberry | *Podosphaera aphani, P. macularis* (*Sphaerotheca macularis*) | Pertot et al. 2008 |
|  | Powdery mildew of cucumber | *Podosphaera fuliginea* (*Sphaerotheca fuliginea*)*** | Sztejnberg et al. 1989, Stirling and Stirling 1997 |
|  |  | *Podosphaera fusca* (*Sphaerotheca fusca*)*** | Elad et al. 1998 |
|  | Powdery mildew of apple | *Podosphaera leucotricha** | Bosshard et al. 1987 |
|  | Powdery mildew of rose | *Podosphaera pannosa* (*Sphaerotheca pannosa* var. *rosae*)*** | Verhaar et al. 1999 |
|  | Powdery mildew of cashew | *Pseudoidium anacardii* (*Oidium anacardii*)*** | Dominic and Marthamakobe 2017 |
| *Anthracocystis flocculosa* (*Sporothrix flocculosa*) | Powdery mildew of cucumber | *Podosphaera fuliginea* (*Sphaerotheca fuliginea*) | Dik et al. 1998 |
| *Aphanocladium album* | Powdery mildew of rose | *Podosphaera pannosa* (*Sphaerotheca pannosa var. rosae*)* | Verhaar et al. 1999 |
|  | Microcyclic rust of chrysanthemum | *Puccinia horiana* | Srivastava et al. 1985 |
| *Arcopilus cupreus* (*Chaetomium cupreum*) | Thielaviopsis bud rot of bottle palm | *Ceratocystis paradoxa* (*Thielaviopsis paradoxa*)*** | Soytong et al. 2005 |
|  | Root rot of pomelo | *Phytophthora palmivora** | Hung et al. 2015 |
|  | White root disease of rubber | *Rigidoporus microporus** | Kaewchai and Soytong 2010, Soytong and Kaewchai 2014 |
| *Aspergillus aculeatus* | Verticillium wilt of cotton | *Verticillium dahliae** | Li et al. 2014 |
|  | Verticillium wilt of eggplant | *Verticillium dahliae** | Marois et al. 1982 |
| *Aspergillus caespitosus* | Crown rot of banana | *Fusarium fujikuroi* (*Fusarium verticillioides*) | Alvindia and Natsuaki 2008 |
|  | Crown rot of banana | *Lasiodiplodia theobromae* | Alvindia and Natsuaki 2008 |
| *Aspergillus carneus* | Damping-off disease of Egyptian clover | *Pythium spinosum**@ | Maghazy et al. 2008 |
| *Aspergillus cervinus* | Damping-off disease of Egyptian clover | *Pythium spinosum**@ | Maghazy et al. 2008 |
| *Aspergillus flavipes* | Sclerotinia stem rot of oilseed rape | *Sclerotinia sclerotiorum** | Zhang et al. 2014 |
|  | Red rot of sugarcane | *Colletotrichum falcatum** | Suresh and Nelson 2016 |
| *Aspergillus flavus* | Wilting, yellowing or necrosis of peach | *Monilinia laxa* | Melgarejo et al. 1986 |
| *Aspergillus fumigatus* | Red rot of sugarcane | *Colletotrichum falcatum** | Suresh and Nelson 2016 |
|  | Leaf smut of cowpea | *Erratomyces patelii* (*Protomycopsis phaseoli*)*** | Adejumo et al. 1999 |
| *Aspergillus galapagensis* (*Neosartorya galapagensis*) | Red root disease of rubber | *Ganoderma philippii* (*Ganoderma pseudoferreum*)*** | Ogbebor et al. 2010 |
| *Aspergillus nidulans* | White root disease of rubber | *Rigidoporus microporus** | Ogbebor et al. 2015 |
| *Aspergillus nidulans* | Red root disease of rubber | *Ganoderma philippii* (*Ganoderma pseudoferreum*)*** | Ogbebor et al. 2010 |
| *Aspergillus nidulans* (*Emericella nidulans*) | Anthracnose of vanilla | *Colletotrichum gloeosporioides** | Talubnak and Soytong 2010 |
| *Aspergillus niger* | Leaf spot disease of toothache plant (*Spilanthes oleracea*) | *Alternaria alternata** | Thakur and Harsh 2014 |
|  | Anthracnose of olive | *Colletotrichum acutatum** | Landum et al. 2016 |
|  | Red rot of sugarcane | *Colletotrichum falcatum** | Suresh and Nelson 2016 |
|  | Corynespora leaf disease of rubber | *Corynespora cassiicola** | Evueh and Osemwegie 2011 |
|  | Red root disease of rubber | *Ganoderma philippii* (*Ganoderma pseudoferreum*)*** | Ogbebor et al. 2010 |
|  | Inflorescence blight of cashew | *Lasiodiplodia theobromae** | Adeniyi et al. 2013 |
|  | White root disease of rubber | *Rigidoporus microporus** | Kaewchai and Soytong 2010, Ogbebor et al. 2015 |
|  | Verticillium wilt of cotton | *Verticillium dahliae* | Li et al. 2014 |
| *Aspergillus ochraceus* | Fusarium crown and root rot of tomato | *Fusarium oxysporum* f. sp. *radicis-lycopersici* | Marois et al. 1981 |
| *Aspergillus ochraceus* | Red root disease of rubber | *Ganoderma philippii* (*Ganoderma pseudoferreum*)*** | Ogbebor et al. 2010 |
| *Aspergillus oryzae* | Verticillium wilt of cotton | *Verticillium dahliae* | Li et al. 2014 |
| *Aspergillus sulphureus* | Red rot of sugarcane | *Colletotrichum falcatum** | Suresh and Nelson 2016 |
|  | Damping-off disease of Egyptian clover | *Pythium spinosum**@ | Maghazy et al. 2008 |
| *Aspergillus terreus* | Pestalotia leaf spots of guava | *Pestalotia psidii** | Pandey et al. 1993 |
| *Aspergillus turcosus* | Red root disease of rubber | *Ganoderma philippii* (*Ganoderma pseudoferreum*)*** | Ogbebor et al. 2010 |
| *Aspergillus versicolor* | Red root disease of rubber | *Ganoderma philippii* (*Ganoderma pseudoferreum*)*** | Ogbebor et al. 2010 |
|  | Powdery scab of potato | *Spongospora subterranea** | Nakayama and Sayama 2013, O’Brien 2017 |
| *Athelia bombacina* | Scab disease of apple | *Venturia inaequalis* | Stirling and Stirling 1997 |
| *Aureobasidium pullulan* | Brown rot of stone fruit | *Monilinia laxa** | Rungjindamai et al. 2013 |
|  | Postharvest rot of grape | *Monilinia laxa** | Schena et al. 2003 |
|  | Brown rot of peach | *Monilinia laxa**, *M. fructigena**, *M. fructicola** | Mari et al. 2012, Di Francesco et al. 2017 |
|  | Blue mould of citrus | *Penicillium italicum** | Wilson and Chalutz 1989 |
|  | Sour rot of grape | *Aspergillus carbonarius** | Dimakopoulou et al. 2008 |
|  | Black mould of grape | *Aspergillus niger** | Schena et al. 1999 |
|  | Grey mould of apple | *Botrytis cinerea** | Schena et al. 1999, Ippolito et al. 2000, Kheireddine et al. 2018 |
|  | Grey mould/ postharvest rot of cherry | *Botrytis cinerea** | Schena et al. 2003 |
|  | Grey mould of grape, post-harvest bunch rot of table grape | *Botrytis cinerea** | Schena et al. 1999, Schena et al. 2003, Parafati et al. 2015 |
|  | Anthracnose leaf spots of guava | *Colletotrichum gloeosporioides** | Pandey et al. 1993 |
|  | Eutypa dieback of grapevine | *Eutypa lata ** | Munkvold and Marois 1993 |
|  | Brown rot blossom blight of sweet cherry | *Monilinia fructicola** | Wittig et al. 1997 |
|  | Green mould of grape | *Penicillium digitatum** | Schena et al. 1999 |
|  | Green mould of citrus | *Penicillium digitatum** | Wilson and Chalutz 1989 |
|  | Blue mould of apple | *Penicillium expansum** | Ippolito et al. 2000 |
|  | Pestalotia leaf spots of guava | *Pestalotia psidii** | Pandey et al. 1993 |
|  | Rhizopus rot of grape | *Rhizopus stolonifer** | Schena et al. 1999 |
|  | Rhizopus rot of cherry tomato | *Rhizopus stolonifer** | Schena et al. 1999 |
|  | Verticillium wilt of olive | *Verticillium dahliae** | Varo et al. 2016 |
| *Bionectria ochroleuca* | Verticillium wilt of cotton | *Verticillium dahliae* | Li et al. 2014 |
| *Bipolaris oryzae* | Powdery mildew of barley | *Blumeria graminis* f. sp. *hordei** | Haugaard et al. 2001 |
| *Blastobotrys* sp. FP12 | Verticillium wilt of eggplant | *Verticillium dahliae** | Papasotiriou et al. 2013 |
| *Botrytis cinerea* | Brown rot blossom blight of stone fruits | *Monilinia fructicola** | Wittig et al. 1997 |
| *Buckleyzyma aurantiaca* (*Rhodotorula aurantiaca*) | Blue mould of apple | *Penicillium expansum** | Chand-Goyal and Spotts 1996a |
| *Cadophora orchidicola* (*Leptodontidium orchidicola*) | Verticillium wilt of tomato | *Verticillium dahliae* | Andrade-Linares et al. 2011 |
| *Candida maritima* | Stem-end rot of mango | *Lasiodiplodia theobromae** | Michereff et al. 1997 |
| *Candida membranifaciens* | Anthracnose of mango | *Colletotrichum gloeosporioides** | Kefialew and Ayalew 2008 |
| *Candida oleophila* | Grey mould of apple | *Botrytis cinerea** | El-Neshawy and Wilson 1997 |
|  | Grey mould rot of pear | *Botrytis cinerea** | Karabulut and Baykal 2004 |
|  | Anthracnose of papaya | *Colletotrichum gloeosporioides* | Gamagae et al. 2003 |
|  | Side rot of pear | *Cadophora malorum* (*Phialophora malorum*)***, *Cladosporium herbarum**, *Alternaria alternata** | Benbow and Sugar 1999 |
|  | Crown rot of banana | *Colletotrichum musae* | Lassois et al. 2008 |
|  | Sour rot decay of citrus | *Dipodascus geotrichum* (*Geotrichum candidum*)*** | Lahlali et al. 2004 |
|  | Crown rot of banana | *Fusarium fujikuroi* (*Fusarium moniliforme*)*** | Lassois et al. 2008 |
|  | Green mould of citrus | *Penicillium digitatum** | Lahlali et al. 2004, 2005 |
|  | Blue mould of pear | *Penicillium expansum** | El-Neshawy and Wilson 1997, Karabulut and Baykal 2004 |
|  | Blue mould of citrus | *Penicillium italicum** | Lahlali et al. 2004, 2005 |
| *Candida quercitrusa* | Anthracnose of chilli | *Colletotrichum truncatum* (*Colletotrichum capsici*)*** | Chanchaichaovivat et al. 2007 |
| *Candida saitoana* | Grey mould of apple | *Botrytis cinerea** | El Ghaouth et al. 2003 |
| *Candida sake* | Grey mould of apple | *Botrytis cinerea** | Vinas et al. 1998 |
|  | Stem end rot disease in kiwifruit | *Botrytis cinerea** | Cook et al. 1999 |
|  | Green mould of citrus | *Penicillium digitatum** | Arras et al. 1998 |
|  | Blue mould of pear | *Penicillium expansum** | Torres et al. 2006 |
|  | Blue mould rot of apple | *Penicillium expansum** | Usall et al. 2001, Morales et al. 2008 |
|  | Blue mould of apple | *Penicillium expansum** | Vinas et al. 1998 |
|  | Rhizopus rot of apple | *Rhizopus stolonifer* (*Rhizopus nigricans*)*** | Vinas et al. 1998 |
| *Candida tropicalis* | Anthracnose of mango | *Colletotrichum gloeosporioides** | Sriram and Poornadchanddra 2013 |
|  | Crown rot of banana | *Colletotrichum musae** | Zhimo et al. 2016 |
|  | Stem-end rot of mango | *Lasiodiplodia theobromae* (*Diplodia natalensis*)*** | Sriram and Poornadchanddra 2013 |
| *Ceratobasidium* spp. Q1M19, Q2M19, Q1M161.2 | Sheath blight of rice | *Rhizoctonia solani** | Mosquera-Espinosa et al. 2013 |
| *Chaetomium cochliodes* | Powdery mildew of apple | *Podosphaera leucotricha** | Bosshard et al. 1987 |
|  | Scab disease of apple | *Venturia inaequalis** | Bosshard et al. 1987 |
| *Chaetomium globosum* | Ascochyta blight of chickpea | *Ascochyta rabiei** | Rajakumar et al. 2005 |
|  | Spot blotch disease of wheat | *Bipolaris sorokiniana* (*Drechslera sorokiniana*)*** | Biswas et al. 2000, Aggarwal et al. 2004 |
|  | Grey mould of cannabis | *Botrytis cinerea** | Kusari et al. 2013 |
|  | Thielaviopsis bud rot of bottle palm | *Ceratocystis paradoxa* (*Thielaviopsis paradoxa*)*** | Soytong et al. 2005 |
|  | Cladosporium leaf spot | *Cladosporium cladosporioides** | Pradeep and Kumud 2000 |
|  | Anthracnose of chilli | *Colletotrichum truncatum* (*Colletotrichum capsici*)*** | Vasanthakumari and Shivanna 2013 |
|  | Anthracnose of tea | *Colletotrichum* sp.* | La et al. 2016 |
|  | Root rot of tea, coffee and rubber | *Fusarium* sp.* | La et al. 2016 |
|  | Witches' broom disease of cocoa | *Moniliophthora perniciosa* (*Crinipellis perniciosa*) | Krauss and Soberanis 2001 |
|  | Powdery mildew of apple | *Podosphaera leucotricha** | Bosshard et al. 1987 |
|  | Root rot of pomelo | *Phytophthora palmivora** | Hung et al. 2015 |
|  | Damping-off disease of Egyptian clover | *Pythium spinosum**@ | Maghazy et al. 2008 |
|  | Sclerotinia stem rot of oilseed rape | *Sclerotinia sclerotiorum** | Zhang et al. 2014 |
|  | Pink rot disease of cannabis | *Trichothecium roseum** | Kusari et al. 2013 |
|  | Verticillium wilt of cotton | *Verticillium dahliae** | Zheng et al. 2011, Li et al. 2014 |
|  | Scab disease of apple | *Venturia inaequalis* | Stirling and Stirling 1997 |
| *Chaetomium globosum* (*Chaetomium olivaceum*) | Armillaria root rot of apple | *Armillaria mellea* | Raziq and Fox 2004, 2006 |
| *Chrysocorona lucknowensis* (*Chaetomium lucknowense*) | Root rot of pomelo | *Phytophthora palmivora** | Hung et al. 2015 |
| *Chaetomium* sp. B132& CEF-681 | Verticillium wilt of cotton | *Verticillium dahliae* | Zheng et al. 2011 |
| *Citeromyces matritensis* | Grey mould of apple | *Botrytis cinerea** | Kheireddine et al. 2018 |
| *Cladophialophora chaetospira* (*Heteroconium chaetospira*) | Alternaria leaf spot of chinese cabbage | *Alternaria brassicae** | Morita et al. 2003 |
|  | Clubroot of cabbage | *Plasmodiophora brassicae** | Narisawa et al. 2000 |
|  | Verticillium wilt of eggplant | *Verticillium dahliae** | Narisawa et al. 2002 |
|  | Verticillium yellows of chinese cabbage | *Verticillium dahliae** | Narisawa et al. 2000, 2004 |
| *Cladosporium cladosporioides* | Anthracnose leaf spots of guava | *Colletotrichum gloeosporioides** | Pandey et al. 1993 |
| *Cladosporium colocasia* | Verticillium wilt of cotton | *Verticillium dahliae* | Li et al. 2014 |
| *Cladosporium herbarum* | Eutypa dieback of grapevine | *Eutypa lata** | Munkvold and Marois 1993 |
| *Cladosporium* sp. CEF-402 | Verticillium wilt of cotton | *Verticillium dahliae* | Li et al. 2014 |
| *Cladosporium uredinicola* | Microcyclic rust of chrysanthemum | *Puccinia horiana* | Srivastava et al. 1985 |
| *Claroideoglomus etunicatum* (*Glomus etunicatum*) | Verticillium wilt of eggplant | *Verticillium dahliae** | Matsubara et al. 1995 |
| *Clavispora fructus* (*Candida musae*) | Anthracnose of chilli | *Colletotrichum truncatum* (*Colletotrichum capsici*)*** | Chanchaichaovivat et al. 2007 |
| *Clonostachys byssicola* | Crown rot of banana | *Ceratocystis paradoxa* (*Thielaviopsis paradoxa*) | Alvindia and Natsuaki 2008 |
|  |  | *Colletotrichum musae** | Alvindia and Natsuaki 2008 |
|  |  | *Fusarium fujikuro* (*Fusarium verticillioides*)*** | Alvindia and Natsuaki 2008 |
|  |  | *Lasiodiplodia theobromae** | Alvindia and Natsuaki 2008 |
|  | Black pod rot of cacao | *Phytophthora palmivora* | Krauss and Soberanis 2001 |
| *Clonostachys rosea* | Anthracnose of chilli | *Colletotrichum truncatum* (*Colletotrichum capsici*)*** | Vasanthakumari and Shivanna 2013 |
|  | Fusarium dry rot disease of potato | *Fusarium avenaceum* and *F. caeruleum* | Jima et al. 2013 |
|  | Fusarium head blight of wheat | *Fusarium graminearum* (*Gibberella zeae*)*** | Xue et al. 2008 |
|  | Frosty pod rot of cacao | *Moniliophthora roreri* | Krauss and Soberanis 2001, Mejía et al. 2008 |
|  | Sclerotinia stem rot of oilseed rape | *Sclerotinia sclerotiorum** | Zhang et al. 2014 |
| *Clonostachys rosea* (*Gliocladium catenulatum*) | Anthracnose of blueberry | *Colletotrichum acutatum* | Verma et al. 2006 |
|  | Witches' broom disease of cocoa | *Crinipellis perniciosa* (*Moniliophthora perniciosa*)*** | Rubini et al. 2005 |
|  | Clubroot of canola | *Plasmodiophora brassicae** | Peng et al. 2001 |
|  | Damping-off and root rot of cucumber | *Pythium aphanidermatum**@ | Punja and Yip 2003 |
| *Clonostachys rosea* (*Gliocladium roseum*) | Black rot of lemon | *Alternaria citri** | Pratella and Mari 1993 |
|  | Seedling diseases of barley | *Bipolaris sorokiniana** | Knudsen et al. 1995 |
|  | Grey mould of strawberry | *Botrytis cinerea** | Pratella and Mari 1993 |
|  | Anthracnose disease in fruit (pear, apple, sour cherry and tomato) | *Colletotrichum acutatum** | Živković et al. 2010 |
|  |  | *Colletotrichum gloeosporioides** | Živković et al. 2010 |
|  | Seedling diseases of wheat | *Fusarium culmorum** | Knudsen et al. 1995 |
|  | Fusarium wilt of potato | *Fusarium oxysporum** | Pratella and Mari 1993 |
|  | Brown rot blossom blight of sweet cherry | *Monilinia fructicola* | Wittig et al. 1997 |
|  | Verticillium wilt of olive | *Verticillium dahliae* | Varo et al. 2016 |
|  | Verticillium wilt of oilseed rape | *Verticillium dahliae** | Alström 2000 |
| *Collariella bostrychodes* (*Chaetomium bostrychodes*) | Anthracnose of chilli | *Colletotrichum truncatum (Colletotrichum capsici)** | Vasanthakumari and Shivanna 2013 |
|  | White root disease of rubber | *Rigidoporus microporus** | Kaewchai and Soytong 2010 |
| *Colletotrichum gloeosporioides* | Brown blight of tea | *Colletotrichum coccodes* (*Colletotrichum camelliae*) | Rabha et al. 2014 |
|  | Black pod rot of cacao | *Phytophthora palmivora** | Mejía et al. 2008 |
|  | Grey blight of tea | *Pseudopestalotiopsis theae* (*Pestalotiopsis theae*)*** | Rabha et al. 2014 |
| *Colletotrichum gloeosporioides* (*Glomerella cingulata*) | South American leaf blight of rubber | *Microcyclus ulei** | Rocha et al. 2011 |
| *Colletotrichum magna* | Anthracnose of avocado | *Colletotrichum gloeosporioides** | Prusky et al. 1994 |
| *Coniochaeta ligniaria* | Late blight of tomato | *Phytophthora infestans** | Kim et al. 2007b |
| *Cordana* sp. KPP4-3 | Anthracnose of wild banana | *Colletotrichum musae** | Nuangmek et al. 2008 |
| *Curvularia australiensis* | Anthracnose leaf spots of guava | *Colletotrichum gloeosporioides* | Pandey et al. 1993 |
|  | Pestalotia leaf spots of guava | *Pestalotia psidii* | Pandey et al. 1993 |
| *Curvularia lunata* | Anthracnose leaf spots of guava | *Colletotrichum gloeosporioides* | Pandey et al. 1993 |
|  | Pestalotia leaf spots of guava | *Pestalotia psidii* | Pandey et al. 1993 |
| *Curvularia pallescens* | Crown rot of banana | *Ceratocystis paradoxa* (*Thielaviopsis paradoxa*)* | Alvindia and Natsuaki 2008 |
|  |  | *Colletotrichum musae** | Alvindia and Natsuaki 2008 |
|  |  | *Fusarium fujikuroi* (*Fusarium verticillioides*) | Alvindia and Natsuaki 2008 |
|  |  | *Lasiodiplodia theobromae* | Alvindia and Natsuaki 2008 |
| *Cystofilobasidium infirmominiatum* (*Cryptococcus infirmominiatus*) | Grey mould rot of pear | *Botrytis cinerea** | Chand-Goyal and Spotts 1996b, Benbow and Sugar 1999 |
|  | Side rot of pear | *Cadophora malorum* (*Phialophora malorum*)*** | Chand-Goyal and Spotts 1996b, Benbow and Sugar 1999 |
|  | Brown rot of sweet cherry | *Monilinia fructicola** | Spotts et al. 1998 |
|  | Mucor rot of pear | *Mucor piriformis** | Chand-Goyal and Spotts 1996b |
|  | Bull’s eye rot of pears | *Neofabraea malicorticis* (*Pezicula malicorticis*)* | Chand-Goyal and Spotts 1996b |
|  | Blue mould of apple | *Penicillium expansum** | Chand-Goyal and Spotts 1996a, Liu et al. 2011a |
|  | Blue mould of sweet cherry | *Penicillium expansum** | Spotts et al. 1998 |
| *Debaryomyces hansenii* | Grey mould of apple | *Botrytis cinerea** | Wisniewski et al. 1988, McLaughlin et al. 1990 |
|  | Sour rot of citrus | *Dipodascus geotrichum* (*Geotrichum citri-aurantii*)* | Chalutz and Wilson 1990 |
|  | Green mould of citrus | *Penicillium digitatum** | Wilson and Chalutz 1989, Chalutz and Wilson 1990 |
|  | Green mould of grape | *Penicillium digitatum** | Droby et al. 1989 |
|  | Blue mould of apple | *Penicillium expansum* | McLaughlin et al. 1990 |
|  | Blue mould of Citrus | *Penicillium italicum** | Wilson and Chalutz 1989, Chalutz and Wilson 1990 |
|  | Rhizopus rot of peach | *Rhizopus microsporus** | Singh 2004, Mandal et al. 2007 |
| *Debaryomyces hansenii* (*Candida famata*) | Eutypa dieback of grapevine | *Eutypa lata* | Munkvold and Marois 1993 |
|  | Green mould of citrus | *Penicillium digitatum** | Arras et al. 1998 |
| *Didymella pomorum* (*Phoma pomorum*) | Damping-off disease of Egyptian clover | *Pythium spinosum**@ | Maghazy et al. 2008 |
| *Diutina catenulata* (*Candida catenulata*) | Green mould of citrus | *Penicillium digitatum* | Perez et al. 2016 |
|  | Blue mould of citrus | *Penicillium italicum** | Perez et al. 2016 |
| *Diversispora versiformis* (*Glomus versiforme*) | Verticillium wilt of cotton | *Verticillium dahliae** | Liu 1995 |
| *Epicoccum nigrum* | Bunch rot of grape | *Botrytis cinerea** | Fowler et al. 1999, Elmer and Reglinski 2005 |
|  | Botrytis blight of tomato | *Botrytis cinerea* | Mónaco et al. 2009 |
|  | Twig blight of peach | *Monilinia laxa** | Melgarejo et al. 1986, Madrigal et al. 1994 |
| *Epicoccum nigrum* (*Epicoccum purpurascens*) | Brown rot blossom blight of sweet cherry | *Monilinia fructicola** | Wittig et al. 1997 |
|  | White mould of bean | *Sclerotinia sclerotiorum** | Boland and Inglis 1989, Zhou and Reeleder 1989 |
| *Exophiala jeanselmei* | Botrytis blight of rose | *Botrytis cinerea** | Redmond et al. 1987 |
| *Funneliformis mosseae* (*Glomus mosseae*) | Anthracnose of cucumber | *Colletotrichum orbiculare* | Saldajeno and Hyakumachi 2011 |
|  | Fusarium wilt of alfalfa | *Fusarium oxysporum* f. sp. *Medicaginis** | Hwang et al. 1992 |
|  | Root necrosis of tomato | *Phytophthora nicotianae* (*Phytophthora parasitica*)*** | Cordier et al. 1998, Vigo et al. 2000 |
|  | Damping-off in cucumber | *Rhizoctonia solani** | Saldajeno and Hyakumachi 2011 |
|  | Verticillium wilt of alfalfa | *Verticillium albo-atrum* | Hwang et al. 1992 |
|  | Verticillium wilt of cotton | *Verticillium dahliae* | Liu 1995 |
|  | Verticillium wilt of eggplant | *Verticillium dahliae** | Demir et al. 2015 |
|  | Verticillium wilt of tomato | *Verticillium dahliae* | Demir et al. 2015 |
|  | Verticillium wilt of pepper | *Verticillium dahliae* | Demir et al. 2015 |
| *Fusarium chlamydosporum* | Ground nut rust | *Puccinia arachidis* | Mathivanan and Murugesan 2000 |
| *Fusarium culmorum* | Verticillium wilt of tomato | *Verticillium albo-atrum* | Dutta et al. 1981 |
| *Fusarium equiseti* | Botrytis blight of tomato | *Botrytis cinerea* | Mónaco et al. 2009 |
|  | Anthracnose of cucumber | *Colletotrichum orbiculare** | Saldajeno and Hyakumachi 2011 |
|  | Damping-off in cucumber | *Rhizoctonia solani** | Saldajeno and Hyakumachi 2011 |
|  | Verticillium wilt of cotton | *Verticillium dahliae** | Zheng et al. 2011 |
| *Fusarium fujikuroi (Gibberella moniliformis)* | Anthracnose of chilli | *Colletotrichum truncatum* (*Colletotrichum capsici*)*** | Vasanthakumari and Shivanna 2013 |
|  | Verticillium wilt of cotton | *Verticillium dahliae* | Li et al. 2014 |
| *Fusarium graminearum* | White mould of bean | *Sclerotinia sclerotiorum** | Boland and Inglis 1989 |
| *Fusarium heterosporum* | Dollar spot of creeping bentgrass | *Clarireedia homoeocarpa* (*Sclerotinia homoeocarpa*)*** | Goodman and Burpee 1991 |
|  | White mould of bean | *Sclerotinia sclerotiorum** | Boland and Inglis 1989 |
| *Fusarium incarnatum* (*Fusarium semitectum*) | Tan spot in wheat | *Pyrenophora tritici-repentis** | Li and Sutton 1995 |
| *Fusarium lateritium* | Eutypa dieback of grapevine; | *Eutypa lata ** | Munkvold and Marois 1993 |
| *Fusarium oxysporum* | Grey mould of tomato | *Botrytis cinerea** | Zhang et al. 2014 |
|  | Anthracnose of chilli | *Colletotrichum truncatum* (*Colletotrichum capsici*)*** | Vasanthakumari and Shivanna 2013 |
|  | Leaf smut of cowpea | *Erratomyces patelii* (*Protomycopsis phaseoli*) | Adejumo et al. 1999 |
|  | Damping-off of cucumber | *Globisporangium ultimum* (*Pythium ultimum*)***@ | Benhamou et al. 2002 |
|  | Root rot of apple seedlings | *Phytophthora cactorum* | Alexander and Stewart 2001 |
|  | Late blight of tomato | *Phytophthora infestans** | Kim et al. 2007b |
|  | Root rot of pepper and phytophthora blight of pepper | *Phytophthora capsici* | Veloso and Díaz 2012 |
|  | Sclerotinia stem rot of oilseed rape | *Sclerotinia sclerotiorum** | Zhang et al. 2014 |
|  | Verticillium wilt of pepper | *Verticillium dahliae** | Veloso and Díaz 2012 |
|  | Verticillium wilt of eggplant | *Verticillium dahliae** | Malandraki et al. 2008, Gizi 2011 |
|  | Verticillium wilt of olive | *Verticillium dahliae** | Varo et al. 2016 |
|  | Verticillium wilt of cotton | *Verticillium dahliae** | Zheng et al. 2011 |
| *F. oxysporum* CAV 255 and CAV 241*#* | Fusarium wilt of banana | *Fusarium oxysporum* f. sp. *cubense** | Nel et al. 2006 |
| *F. oxysporum* C5*#* | Fusarium wilt of cucumber | *Fusarium oxysporum* f. sp. *cucumerinum** | Paulitz et al. 1987 |
| *F. oxysporum* 618-12*#* | Fusarium wilt of carnation | *Fusarium oxysporum* f. sp. *dianthi** | Postma and Rattink 1992 |
| *F. oxysporum* Fo47*#* | Fusarium wilt of tomato | *Fusarium oxysporum* f. sp. *lycopersici** | Larkin and Fravel 1998 |
| *F. oxysporum* f.sp. *dianthi* | Verticillium wilt of tomato | *Verticillium dahliae** | Matta and Garibaldi 1977 |
| *F. oxysporum* f.sp. *lycopersici* | Root rot pepper | *Phytophthora capsici* | Diaz et al. 2005 |
|  | Verticillium wilt of tomato | *Verticillium dahliae** | Matta and Garibaldi 1977 |
| *Fusarium* sp. Bx144 | Verticillium wilt of cotton | *Verticillium dahliae* | Zheng et al. 2011 |
| *Fusarium* sp. MNS1 and MNB3 | Verticillium wilt of eggplant | *Verticillium dahliae** | Narisawa et al. 2002 |
| *Fusarium tricinctum* | Sclerotinia stem rot of oilseed rape | *Sclerotinia sclerotiorum** | Zhang et al. 2014 |
| *Fusarium verticillioides* (*Fusarium moniliforme*) | Verticillium wilt of olive | *Verticillium dahliae* | Varo et al. 2016 |
| *Ganoderma lucidum* | Armillaria root rot of peach | *Armillaria mellea* and *Desarmillaria tabescens* (*Armillaria tabescens*)*** | Cox and Scherm 2006 |
| *Gibellulopsis nigrescens#* | Verticillium wilt of cotton | *Verticillium dahliae** | Zhu et al. 2013, Vagelas and Leontopoulos 2015 |
| *Gibellulopsis nigrescens#* | Verticillium wilt of mints | *Verticillium dahliae* and *Gibellulopsis nigrescens* (*Verticillium nigrescens*)*** | Melouk and Horner 1975 |
| *Gigaspora margarita* | Verticillium wilt of eggplant | *Verticillium dahliae* | Matsubara et al., 1995 |
| *Gjaerumia minor* (*Tilletiopsis minor*) | Powdery mildew of cucumber | *Podosphaera fuliginea* (*Sphaerotheca fuliginea*) | Hijwegen 1992 |
|  | Powdery mildew of rose | *Podosphaera pannosa* (*Sphaerotheca pannosa var. rosae*)*** | Verhaar et al. 1999 |
| *Gliomastix roseogrisea* (*Cephalosporium roseogriseum*) | Pestalotia leaf spots of guava | *Pestalotia psidii** | Pandey et al. 1993 |
| *Gonatobotryum parasiticum* (*Gonatorrhodiella parasitica*) | Corynespora leaf disease of rubber | *Corynespora cassiicola** | Evueh and Osemwegie 2011 |
| *Haematonectria haematococca* (*Nectria haematococca*) | Verticillium wilt of cotton | *Verticillium dahliae** | Zheng et al. 2011 |
| *Hanseniaspora opuntiae* | Grey mould of grape | *Botrytis cinerea* | Guzzon et al. 2014 |
| *Hanseniaspora uvarum* | Eutypa dieback of grape | *Eutypa lata** | Pretscher et al. 2018 |
| *Hanseniaspora uvarum* (*Kloeckera apiculata*) | Grey mould of sweet cherry | *Botrytis cinerea** | Karabulut et al. 2005 |
|  | Blue mould of sweet cherry | *Penicillium expansum* | Karabulut et al. 2005 |
|  | Blue mould of citrus | *Penicillium italicum** | Long et al. 2006 |
|  |  |  |  |
| *Hypomyces rosellus* (*Dactylium dendroides*) | Armillaria root rot of apple | *Armillaria mellea* | Raziq and Fox 2004, 2006 |
| *Induratia alba* (*Muscodor albus*) | Seedling diseases of Sugar Beet | *Aphanomyces cochlioides* | Stinson et al. 2003 |
| *Kazachstania exigua* | Blue mould of citrus | *Penicillium italicum** | Perez et al. 2016 |
| *Laetisaria arvalis* | Black scurf of potato | *Rhizoctonia solani** | Brewer and Larkin 2005 |
| *Lentinus squarrosulus* | White root disease of rubber | *Rigidoporus microporus* (*Rigidoporus lignosus*)*** | Sudirman et al. 1992 |
| *Lecanicillium psalliotae* (*Verticillium psalliotae*) | Soybean rust | *Phakopsora pachyrhizi* | Saksirirat and Hoppe 1990 |
| *Leptosphaeria biglobosa* | Sclerotinia stem rot of oilseed rape | *Sclerotinia sclerotiorum** | Zhang et al. 2014 |
| *Leptosphaeria* sp.CEF-714 | Verticillium wilt of cotton | *Verticillium dahliae** | Li et al. 2014, Yuan et al. 2017 |
| *Lipomyces tetrasporus* | Diplodia rot of guava | *Lasiodiplodia theobromae* (*Botryodiplodia theobromae*) | Mohamed and Saad 2009 |
| *Metapochonia bulbillosa* (*Verticillium bulbillosum*) | Root and butt rot disease of spruce | *Heterobasidion annosum** | Nicolotti et al. 1999 |
| *Metarhizium anisopliae* | Blackleg of beet | *Neocamarosporium betae (Phoma betae)** | Roberti et al. 1993 |
| *Metschnikowia fructicola* | Alternaria rot of grape | *Alternaria sp. ** | Karabulut et al. 2003 |
|  | Black mould of grape | *Aspergillus niger** | Karabulut et al. 2003 |
|  | Grey mould of grape | *Botrytis cinerea** | Karabulut et al. 2003 |
|  | Grey mould of sweet cherry | *Botrytis cinerea** | Karabulut et al. 2005 |
|  | Diplodia rot of guava | *Lasiodiplodia theobromae* (*Botryodiplodia theobromae*) | Mohamed and Saad 2009 |
|  | Blue mould of apple | *Penicillium expansum** | Liu et al. 2011b |
|  | Blue mould of sweet cherry | *Penicillium expansum* | Karabulut et al. 2005 |
| *Metschnikowia pulcherrima* | Grey mould of apple | *Botrytis cinerea* | Piano et al. 1997, Spadaro et al. 2002, 2004 |
|  | Post-harvest bunch rot of table grape | *Botrytis cinerea** | Parafati et al. 2015, Pretscher et al. 2018 |
|  | Anthracnose of apple | *Colletotrichum acutatum** | Conway et al. 2004 |
|  | Seedling blight and blast of rice | *Magnaporthe oryzae** | Pretscher et al. 2018 |
|  | Blue mould of apple | *Penicillium expansum** | Spadaro et al. 2002, 2004, Conway et al. 2004 |
|  | Root rot of apple and cottonwood | *Roesleria subterranea** | Pretscher et al. 2018 |
| *Metschnikowia sinensis* | Grey mould of grape | *Botrytis cinerea* | Guzzon et al. 2014 |
| *Meyerozyma caribbica* (*Pichia caribbica*) | Blue mould rot of apple | *Penicillium expansum** | Cao et al. 2013 |
|  | Patulin degradation in apples | *Penicillium expansum** | Cao et al. 2013 |
|  | Rhizopus rot of Peach | *Rhizopus stolonifer** | Xu et al. 2013 |
| *Meyerozyma guilliermondii* (*Pichia guilliermondii*) | Anthracnose of chilli | *Colletotrichum truncatum* (*Colletotrichum capsici*)*** | Chanchaichaovivat et al. 2007 |
|  | Diplodia rot of guava | *Lasiodiplodia theobromae* (*Botryodiplodia theobromae*) | Mohamed and Saad 2009 |
|  | Green mould of orange | *Penicillium digitatum* | Stirling and Stirling 1997 |
|  | Green mould of citrus | *Penicillium digitatum** | Droby et al. 1993, Arras et al. 1998 |
|  | Green mould of grape | *Penicillium digitatum** | Droby et al. 1997 |
|  | Rhizopus rot of tomato | *Rhizopus stolonifer* (*Rhizopus nigricans*)*** | Zhao et al. 2008 |
|  | Postharvest black rot of pineapple | *Thielaviopsis paradoxa* (*Ceratocystis paradoxa*)*** | Reyes et al. 2004 |
| *Microdochium bolleyi* (*Idriella bolley*) | Seedling diseases of barley | *Bipolaris sorokiniana** | Knudsen et al. 1995 |
|  | Seedling diseases of wheat | *Fusarium culmorum** | Knudsen et al. 1995 |
| *Microsphaeropsis arundinis* | White pine blister rust | *Cronartium ribicola** | Bérubé et al. 1998 |
| *Microsphaeropsis ochracea* | Sclerotinia rot of oilseed rape | *Sclerotinia sclerotiorum** | Bitsadze et al. 2015 |
|  | Verticillium wilt of oilseed rape | *Verticillium longisporum* | Stadler and von Tiedemann 2014 |
| *Microsphaeropsis* sp. F15 | Root rot of apple seedlings | *Phytophthora cactorum** | Alexander and Stewart 2001 |
| *Microsphaeropsis*sp. PMBMDF049 | South American leaf blight of rubber | *Microcyclus ulei** | Rocha et al. 2011 |
| *Minimedusa polyspora* | Fusarium basal rot of *Narcissus* | *Fusarium oxysporum* f. sp. *narcissi** | Beale and Pitt 1990 |
| *Moesziomyces aphidis* (*Pseudozyma aphidis*) | Powdery mildew of cucumber | *Podosphaera fusca* (*Podosphaera xanthii*)*** | Gafni et al. 2015 |
| *Moesziomyces rugulosus* (*Sporothrix rugulosa*) | Powdery mildew of rose | *Podosphaera pannosa* (*Sphaerotheca pannosa var. rosae*)*** | Verhaar et al. 1999 |
| *Mucor* sp. MU01 and MU02 | Verticillium wilt of olive | *Verticillium dahliae** | Varo et al. 2016 |
|  | Seedling diseases of Sugar Beet | *Globisporangium ultimum* (*Pythium ultimum*)***@ | Stinson et al. 2003 |
|  | Seedling diseases of Sugar Beet | *Rhizoctonia solani** | Stinson et al. 2003 |
|  | Common bunt of wheat | *Tilletia caries** | Goates and Mercier 2011 |
|  | Verticillium wilt of eggplant | *Verticillium dahliae** | Stinson et al. 2003 |
| *Muscodor heveae* | Brown root disease of rubber | *Pyrrhoderma noxium* (*Phellinus noxius*)*** | Siri-udom et al. 2016 |
|  | White root disease of rubber | *Rigidoporus microporus** | Siri-udom et al. 2016 |
| *Muscodor roseus* | Seedling diseases of Sugar Beet | *Aphanomyces cochlioides* | Stinson et al. 2003 |
|  | Seedling diseases of Sugar Beet | *Globisporangium ultimum* (*Pythium ultimum*)*** | Stinson et al. 2003 |
|  | Seedling diseases of Sugar Beet | *Rhizoctonia solani* | Stinson et al. 2003 |
|  | Verticillium wilt of eggplant | *Verticillium dahliae* | Stinson et al. 2003 |
| *Myrothecium* sp. PMBCDC014 & 86 | South American leaf blight of rubber | *Microcyclus ulei** | Rocha et al. 2011 |
| *Naganishia albida* (*Cryptococcus albidus*) | Postharvest decay of radish | *Alternaria* spp. | Chen et al. 2012 |
|  | Grey mould of apple | *Botrytis cinerea** | Roberts 1991, Fan and Tian 2001, Tian et al. 2002a |
|  | Grey mould of pear | *Botrytis cinerea** | Fan and Tian 2001 |
|  | Postharvest decay of radish | *Fusarium* spp.* | Chen et al. 2012 |
|  | Mucor rot of pear | *Mucor piriformis** | Lopatecki and Peters 1972, Roberts 1990b |
|  | Blue mould of apple | *Penicillium expansum** | Fan and Tian 2001, Tian et al. 2002a |
|  | Blue mould of pear | *Penicillium expansum* | Tian et al. 2002a |
| *Nakazawaea ernobii* (*Candida ernobii*) | Stem-end rot in citrus | *Lasiodiplodia theobromae* (*Diplodia natalensis*)*** | Liu et al. 2010b |
| *Neocamarosporium betae* (*Phoma betae*) | Fusarium ear blight of wheat | *Fusarium culmoru* & *Microdochium nivale** | Diamond and Cooke 2003 |
| *Neocosmospora solani* (*Fusarium solani*) | Anthracnose of chilli | *Colletotrichum truncatum* (*Colletotrichum capsici*)*** | Vasanthakumari and Shivanna 2013 |
|  | Fusarium wilt of tomato | *Fusarium oxysporum* f. sp. *lycopersici** | Larkin and Fravel 1998 |
|  | Verticillium wilt of cotton | *Verticillium dahliae** | Li et al. 2014 |
|  | Verticillium wilt of cotton | *Verticillium dahliae** | Zheng et al. 2011 |
| *Nigrospora oryzae* | Verticillium wilt of cotton | *Verticillium dahliae* | Li et al. 2014 |
| *Nodulisporium* sp. JWL1-8 | Anthracnose of wild banana | *Colletotrichum musae** | Nuangmek et al. 2008 |
| *Ogataea methanolica* (*Pichia methanolica*) | Seedling blight and blast of rice | *Magnaporthe oryzae** | Pretscher et al. 2018 |
| *Oidiodendron* sp. F762 | Root rot of apple seedlings | *Phytophthora cactorum** | Alexander and Stewart 2001 |
| *Paecilomyces* sp. (F46) | Root rot of apple seedlings | *Phytophthora cactorum** | Alexander and Stewart 2001 |
| *Paecilomyces variotii* | Black rot of lemon | *Alternaria citri** | Pratella and Mari 1993 |
|  | Grey mould of kiwifruit and strawberry | *Botrytis cinerea* | Pratella and Mari 1993 |
|  | Fusarium wilt of potato | *Fusarium oxysporum* | Pratella and Mari 1993 |
| *Papiliotrema flavescens* (*Cryptococcus nodaensis*) | Fusarium head blight on durum wheat | *Fusarium graminearum* (*Gibberella zeae*)*** | Schisler et al. 2002 |
| *Papiliotrema laurentii* (*Cryptococcus laurentii*) | Black mould of jujube | *Alternaria alternata* | Qin and Tian 2004, Tian et al. 2005 |
|  | Black mould of sweet cherry | *Alternaria alternata* | Qin et al. 2004 |
|  | Grey mould of apple | *Botrytis cinerea** | Roberts 1990a |
|  | Grey mould of peach | *Botrytis cinerea** | Zhang et al. 2007a |
|  | Grey mould of pear | *Botrytis cinerea** | Chand-Goyal and Spotts 1996b, Zhang et al. 2005a |
|  | Grey mould of tomato | *Botrytis cinerea** | Xi and Tian 2005 |
|  | Grey mould rot of sweet cherry | *Botrytis cinerea** | Qin et al. 2004 |
|  | Side rot in pear | *Cadophora malorum* (*Phialophora malorum*)*** | Chand-Goyal and Spotts 1996b |
|  | Sour rot of Citrus | *Dipodascus geotrichum* (*Geotrichum citri-aurantii*)* | Liu et al. 2010a |
|  | Brown rot of sweet cherry | *Monilinia fructicola** | Chand-Goyal and Spotts 1996a, Qin et al. 2006 |
|  | Brown rot of jujube | *Monilinia fructicola* | Qin and Tian 2004 |
|  | Brown rot of peach | *Monilinia fructicola** | Yao and Tian 2005 |
|  | Mucor rot of pear | *Mucor piriformis** | Roberts 1990b, Lopatecki and Peters 1972 |
|  | Bull’s eye rot of pears | *Neofabraea malicorticis* (*Pezicula malicorticis*)*** | Chand-Goyal and Spotts 1996b |
|  | Postharvest decay of arbutus berries | *Penicillium citrinum* | Zheng et al. 2004 |
|  | Blue mould of sweet cherry | *Penicillium expansum** | Qin et al. 2004 |
|  | Blue mould of apple | *Penicillium expansum** | Chand-Goyal and Spotts 1996a |
|  | Blue mould of pear | *Penicillium expansum** | Chand-Goyal and Spotts 1996b |
|  | Blue mould of peach | *Penicillium expansum** | Zhang et al. 2007a, Yao and Tian 2005 |
|  | Blue mould of jujube | *Penicillium expansum** | Cao et al. 2012 |
|  | Blue mould of oranges | *Penicillium italicum** | Zhang et al. 2005b |
|  | Damping-off disease of tomato | *Pythium aphanidermatum*@ | Xi and Tian 2005 |
|  | Rhizopus rot of strawberry | *Rhizopus stolonifer** | Zhang et al. 2007b |
|  | Rhizopus decay of peach | *Rhizopus stolonifer** | Zhang et al. 2007a |
|  | Rhizopus rot of sweet cherry | *Rhizopus stolonifer** | Qin et al. 2004 |
| *Paraboeremia putaminum* (*Phoma putaminum*) | Anthracnose of chilli | *Colletotrichum truncatum* (*Colletotrichum capsici*)*** | Vasanthakumari and Shivanna 2013 |
| *Paramyrothecium roridum* (*Myrothecium roridum*) | Anthracnose leaf spots of guava | *Colletotrichum gloeosporioides** | Pandey et al. 1993 |
|  | Pestalotia leaf spots of guava | *Pestalotia psidii** | Pandey et al. 1993 |
|  | Root rot of avocado | *Phytophthora cinnamomi** | Gees and Coffey 1989 |
|  | Verticillium wilt of cotton | *Verticillium dahliae* | Zheng et al. 2011 |
| *Paraphaeosphaeria minitans* (*Coniothyrium minitans*) | Verticillium wilt of olive | *Verticillium dahliae** | Varo et al. 2016 |
|  | Sclerotinia lettuce drop | *Sclerotinia minor** | Rabeendran et al. 2006 |
|  | Sclerotinia wilt of sunflower | *Sclerotinia sclerotiorum** | McLaren et al. 1994 |
|  | Sclerotinia disease of lettuce | *Sclerotinia sclerotiorum* | Budge and Whipps 1991, Jones and Whipps 2002 |
|  | Sclerotinia rot of oilseed rape | *Sclerotinia sclerotiorum** | Bitsadze et al. 2015 |
|  | White mould of dry bean | *Sclerotinia sclerotiorum** | Huang et al. 2000 |
| *Parasarocladium breve* (*Acremonium breve*) | Grey mould of apple | *Botrytis cinerea** | Janisiewicz 1988 |
| *Penicillium bilaiae* | Black-rot disease of onion | *Aspergillus niger** | Khokhar et al. 2013 |
| *Penicillium copticola* | Grey mould of cannabis | *Botrytis cinerea** | Kusari et al. 2013 |
|  | Pink rot disease of cannabis | *Trichothecium roseum** | Kusari et al. 2013 |
| *Penicillium chrysogenum* | Anthracnose of chilli | *Colletotrichum truncatum* (*Colletotrichum capsici*)*** | Vasanthakumari and Shivanna 2013 |
|  | Fusarium wilt of cotton | *Fusarium oxysporum* f. sp *vasinfectum* | Dong et al. 2006 |
|  | Verticillium wilt of tomato | *Verticillium albo-atrum** | Dutta et al. 1981 |
|  | Verticillium wilt of cotton | *Verticillium dahliae** | Dong et al. 2003, 2006 |
| *Penicillium citreonigrum* (*Penicillium citreoviride*) | Leaf spot of toothache plant (*Spilanthes oleracea*) | *Alternaria alternata** | Thakur and Harsh 2014 |
| *Penicillium citrinum* | Anthracnose of chilli | *Colletotrichum truncatum* (*Colletotrichum capsici*)*** | Vasanthakumari and Shivanna 2013 |
|  | Basal stem rot oil palm | *Ganoderma orbiforme (Ganoderma boninense)** | Dharmaputra et al. 1989 |
| *Penicillium expansum* | White rot of onion | *Stromatinia cepivora* (*Sclerotium cepivorum*)*** | Harrison and Stewart 1988 |
| *Penicillium frequentans* | Wilting, yellowing or necrosis off peach | *Monilinia laxa** | Melgarejo et al. 1986 |
|  | Brown rot of stone fruit (apricot) | *Monilinia laxa** | De Cal et al. 2002 |
|  | Brown rot and twig blight of peach | *Monilinia laxa** | De Cal et al. 1990, Guijarro et al. 2006 |
|  | Brown rot of peach | *Monilinia laxa**; *M. fructigena**; *M. fructicola** | Guijarro et al. 2007 |
| *Penicillium herquei* | Leaf spot of toothache plant (Spilanthes oleracea) | *Alternaria alternata** | Thakur and Harsh 2014 |
| *Penicillium olsonii* | Black-rot disease of onion | *Aspergillus niger** | Khokhar et al. 2013 |
| *Penicillium oxalicum* | Crown rot of banana | *Ceratocystis paradoxa* (*Thielaviopsis paradoxa*) | Alvindia and Natsuaki 2008 |
|  | Crown rot of banana | *Colletotrichum musae** | Alvindia and Natsuaki 2008 |
|  | Crown rot of banana | *Fusarium fujikuroi* (*Fusarium verticillioides*) | Alvindia and Natsuaki 2008 |
|  | Fusarium wilt of tomato | *Fusarium oxysporum* f. sp. *lycopersici** | Larena et al. 2003, Sabuquillo et al. 2005 |
|  | Crown rot of banana | *Lasiodiplodia theobromae* | Alvindia and Natsuaki 2008 |
|  | Pestalotia leaf spots of guava | *Pestalotia psidii** | Pandey et al. 1993 |
|  | Verticillium wilt of tomato | *Verticillium dahliae** | Larena et al. 2003 |
| *Penicillium roqueforti* | Black-rot disease of onion | *Aspergillus niger** | Khokhar et al. 2013 |
| *Penicillium simplicissimum* | Verticillium wilt of cotton | *Verticillium dahliae** | Li et al. 2014, Yuan et al. 2017 |
| *Penicillium* sp. F120 | Root rot of apple seedlings | *Phytophthora cactorum** | Alexander and Stewart 2001 |
| *Penicillium* sp. CEF-718 | Verticillium wilt of cotton | *Verticillium dahliae** | Li et al. 2014 |
| *Penicillium* sp. MNT8 | Verticillium wilt of eggplant | *Verticillium dahliae** | Narisawa et al. 2002 |
| *Penicillium spinulosum* (*Penicillium nigricans*) | Damping-off disease of Egyptian clover | *Pythium spinosum**@ | Maghazy et al. 2008 |
| *Penicillium striatisporum* | Phytophthora root rot of chilli pepper | *Phytophthora capsici** | Ma et al. 2008 |
| *Penicillium sublateritium* | Leaf spot disease of toothache plant (Spilanthes oleracea) | *Alternaria alternata** | Thakur and Harsh 2014 |
| *Penicillium sumatrense* | Witches' broom disease of cocoa | *Botrytis cinerea** | Rubini et al. 2005 |
|  | Clubroot of canola | *Plasmodiophora brassicae** | Peng et al. 2001 |
| *Penicillium viridicatum* | Black-rot disease of onion | *Aspergillus niger** | Khokhar et al. 2013 |
| *Pestalotiopsis neglecta* | Anthracnose of avocado | *Colletotrichum gloeosporioides** | Adikaram and Karunaratne 1998 |
|  | Stem-end rot of avocado | *Phoma* spp.*** | Adikaram and Karunaratne 1998 |
| *Pestalotiopsis* sp. PMBMDF087 | South American leaf blight of rubber | *Microcyclus ulei** | Rocha et al. 2011 |
| *Phaeotheca dimorphospora* | Shoot blight & Scleroderris canker of pine | *Gremmeniella abietina** | Yang et al. 1993, 1995 |
|  | Scleroderris canker of larch | *Gremmeniella laricina** | Yang et al. 1993 |
|  | Root and butt rot disease of conifers | *Heterobasidion annosum** | Roy et al. 2001, 2003 |
|  | Dutch elm disease | *Ophiostoma ulmi ** | Yang et al. 1993 |
|  | Septoria leaf spot, canker of poplar | *Sphaerulina musiva* (*Septoria musiva*)*** | Yang et al. 1993, 1994 |
| *Phialocephala fortini* | Verticillium wilt of eggplant | *Verticillium dahliae** | Nicolotti et al. 1999, Narisawa et al. 2002 |
|  | Verticillium yellows of Chinese cabbage | *Verticillium dahliae* | Narisawa et al. 2004 |
| *Phlebiopsis gigantea* (*Phlebia gigantea*) | Butt and stem rot of pine trees | *Heterobasidion annosum* | Stirling and Stirling 1997 |
| *Phlebiopsis gigantea* | Root and butt rot disease of conifers | *Heterobasidion annosum** | Roy et al. 2003 |
| *Phoma* sp. isolate 177 | Anthracnose of chilli | *Colletotrichum truncatum* (*Colletotrichum capsici*)*** | Vasanthakumari and Shivanna 2013 |
| *Phoma sp.* PH01& 02 | Verticillium wilt of olive | *Verticillium dahliae* | Varo et al. 2016 |
| *Phomopsis* sp. By231 | Verticillium wilt of cotton | *Verticillium dahliae** | Zheng et al. 2011 |
| *Phomopsis* sp. By254 | Verticillium wilt of cotton | *Verticillium dahliae* | Zheng et al. 2011 |
| *Pichia fermentans* | Green mould of Citrus | *Penicillium digitatum** | Perez et al. 2016 |
|  | Blue mould of Citrus | *Penicillium italicum** | Perez et al. 2016 |
| *Pichia kluyveri* | Seedling blight and blast of rice | *Magnaporthe oryzae** | Pretscher et al. 2018 |
| *Pichia kudriavzevii* (*Issatchenkia orientalis*) | Anthracnose of chilli | *Colletotrichum truncatum* (*Colletotrichum capsici*)*** | Chanchaichaovivat et al. 2007 |
| *Pichia membranifaciens* | Black mould of sweet cherry | *Alternaria alternata** | Qin et al. 2004 |
|  | Grey mould rot of sweet cherry | *Botrytis cinerea* | Qin et al. 2004 |
|  | Brown rot of sweet cherry | *Monilinia fructicola* | Qin et al. 2006 |
|  | Blue mould rot of sweet cherry | *Penicillium expansum** | Qin et al. 2004 |
|  | Rhizopus rot of sweet cherry | *Rhizopus stolonifer** | Qin et al. 2004 |
|  | Rhizopus rot of nectarine | *Rhizopus stolonifer** | Qing and Shiping 2000 |
| *Pichia terricola* (*Issatchenkia terricola*) | Grey mould of grape | *Botrytis cinerea* | Guzzon et al. 2014 |
| *Plectosphaerella cucumerina* (*Plectosporium tabacinum*) | Crown rot of banana | *Ceratocystis paradoxa* (*Thielaviopsis paradoxa*) | Alvindia and Natsuaki 2008 |
|  | Crown rot of banana | *Colletotrichum musae* | Alvindia and Natsuaki 2008 |
|  | Crown rot of banana | *Lasiodiplodia theobromae* | Alvindia and Natsuaki 2008 |
| *Plectosphaerella* sp. CEF-646 | Verticillium wilt of cotton | *Verticillium dahliae* | Li et al. 2014 |
| *Purpureocillium lilacinum* (*Paecilomyces lilacinus*) | Charcoal rot of sunflower and mung bean | *Macrophomina phaseolina** | Hussain et al. 1990 |
|  | Tan spot of wheat | *Pyrenophora tritici-repentis** | Larran et al. 2016 |
|  | Damping-off disease of Egyptian clover | *Pythium spinosum**@ | Maghazy et al. 2008 |
|  | Verticillium wilt of eggplant | *Verticillium dahliae** | Marois et al. 1982 |
| *Rhizoctonia solani* | Verticillium wilt of cotton | *Verticillium dahliae* | Li et al. 2014 |
| *Rhizophagus fasciculatus* (*Glomus fasciculatum*) | Fusarium wilt of alfalfa | *Fusarium oxysporum* f. sp. *medicaginis* | Hwang et al., 1992 |
|  | Verticillium wilt of alfalfa | *Verticillium albo-atrum** | Hwang et al., 1992 |
| *Rhizophagus intraradices* (*Glomus intraradices*) | Fusarium crown and root rot of tomato | *Fusarium oxysporum* f. sp. *radicis-lycopersici** | Datnoff et al. 1995 |
|  | Verticillium wilt of olive | *Verticillium dahliae** | Varo et al. 2016 |
| *Rhizophagus clarus* (*Glomus clarum*) | Southern stem rot of Jerusalem artichoke | *Athelia rolfsii* (*Sclerotium rolfsii*)*** | Sennoi et al. 2013 |
| *Rhizopus stolonifer* | Barley powdery mildew | *Blumeria graminis* f. sp. *hordei** | Haugaard et al. 2001 |
| *Rhodotorula glutinis* | Black mould of sweet cherry | *Alternaria alternata** | Qin et al. 2004 |
|  | Alternaria rot of jujube/ Black mould of jujube | *Alternaria alternata* | Tian et al. 2005 |
|  | Grey mould of apple | *Botrytis cinerea** | Zhang et al. 2009 |
|  | Grey mould of sweet cherry | *Botrytis cinerea** | Qin et al. 2004 |
|  | Grey mould of pear | *Botrytis cinerea** | Chand-Goyal and Spotts 1996b, Benbow and Sugar 1999 |
|  | Side rot of pear | *Cadophora malorum* (*Phialophora malorum*)*** | Chand-Goyal and Spotts 1996b, Benbow and Sugar 1999 |
|  | Mucor rot of pear | *Mucor piriformis* | Chand-Goyal and Spotts 1996b |
|  | Bull’s eye rot of pears | *Neofabraea malicorticis* (*Pezicula malicorticis*)*** | Chand-Goyal and Spotts 1996b |
|  | Blue mould of jujube | *Penicillium expansum* | Tian et al. 2005 |
|  | Blue mould of apple | *Penicillium expansum** | Chand-Goyal and Spotts 1996a, Zhang et al. 2009 |
|  | Blue mould of pear | *Penicillium expansum** | Chand-Goyal and Spotts 1996b |
|  | Blue mould of sweet cherry | *Penicillium expansum** | Qin et al. 2004 |
|  | Rhizopus rot of sweet cherry | *Rhizopus stolonifer** | Qin et al. 2004 |
| *Rhodotorula mucilaginosa* (*Rhodotorula rubra*) | Eutypa dieback of grapevine | *Eutypa lata* | Munkvold and Marois 1993 |
| *Rhodotorula paludigena* (*Rhodosporidium paludigenum*) | Black mould of Chinese winter jujubes | *Alternaria alternata* | Wang et al. 2010 |
|  | Black rot of cherry tomato | *Alternaria alternata** | Wang et al. 2008 |
|  | Sour rot of Citrus | *Dipodascus geotrichum* (*Geotrichum citri-aurantii*) | Liu et al. 2010a |
|  | Blue mould of pear | *Penicillium expansum** | Wang et al. 2010 |
| *Robbauera albescens* (*Tilletiopsis albescens*) | Powdery mildew of cucumber | *Podosphaera fuliginea* (*Sphaerotheca fuliginea*)*** | Knudsen and Skou 1993 |
| *Robillarda sessilis* | Anthracnose of chilli | *Colletotrichum truncatum*(*Colletotrichum capsici*)*** | Vasanthakumari and Shivanna 2013 |
| *Saccharomyces bayanus* | Brown rot of peach | *Monilinia fructicola* | Zhou et al. 2008 |
| *Saccharomyces bayanus* (*Saccharomyces uvarum*) | Brown rot of peach | *Monilinia fructicola* | Zhou et al. 2008 |
| *Saccharomyces cerevisiae* | Blue mould of Citrus | *Penicillium italicum** | Perez et al. 2016 |
|  | Crown rot of banana | *Colletotrichum musae** | Zhimo et al. 2016 |
|  | Post-harvest bunch rot of table grape | *Botrytis cinerea** | Parafati et al. 2015 |
|  | Brown rot of peach | *Monilinia fructicola** | Zhou et al. 2008 |
| *Saitozyma flava* (*Cryptococcus flavus*) | Grey mould of apple | *Botrytis cinerea** | Kheireddine et al. 2018 |
| *Saitozyma flava* (*Cryptococcus flavus*) | Grey mould of apple | *Botrytis cinerea** | Roberts 1991 |
|  | Mucor rot of pear | *Mucor piriformis** | Lopatecki and Peters 1972, Roberts 1990b |
| *Sarocladium implicatum* (*Acremonium implicatum*) | Ascochyta blight of chickpea | *Ascochyta rabiei** | Rajakumar et al. 2005 |
|  | Verticillium wilt of cotton | *Verticillium dahliae* | Li et al. 2014 |
|  | Crown rot of banana | *Lasiodiplodia theobromae* | Alvindia and Natsuaki 2008 |
|  |  | *Colletotrichum musae* | Alvindia and Natsuaki 2008 |
| *Schizophyllum commune* | Armillaria root rot of peach | *Armillaria mellea* and Desarmillaria tabescens* (*Armillaria tabescens*) *** | Cox and Scherm 2006 |
| *Septoglomus deserticola* (*Glomus deserticola*) | Verticillium wilt of pepper | *Verticillium dahliae* | Garmendia et al. 2004 |
| *Serendipita indica* (*Piriformospora indica*) | Verticillium wilt of tomato | *Verticillium dahliae* | Fakhro et al. 2010 |
| *Simiglomus hoi* (*Glomus hoi*) | Verticillium wilt of cotton | *Verticillium dahliae* | Liu 1995 |
| *Simplicillium lamellicola* | Sclerotinia stem rot of oilseed rape | *Sclerotinia sclerotiorum** | Zhang et al. 2014 |
| *Simplicillium lanosoniveum* | Soybean rust | *Phakopsora pachyrhizi* | Ward et al. 2012, Gauthier et al. 2014 |
| *Sordaria fimicola* | Anthracnose of chilli | *Colletotrichum truncatum* (*Colletotrichum capsici*)*** | Vasanthakumari and Shivanna 2013 |
| *Stachybotrys chartarum* | Verticillium wilt of cotton | *Verticillium dahliae* | Li et al. 2014 |
| *Stemphylium solani* | Verticillium wilt of cotton | *Verticillium dahliae* | Li et al. 2014 |
| *Stilbella aciculosa* | Stem canker of potato | *Rhizoctonia solani* | Brewer and Larkin 2005 |
| *Talaromyces aculeatus* (*Penicillium aculeatum*) | Verticillium wilt of cotton | *Verticillium dahliae* | Li et al. 2014 |
| *Talaromyces flavus* | Stem rot of bean | *Athelia rolfsii* (*Sclerotium rolfsii*)*** | Madi et al. 1997 |
|  | Sclerotinia wilt of sunflower | *Sclerotinia sclerotiorum** | McLaren et al. 1994 |
|  | White mould of dry bean | *Sclerotinia sclerotiorum* | Huang et al. 2000 |
|  | Verticillium wilt of hops | *Verticillium albo-atrum** | Solarska et al. 2000 |
|  | Verticillium wilt of potato | *Verticillium albo-atrum** | Naraghi et al. 2010a |
|  | Verticillium wilt of cucumber | *Verticillium albo-atrum** | Naraghi et al. 2010b |
|  | Verticillium wilt of eggplant | *Verticillium dahliae** | Marois et al. 1982, Nagtzaam et al. 1998 |
|  | Verticillium wilt of cotton | *Verticillium dahliae** | Naraghi et al. 2006, Li et al. 2014 |
|  | Verticillium wilt of potato | *Verticillium dahliae** | Nagtzaam et al. 1998 |
| *Talaromyces flavus* (*Penicillium vermiculatum*) | Verticillium wilt of tomato | *Verticillium albo-atrum** | Dutta et al. 1981 |
| *Talaromyces funiculosus* (*Penicillium funiculosum*) | Fusarium crown and root rot of tomato | *Fusarium oxysporum* f. sp. *radicis-lycopersici* | Marois et al. 1981 |
|  | Phytophthora root rot of azalea | *Phytophthora cinnamomi** | Fang and Tsao 1995 |
|  | Phytophthora root rot of sweet orange | *Phytophthora nicotianae* (*Phytophthora parasitica*)*** | Fang and Tsao 1995 |
|  | Damping-off disease of Egyptian clover | *Pythium spinosum**@ | Maghazy et al. 2008 |
|  | Verticillium wilt of cotton | *Verticillium dahliae* | Li et al. 2014 |
| *Talaromyces funiculosus* (*Penicillium funiculosum*)*#* | Fruitlet core rot of pineapple/ leathery pocket/black Spots of Pineapple | *Talaromyces funiculosus* (*Penicillium funiculosum*)*** | Lim and Rohrbach 1980, Wilson et al. 1991 |
| *Talaromyces islandicus* (*Penicillium islandicum*) | Damping-off disease of Egyptian clover | *Pythium spinosum**@ | Maghazy et al. 2008 |
| *Talaromyces pinophilus* (*Penicillium pinophilum*) | Verticillium wilt of cotton | *Verticillium dahliae* | Li et al. 2014 |
| *Talaromyces purpureogenus* (*Penicillium purpurogenum*) | Wilting, yellowing or necrosis off peach | *Monilinia laxa** | Melgarejo et al. 1986 |
| *Talaromyces ruber* (*Penicillium rubrum*) | Anthracnose of chilli | *Colletotrichum truncatum* (*Colletotrichum capsici*)*** | Vasanthakumari and Shivanna 2013 |
| *Talaromyces stollii* | Verticillium wilt of cotton | *Verticillium dahliae** | Li et al. 2014 |
| *Tausonia pullulans* (*Trichosporon pullulans*) | Black mould of sweet cherry | *Alternaria alternata** | Qin et al. 2004 |
|  | Grey mould of sweet cherry | *Botrytis cinerea** | Qin et al. 2004 |
|  | Stem end rot disease in kiwifruit | *Botrytis cinerea** | Cook et al. 1999 |
|  | Blue mould of sweet cherry | *Penicillium expansum** | Qin et al. 2004 |
|  | Rhizopus rot of sweet cherry | *Rhizopus stolonifer** | Qin et al. 2004 |
| *Teratosperma sclerotivorum* (*Sporidesmium sclerotivorum*) | Sclerotinia lettuce drop | *Sclerotinia minor** | Adams and Ayers 1982, Adams and Fravel 1990 |
| *Tilletiopsis pallescens* | Powdery mildew of cucumber | *Podosphaera fuliginea* (*Sphaerotheca fuliginea*)*** | Urquhart et al. 1994 |
| *Tilletiopsis washingtonensis* | Powdery mildew of cucumber | *Podosphaera fuliginea* (*Sphaerotheca fuliginea*)*** | Urquhart et al. 1994 |
| *Tolypocladium* sp. ALF 902 | Black-pod rot of cacao | *Phytophthora palmivora* | Hanada et al. 2010 |
| *Torulaspora delbrueckii* | Grey mould of apple | *Botrytis cinerea** | Pretscher et al. 2018 |
|  | Seedling blight and blast of rice | *Magnaporthe oryzae** | Pretscher et al. 2018 |
| *Torulaspora delbrueckii* (*Saccharomyces delbrueckii*) | Brown rot of peach | *Monilinia fructicola** | Zhou et al. 2008 |
| *Torulaspora globosa* | Anthracnose in sorghum | *Colletotrichum sublineolum* | Rosa et al. 2010 |
| *Trametes sanguinea* (*Polyporus sanguineus*) | Forest tree root rot | *Ganoderma lucidum** | Shukla and Anil 1996 |
| *Trichoderma asperellum* | Anthracnose of mango | *Colletotrichum gloeosporioides** | de los Santos-Villalobos et al. 2013 |
|  | Fusarium wilt of tomato | *Fusarium oxysporum* f.sp. *lycopersici** | Cotxarrera et al. 2002, El-Komy et al. 2015 |
|  | Fusarium wilt of potato | *Fusarium oxysporum* f. sp. *tuberosi** | Ommati et al. 2013 |
|  | Root rot of pepper | *Phytophthora capsici** | Segarra et al. 2013 |
|  | Seed-borne disease of radish | *Rhizoctonia solani** | Lee 2018 |
|  | Black rot of pineapple | *Thielaviopsis paradoxa** | Wijesinghe et al. 2010 |
|  | Verticillium wilt of bell pepper | *Verticillium dahliae** | Ślusarski and Pietr 2009 |
|  | Verticillium wilt of olive | *Verticillium dahliae** | Carrero-Carrón et al. 2016 |
| *Trichoderma atroviride* | Ring rot of apple | *Botryosphaeria berengeriana* f. sp. *pyricola** | Kexiang et al. 2002 |
|  | Grey mould of strawberry | *Botrytis cinerea** | Freeman et al. 2004, Card et al. 2009 |
|  | Anthracnose of strawberry | *Colletotrichum acutatum** | Freeman et al. 2004 |
|  | Grapevine trunk diseases | *Diplodia seriata** | Kotze et al. 2011 |
|  | Collar canker disease of tea | *Diaporthe theae* (*Phomopsis theae*)*** | Anita and Ponmurugan 2011 |
|  | Grapevine trunk diseases | *Eutypa lata** | Kotze et al. 2011 |
|  | Fusarium wilt of potato | *Fusarium oxysporum* f. sp*. tuberosi* | Ommati et al. 2013 |
|  | Grapevine trunk diseases | *Lasiodiplodia theobromae** | Kotze et al. 2011 |
|  | Grapevine trunk diseases | *Neofusicoccum australe** | Kotze et al. 2011 |
|  | Grapevine trunk diseases | *Neofusicoccum parvum** | Kotze et al. 2011 |
|  | Grapevine trunk diseases | *Phaeomoniella chlamydospora** | Kotze et al. 2011 |
|  | Fruit rot of brinjal | *Phomopsis vexans** | Das et al. 2014 |
|  | Bird’s eye spot disease of tea | *Pseudocercospora ocellata* (*Cercospora theae*) | Gnanamangai and Ponmurugan 2012 |
|  | Sclerotinia stem rot of rapesee | *Sclerotinia sclerotiorum** | Liu and Wen 2005 |
|  | Shoot blight of pine | *Diplodia sapinea* (*Diplodia pinea*)*** | Santamaría et al. 2012 |
|  | Grapevine trunk diseases | *Diplodia sapinea* (*Diplodia pinea*)*** | Kotze et al. 2011 |
|  | Diplodia dieback of pine | *Diplodia sapinea* (*Diplodia pinea*) | Regliński et al. 2012 |
|  | Seed-borne disease of radish | *Rhizoctonia solani** | Lee 2018 |
|  | Verticillium wilt of cotton | *Verticillium dahliae* | Li et al. 2014 |
| *Trichoderma aureoviride* | Seedling blight of wheat | *Fusarium graminearum** | Dal Bello et al. 2002 |
|  | Tan spot of wheat | *Pyrenophora tritici-repentis* | Perello et al. 2003 |
| *Trichoderma brevicompactum* | Anthracnose of mango | *Colletotrichum gloeosporioides** | de los Santos-Villalobos et al. 2013 |
|  | Fusarium wilt of potato | *Fusarium oxysporum* f. sp. *tuberosi* | Ommati et al. 2013 |
| *Trichoderma deliquescens* (*Gliocladium viride*) | Basal stem rot disease of oil palm | *Ganoderma orbiforme* (*Ganoderma boninense*)*** | Susanto et al. 2005 |
| *Trichoderma gamsii* | Root-rot of notoginseng | *Epicocum nigrum**, *Fusarium flocciferu**, *Phoma herbarum**, *Scytalidium lignicola** | Chen et al. 2016 |
| *Trichoderma ghanense* (*Trichoderma parceramosum*) | Chestnut blight | *Cryphonectria parasitica** | Arisan-Atac et al. 1995 |
| *Trichoderma hamatum* | Early blight of tomato | *Alternaria solani** | El-Rafai et al. 2003 |
|  | Anthracnose of bean | *Colletotrichum lindemuthianum** | Padder and Sharma 2011 |
|  | Pink canker of apple stems and twigs | *Erythricium salmonicolor* (*Corticium salmonicolor*)*** | Durga et al. 2014 |
|  | Seedling blight of wheat | *Fusarium graminearum** | Dal Bello et al. 2002 |
|  | Fusarium wilt of tomato | *Fusarium oxysporum* f. sp. *lycopersici** | Larkin and Fravel 1998, El-Rafai et al. 2003 |
|  | Red root disease of rubber | *Ganoderma philippii* (*Ganoderma pseudoferreum*)*** | Ogbebor et al. 2010 |
|  | Tan spot of wheat | *Pyrenophora tritici-repentis** | Larran et al. 2016 |
|  | White root disease of rubber | *Rigidoporus microporus** | Kaewchai and Soytong 2010 |
|  | Sclerotinia lettuce drop | *Sclerotinia minor** | Rabeendran et al. 2006 |
|  | Sclerotinia rot of lettuce | *Sclerotinia sclerotiorum** | Gracia-Garza et al. 1997 |
|  | Sclerotinia stem rot of rapeseed | *Sclerotinia sclerotiorum** | Liu and Wen 2005 |
|  | Verticillium wilt of tomato | *Verticillium dahliae** | El-Rafai et al. 2003 |
| *Trichoderma harzianum* | Collapse of muskmelon | *Acremonium cucurbitacearum* | Grondona et al. 1997 |
|  | Black fruit spot disease of persimmon | *Alternaria alternata** | Batta 2001 |
|  | Leaf spot of toothache plant (*Spilanthes oleracea*) | *Alternaria alternata** | Thakur and Harsh 2014 |
|  | Brown spot on tobacco | *Alternaria alternata** | Gveroska and Ziberoski 2012 |
|  | Black rot of lemon | *Alternaria citri** | Pratella and Mari 1993 |
|  | Damping-off disease of sugar beet | *Aphanomyces cochlioides** | Grondona et al. 1997 |
|  | Armillaria root rot of apple | *Armillaria mellea** | Raziq and Fox 2006 |
|  | Collar rot of groundnut | *Aspergillus niger** | Devi and Prasad 2009, Kumari and Singh 2017 |
|  | Neck rot of onion | *Aspergillus* sp.* | Lee et al. 2001 |
|  | Sclerotium root rot of sugarbeet | *Athelia rolfsii* (*Sclerotium rolfsii*)*** | Upadhyay and Mukhopadhyay 1986 |
|  | Collar and stem rot of tomato | *Athelia rolfsii* (*Sclerotium rolfsii*)*** | Suriyagamon et al. 2018 |
|  | Damping-off disease and blight of snapbean | *Athelia rolfsii* (*Sclerotium rolfsii*)*** | Papavizas and Lewis 1989 |
|  | Damping-off disease of bean | *Athelia rolfsii* (*Sclerotium rolfsii*)*** | Elad et al. 1980 |
|  | Brown spot of rice | *Bipolaris oryzae** | Harish et al. 2008 |
|  | Ring rot of apple | *Botryosphaeria berengeriana* f.sp. *pyricola** | Kexiang et al. 2002 |
|  | Neck rot of onion | *Botrytis allii** | Lee et al. 2001 |
|  | Grey mould of pear | *Botrytis cinerea* | Batta 2007 |
|  | Grey mould of strawberry | *Botrytis cinerea** | Freeman et al. 2004, Batta 2007 |
|  | Botrytis blight of tomato | *Botrytis cinerea* | Mónaco et al. 2009 |
|  | Grey mould of cucumber | *Botrytis cinerea** | Elad 2000 |
|  | Grey mould of Kiwifruit | *Botrytis cinerea** | Batta 2007 |
|  | Grey mould of strawberry | *Botrytis cinerea** | Pratella and Mari 1993 |
|  | Grey mould of grape | *Botrytis cinerea** | Dubos 1984, Elad 1994, Batta 2007 |
|  | Pineapple disease of sugarcane | *Ceratocystis paradoxa** | Talukder et al. 2007, Rahman et al. 2009 |
|  | Crown rot of banana | *Ceratocystis paradoxa* (*Thielaviopsis paradoxa*)*** | Alvindia and Natsuaki 2008 |
|  | Anthracnose of strawberry | *Colletotrichum acutatum** | Freeman et al. 2004 |
|  | Anthracnose of blueberry | *Colletotrichum acutatum* | Verma et al. 2006 |
|  | Anthracnose of pear, apple, sour cherry and tomato | *Colletotrichum acutatum** | Živković et al. 2010 |
|  | Anthracnose of soyabean | *Colletotrichum dematium** | Shovan et al. 2008 |
|  | Anthracnose of cowpea | *Colletotrichum destructivum** | Akinbode and Ikotun 2011 |
|  | Red rot of sugarcane | *Colletotrichum falcatum** | Suresh and Nelson 2016 |
|  | Anthracnose of rambutan | *Colletotrichum gloeosporioides** | Sivakumar et al. 2000 |
|  | Anthracnose disease of pear, apple, sour cherry and tomato | *Colletotrichum gloeosporioides** | Živković et al. 2010 |
|  | Anthracnose leaf spots of guava | *Colletotrichum gloeosporioides** | Pandey et al. 1993 |
|  | Anthracnose of bean | *Colletotrichum lindemuthianum** | Padder et al. 2010, Padder and Sharma 2011 |
|  | Crown rot of banana | *Colletotrichum musae** | Sangeetha et al. 2009 |
|  | Anthracnose of chilli | *Colletotrichum truncatum* (*Colletotrichum capsici*)*** | Vasanthakumari and Shivanna 2013 |
|  | Leaf spot of turmeric | *Colletotrichum truncatum* (*Colletotrichum capsici*)*** | Jagtap et al. 2013 |
|  | Anthracnose of pepper | *Colletotrichum truncatum* (*Colletotrichum capsici*) | Ekefan et al. 2009 |
|  | Leaf smut of cowpea | *Erratomyces patelii* (*Protomycopsis phaseoli*)*** | Adejumo et al. 1999 |
|  | Pink disease of rubber | *Erythricium salmonicolor* (*Corticium salmonicolor*) | Jollands 1983 |
|  | Crown rot of banana | *Fusarium fujikuro* (*Fusarium verticillioides*)*** | Alvindia and Natsuaki 2008 |
|  | Fusarium root rot and seedling blight of wheat | *Fusarium graminearum** | Dal Bello et al. 2002, Foroutan 2013 |
|  | Fusarium wilt of potato | *Fusarium oxysporum** | Pratella and Mari 1993 |
|  | Fusarium wilt of sweet pepper | *Fusarium oxysporum** | Sahi and Khalid 2007 |
|  | Basal rot of onion | *Fusarium oxysporum** | Lee et al. 2001 |
|  | Basal rot disease of onion | *Fusarium oxysporum* f. sp. *cepae** | Coşkuntuna and Özer 2008 |
|  | Fusarium wilt (Panama wilt disease) of banana | *Fusarium oxysporum* f. sp. *cubense* | Thangavelu et al. 2004 |
|  | Fusarium wilt of tomato | *Fusarium oxysporum* f. sp. *lycopersici** | El-Rafai et al. 2003 |
|  | Fusarium crown and root rot of tomato | *Fusarium oxysporum* f. sp. *radicis-lycopersici** | Marois et al. 1981, Sivan et al. 1987, Sivan and Chet 1993, Datnoff et al. 1995, Mohamed and Haggag 2006 |
|  | Fusarium wilt of potato | *Fusarium oxysporum* f. sp. *tuberosi* | Ommati et al. 2013 |
|  | Fusarium wilt of pigeonpea | *Fusarium udum** | Prasad et al. 2002 |
|  | Basal stem rot of oil palm | *Ganoderma boninense** | Abdullah et al. 2003 |
|  | Basal stem rot disease of coconut | *Ganoderma lucidum** | Bhaskaran 2000 |
|  | Basal stem rot disease of oil palm | *Ganoderma orbiforme* (*Ganoderma boninense*)*** | Susanto et al. 2005 |
|  | Red root disease of rubber | *Ganoderma philippii* (*Ganoderma pseudoferreum*)*** | Ogbebor et al. 2010 |
|  | Brown spot of rambutan | *Gliocephalotrichum microchlamydosporum** | Sivakumar et al. 2000 |
|  | Charcoal stump rot of tea | *Kretzschmaria zonata* (*Ustulina zonata*)*** | Hazarika et al. 2000 |
|  | Postharvest rot of banana | *Lasiodiplodia theobromae* | Mortuza and Ilag 1999 |
|  | Crown rot of banana | *Lasiodiplodia theobromae** | Sangeetha et al. 2009 |
|  | _ | *Lasiodiplodia theobromae** | Bhadra et al. 2014 |
|  | Stem end rot of rambutan | *Lasiodiplodia theobromae* (*Botryodiplodia theobromae*)*** | Sivakumar et al. 2000 |
|  | Brown-rot of wood | *Lentoporia carbonica* (*Antrodia carbonica*)*** | Highley and Ricard 1988 |
|  | Root-rot of mungbean | *Macrophomina phaseolina** | Shahid and Khan 2016 |
|  | Charcoal rot of sunflower and mung bean | *Macrophomina phaseolina** | Hussain et al. 1990 |
|  | Charcoal rot of bean | *Macrophomina phaseolina** | Elad et al. 1986 |
|  | Postharvest disease of fruit and vegetable | *Macrophomina phaseolina** | Odebode 2006 |
|  | Witches' broom disease of cocoa | *Moniliophthora perniciosa* (*Crinipellis perniciosa*) | De Marco and Felix 2002 |
|  | Blue mould of grape | *Penicillium expansum* | Batta 2007 |
|  | Blue mould of apple | *Penicillium expansum* | Batta 2004 |
|  | Blue mould of Pear | *Penicillium expansum** | Batta 2007 |
|  | Blue mould of Kiwifruit | *Penicillium expansum** | Batta 2007 |
|  | Pestalotia leaf spots of guava | *Pestalotia psidii** | Pandey et al. 1993 |
|  | Soybean seed decay | *Phomopsis phaseoli* (*Diaporthe phaseolorum var. sojae*)*** | Begum et al. 2008 |
|  | Fruit rot of brinjal | *Phomopsis vexans** | Das et al. 2014 |
|  | Damping-off disease of sugar beet | *Pleospora bjoerlingii* (*Phoma betae*) | Grondona et al. 1997 |
|  | Powdery mildew of strawberry | *Podosphaera aphani, Podosphaera macularis* (*Sphaerotheca macularis*) | Pertot et al. 2008 |
|  | Powdery mildew of cucumber | *Podosphaera fusca* (*Sphaerotheca fusca*)*** | Elad et al. 1998, Elad 2000 |
|  | Downy mildews of cucumber | *Pseudoperonospora cubensis** | Elad 2000, El-Sharkaway et al. 2014 |
|  | Bird’s eye spot disease of tea | *Pseudocercospora ocellata* (*Cercospora theae*) | Gnanamangai and Ponmurugan 2012 |
|  | Root rot of apple seedlings | *Phytophthora cactorum** | Alexander and Stewart 2001 |
|  | Pink rot of potato & root and stem rot of tomato | *Phytophthora erythroseptica** | Etebarian et al. 2000 |
|  | Phytophthora leaf fall | *Phytophthora palmivora** | Promwee et al. 2017 |
|  | Tan spot of wheat | *Pyrenophora tritici-repentis ** | Perello et al. 2003, 2006 |
|  | Seed rot and damping-off of chickpea | *Rhizoctonia solani** | Prasad and Rangeshwaran 2000 |
|  | Stem canker of potato | *Rhizoctonia solani** | Brewer and Larkin 2005 |
|  | Sheath blight disease of rice | *Rhizoctonia solani** | Mishra et al. 2009 |
|  | Seedling blight of mung bean | *Rhizoctonia solani** | Bhagat and Pan 2007 |
|  | Damping-off disease of sugar beet | *Rhizoctonia solani* | Grondona et al. 1997 |
|  | Rhizopus rot of apple | *Rhizopus stolonifer** | Batta 2007 |
|  | Rhizopus rot of pear | *Rhizopus stolonifer** | Batta 2007 |
|  | Rhizopus rot of strawberry | *Rhizopus stolonifer* | Batta 2007 |
|  | Brown-rot of wood | *Rhodonia placenta* (*Postia placenta*) | Highley and Ricard 1988 |
|  | White root disease of rubber | *Rigidoporus microporus** | Jayasuriya and Thennakoon 2007, Kaewchai and Soytong 2010 |
|  | Sclerotinia rot of soybean | *Sclerotinia sclerotiorum** | Menendez et al. 1998 |
|  | Sclerotinia rot of lettuce | *Sclerotinia sclerotiorum* | Gracia-Garza et al. 1997 |
|  | Sclerotinia stem rot of rapesee | *Sclerotinia sclerotiorum** | Liu and Wen 2005 |
|  | White mould of cucumber | *Sclerotinia sclerotiorum** | Elad 2000 |
|  | Verticillium wilt of tomato | *Verticillium albo-atrum* | Jabnoun-Khiareddine et al. 2009 |
|  | Verticillium wilt of eggplant | *Verticillium dahliae** | Marois et al. 1982 |
|  | Verticillium wilt of tomato | *Verticillium dahliae** | El-Rafai et al. 2003, Jabnoun-Khiareddine et al. 2009 |
|  | Verticillium wilt of potato | *Verticillium dahliae** | Ordentlich et al. 1990 |
|  | Verticillium wilt of strawberry | *Verticillium dahliae** | Mirmajlessi et al. 2016 |
|  | Verticillium wilt of tomato | *Verticillium tricorpus* | Jabnoun-Khiareddine et al. 2009 |
|  | Karnal bunt of wheat | *Tilletia indica* | Sharma and Basandrai 2000 |
|  | Leaf blotch of wheat | *Zymoseptoria tritici* (*Mycosphaerella graminicola*) | Perello et al. 2006, 2009 |
|  | Stem bleeding of coconut | *Ceratocystis paradoxa* (*Thielaviopsis paradoxa*) | Kannangara et al. 2017 |
| *Trichoderma harzianum* mutant KA159-2 | Phytophthora root rot of avocado | *Phytophthora cinnamomi** | Costa et al. 2000 |
| *Trichoderma hebeiense* | _ | *Botrytis cinerea* | Chen and Zhuang 2017 |
|  | _ | *Sclerotinia sclerotiorum* | Chen and Zhuang 2017 |
| *Trichoderma koningii* | Seed-borne disease of radish | *Alternaria brassicicola, A. raphani,* | Vannacci and Harman 1987 |
|  | Postharvest disease of fruit and vegetable | *Alternaria solani** | Shaikh and Nasreen 2013 |
|  | Postharvest disease of fruit and vegetable | *Aspergillus niger** | Odebode 2006 |
|  | Southern blight disease of tomato | *Athelia rolfsii* (*Sclerotium rolfsii*)*** | Latunde‐Dada 1993 |
|  | Crown rot of banana | *Colletotrichum musae** | Sangeetha et al. 2009 |
|  | Anthracnose of chilli | *Colletotrichum truncatum* (*Colletotrichum capsici*)*** | Vasanthakumari and Shivanna 2013 |
|  | Leaf spot of turmeric | *Colletotrichum truncatum* (*Colletotrichum capsici*)*** | Jagtap et al. 2013 |
|  | Leaf smut of cowpea | *Erratomyces patelii* (*Protomycopsis phaseoli*)*** | Adejumo et al. 1999 |
|  | Pink canker of apple stems and twigs | *Erythricium salmonicolor* (*Corticium salmonicolor*)*** | Durga et al. 2014 |
|  | Postharvest disease of fruit and vegetable | *Fusarium oxysporum** | Shaikh and Nasreen 2013 |
|  | Postharvest disease of fruit and vegetable | *Neocosmospora solani* (*Fusarium solani*)*** | Odebode 2006, Shaikh and Nasreen 2013 |
|  | Crown rot of banana | *Lasiodiplodia theobromae** | Sangeetha et al. 2009, Bhadra et al. 2014 |
|  | Postharvest disease of fruit and vegetable | *Macrophomina phaseolina** | Odebode 2006, Shaikh and Nasreen 2013 |
|  | Damping-off disease of cowpea | *Macrophomina phaseolina** | Adekunle et al. 2001, Rajesh et al. 2007 |
|  | Witches' broom disease of cocoa | *Moniliophthora perniciosa* (*Crinipellis perniciosa*) | Krauss and Soberanis 2001 |
|  | Soybean seed decay | *Phomopsis phaseoli* (*Diaporthe phaseolorum var. sojae*)*** | Begum et al. 2008 |
|  | Root rot of apple seedlings | *Phytophthora cactorum** | Alexander and Stewart 2001 |
|  | Tan spot of wheat | *Pyrenophora tritici-repentis* | Perello et al. 2003, 2006, 2009 |
|  | Postharvest disease of fruit and vegetable | *Rhizoctonia solani** | Shaikh and Nasreen 2013 |
|  | Sclerotinia rot of lettuce | *Sclerotinia sclerotiorum** | Gracia-Garza et al. 1997 |
|  | Sclerotinia stem rot of rapesee | *Sclerotinia sclerotiorum** | Liu and Wen 2005 |
|  | White rot of onion | *Stromatinia cepivora* (*Sclerotium cepivorum*)*** | Metcalf 1997, Metcalf and Wilson 2001 |
|  | Leaf blotch of wheat | *Zymoseptoria tritici* (*Mycosphaerella graminicola*) | Perello et al. 2006 |
| *T. koningii,T. aureoviride,T. longibrachiatum* | Head rot of sunflower | *Sclerotinia sclerotiorum** | Escande et al. 2002 |
| *Trichoderma lixii* (*Hypocrea lixii*) | Anthracnose of mango | *Colletotrichum gloeosporioides** | de los Santos-Villalobos et al. 2013 |
|  | White root disease of rubber | *Rigidoporus microporus** | Ogbebor et al. 2015 |
| *Trichoderma longibrachiatum* | Grey mould of strawberry | *Botrytis cinerea** | Freeman et al. 2004 |
|  | Anthracnose of strawberry | *Colletotrichum acutatum** | Freeman et al. 2004 |
|  | Fusarium wilt of potato | *Fusarium oxysporum* f. sp. *tuberosi* | Ommati et al. 2013 |
|  | Soybean seed decay | *Phomopsis phaseoli* (*Diaporthe phaseolorum var. sojae*)*** | Begum et al. 2008 |
|  | Grey blight of tea | *Pseudopestalotiopsis theae* (*Pestalotiopsis theae*)*** | Barman et al. 2015 |
| *Trichoderma martiale* | Black-pod rot of cacao | *Phytophthora palmivora* | Hanada et al. 2009, 2010 |
| *Trichoderma ovalisporum* | Frosty pod rot of cocoa | *Moniliophthora roreri* (*Crinipellis roreri*) | Holmes et al. 2004 |
| *Trichoderma polysporum* | Stem bleeding of coconut | *Ceratocystis paradoxa** (*Thielaviopsis paradoxa*) | Kannangara et al. 2017 |
| *Trichoderma piluliferum* | Leaf spot disease of toothache plant (*Spilanthes oleracea*) | *Alternaria alternata** | Thakur and Harsh 2014 |
| *Trichoderma pseudokoningii* | Anthracnose of cowpea | *Colletotrichum destructivum** | Akinbode and Ikotun 2011 |
|  | Crown rot of banana | *Colletotrichum musae** | Sangeetha et al. 2009 |
|  | Crown rot of banana | *Lasiodiplodia theobromae* | Sangeetha et al. 2009 |
|  | Black pod rot of cacao | *Phytophthora palmivora* | Krauss and Soberanis 2001 |
|  | Sclerotinia stem rot of rapeseed | *Sclerotinia sclerotiorum** | Liu and Wen 2005 |
|  | White rot of onion | *Stromatinia cepivora* (*Sclerotium cepivorum*) | Clarkson et al. 2004 |
| *Trichoderma reesei* | Crown rot of banana | *Colletotrichum musae** | Sangeetha et al. 2009 |
|  | Crown rot of banana | *Lasiodiplodia theobromae** | Sangeetha et al. 2009 |
|  | Grey blight of tea | *Pseudopestalotiopsis theae* (*Pestalotiopsis theae*)*** | Barman et al. 2015 |
| *Trichoderma reesei* (*Hypocrea jecorina*) | Anthracnose of mango | *Colletotrichum gloeosporioides** | de los Santos-Villalobos et al. 2013 |
|  | White root disease of rubber | *Rigidoporus microporus** | Ogbebor et al. 2015 |
| *Trichoderma* sp. CEF-716 | Verticillium wilt of cotton | *Verticillium dahliae* | Li et al. 2014 |
| *Trichoderma spirale* | Anthracnose of mango | *Colletotrichum gloeosporioides** | de los Santos-Villalobos et al. 2013 |
|  | White root disease of rubber | *Rigidoporus microporus** | Ogbebor et al. 2015 |
| *Trichoderma strictipile* | Red root disease of rubber | *Ganoderma philippii* (*Ganoderma pseudoferreum*)*** | Ogbebor et al. 2010 |
| *Trichoderma stromaticum* | Witches' broom disease of cocoa | *Moniliophthora perniciosa* (*Crinipellis perniciosa*) | Samuels et al. 2000, Sanogo et al. 2002 |
| *Trichoderma virens* | Crown rot of banana | *Colletotrichum musae** | Sangeetha et al. 2009 |
|  | Fusarium corm rot and wilt of gladiolus | *Fusarium oxysporum* f. sp. *gladioli ** | Mishra et al. 2000 |
|  | Fusarium wilt of potato | *Fusarium oxysporum* f. sp. *tuberosi** | Ommati et al. 2013 |
|  | Crown rot of banana | *Lasiodiplodia theobromae* | Sangeetha et al. 2009 |
|  | Frosty pod rot of cacao | *Moniliophthora roreri** | Krauss and Soberanis 2001 |
|  | Soybean seed decay | *Phomopsis phaseoli* (*Diaporthe phaseolorum var. sojae*)*** | Begum et al. 2008 |
|  | Grey blight of tea | *Pseudopestalotiopsis theae* (*Pestalotiopsis theae*)*** | Barman et al. 2015 |
|  | Phytophthora crown and rot of apple | *Phytophthora cactorum** | Roiger and Jeffers 1991 |
|  | Pink rot of potato and root and stem rot of tomato | *Phytophthora erythroseptica** | Etebarian et al. 2000 |
|  | Seed-borne disease of radish | *Rhizoctonia solani** | Lee 2018 |
|  | Stem canker and black scurf of potato | *Rhizoctonia solani** | Brewer and Larkin 2005 |
|  | White mould of dry bean | *Sclerotinia sclerotiorum* | Huang et al. 2000 |
|  | Verticillium wilt of tomato | *Verticillium albo-atrum** | Jabnoun-Khiareddine et al. 2009 |
|  | Verticillium wilt of cotton | *Verticillium dahliae* | Hanson 2000 |
|  | Verticillium wilt of tomato | *Verticillium dahliae** | Jabnoun-Khiareddine et al. 2009 |
|  | Verticillium wilt of tomato | *Verticillium tricorpus** | Jabnoun-Khiareddine et al. 2009 |
| *Trichoderma virens* (*Hypocrea virens*) | White root disease of rubber | *Rigidoporus microporus** | Ogbebor et al. 2015 |
| *Trichoderma virens* (*Gliocladium virens*) | Alternaria blight of chickpea | *Alternaria alternata** | Agarwal et al. 2011 |
|  | Damping-off and blight of snapbean | *Athelia rolfsii* (*Sclerotium rolfsii*)*** | Papavizas and Lewis 1989 |
|  | Anthracnose of bean | *Colletotrichum lindemuthianum** | Padder and Sharma 2011 |
|  | White rot of wood | *Coriolus versicolor** | Highley and Ricard 1988 |
|  | Fusarium wilt of chickpea | *Fusarium oxysporum** | Agarwal et al. 2011 |
|  | Fusarium wilt of tomato | *Fusarium oxysporum* f. sp. l*ycopersici** | Larkin and Fravel 1998 |
|  | Damping-off disease of corn | *Globisporangium ultimum* (*Pythium ultimum*)@*, Fusarium graminearu, Pythium arrhenomane*@ | Mao et al. 1997 |
|  | Brown rot of wood | *Gloeophyllum trabeum** | Highley and Ricard 1988 |
|  | Brown rot of wood | *Lentoporia carbonica* (*Antrodia carbonica*)*** | Highley and Ricard 1988 |
|  | Charcoal rot of sunflower and mung bean | *Macrophomina phaseolina** | Hussain et al. 1990 |
|  | Brown rot of wood | *Neolentinus lepideus* (*Lentinus lepideus*)*** | Highley and Ricard 1988 |
|  | Brown rot of wood | *Rhodonia placenta* (*Postia placenta*)*** | Highley and Ricard 1988 |
|  | Rhizoctonia stem canker and black scurf of potato | *Rhizoctonia solani** | Beagle-Ristaino and Papavizas 1985 |
|  | Charcoal stump rot of tea | *Ustulina zonata** | Hazarika et al. 2000 |
|  | Verticillium wilt of eggplant | *Verticillium dahliae** | Marois et al. 1982 |
| *Trichoderma virens* (*Gliocladium virens*) mutant KA230-1 | Phytophthora root rot of avocado | *Phytophthora cinnamomi** | Costa et al. 2000 |
| *Trichoderma viride* | Fruit rot disease of chilli | *Alternaria alternata* | Anand et al. 2009 |
|  | Alternaria leaf spot of mung bean | *Alternaria alternata** | Mishra et al. 2011 |
|  | Alternaria blight of pigeon- pea | *Alternaria alternata** | Pradeep and Kumud 2000 |
|  | Alternaria blight of indian mustard | *Alternaria brassicae** | Meena et al. 2004 |
|  | Black rot of lemon | *Alternaria citri** | Pratella and Mari 1993 |
|  | Collar rot of groundnut | *Aspergillus niger** | Devi and Prasad 2009, Kumari and Singh 2017 |
|  | Postharvest rot of yam | *Aspergillus niger*, Lasiodiplodia theobromae* (*Botryodiplodia theobromae*)**, Penicillium oxalicum** | Okigbo and Ikediugwu 2000 |
|  | Root rot of mung bean | *Athelia rolfsii* (*Sclerotium rolfsii*)**, Rhizoctonia solani, Macrophomina phaseolin* | Mishra et al. 2011 |
|  | Basal stem rot disease of tomato | *Athelia rolfsii* (*Corticium rolfsii*)*** | Wokocha et al. 1986 |
|  | Brown spot disease of rice | *Bipolaris oryzae** | Harish et al. 2008 |
|  | Grey mould of strawberry | *Botrytis cinerea** | Pratella and Mari 1993 |
|  | Cladosporium leaf spot of pigeon pea | *Cladosporium cladosporioides** | Pradeep and Kumud 2000 |
|  | Seed rot and seedling diseases of pigeon pea | *Colletotrichum dematium** | Pradeep and Kumud 2000 |
|  | Anthracnose of soyabean | *Colletotrichum dematium** | Saber et al. 2003 |
|  | Red rot of sugarcane | *Colletotrichum falcatum** | Suresh and Nelson 2016 |
|  | Fruit rot disease of chilli | *Colletotrichum gloeosporioides** | Ngullie et al. 2010 |
|  | Leaf twisting of onion | *Colletotrichum gloeosporioides* | Naguleswaran et al. 2014 |
|  | Anthracnose of bean | *Colletotrichum lindemuthianum** | Padder et al. 2010, Padder and Sharma 2011 |
|  | Anthracnose of cowpea | *Colletotrichum lindemuthianum** | Adebanjo and Bankole 2004 |
|  | Crown rot of banana | *Colletotrichum musae** | Sangeetha et al. 2009 |
|  | Brown blotch of cowpea | *Colletotrichum truncatum** | Bankole and Adebanjo 1996 |
|  | Leaf spot of turmeric | *Colletotrichum truncatum (Colletotrichum capsici)** | Jagtap et al. 2013 |
|  | Anthracnose of mung bean | *Colletotrichum truncatum* (*Colletotrichum capsici*)*** | Mishra et al. 2011 |
|  | Anthracnose of chilli | *Colletotrichum truncatum* (*Colletotrichum capsici*) | Anand et al. 2009 |
|  | Corynespora leaf disease of rubber | *Corynespora cassiicola** | Evueh and Osemwegie 2011 |
|  | Chestnut blight | *Cryphonectria parasitica** | Arisan-Atac et al. 1995 |
|  | Curvularia blight of pigeon pea | *Curvularia lunata** | Pradeep and Kumud 2000 |
|  | Cankers, vascular necrosis and dieback on oak | *Diplodia corticola* | Campanile et al. 2007 |
|  | Powdery mildew of spindle | *Erysiphe euonymi-japonici* (*Oidium euonymi-japonici*) | Ahanger et al. 2018 |
|  | Pink canker of apple stems and twigs | *Erythricium salmonicolor* (*Corticium salmonicolor*)*** | Durga et al. 2014 |
|  | Root and stalk rot of maize | *Fusarium fujikuroi* (*Fusarium moniliforme*)*** | Yates et al. 1999, Bacon et al. 2001 |
|  | Fusarium wilt of potato | *Fusarium oxysporum** | Pratella and Mari 1993 |
|  | Fusarium basal rot of onion | *Fusarium oxysporum* (*Fusarium oxysporum* f. sp. *cepae)* | Naguleswaran et al. 2014 |
|  | Fusarium wilt (Panama wilt disease) of banana | *Fusarium oxysporum* f. sp. *cubense** | Raguchander et al. 2000 |
|  | Fusarium wilt of soybean/ Adzuki bean wilt | *Fusarium oxysporum* f. sp. *adzuki* | John et al. 2010 |
|  | Basal stem rot disease of oil palm | *Ganoderma orbiforme* (*Ganoderma boninense*)*** | Susanto et al. 2005 |
|  | Charcoal stump rot of tea | *Kretzschmaria zonata* (*Ustulina zonata*)*** | Hazarika et al. 2000 |
|  | Stem-end rot of mango | *Lasiodiplodia theobromae* (*Diplodia natalensis*)*** | Moreno and Paningbatan 1995 |
|  | Postharvest rot of banana | *Lasiodiplodia theobromae* | Mortuza and Ilag 1999 |
|  | _ | *Lasiodiplodia theobromae** | Bhadra et al. 2014 |
|  | Crown rot of banana | *Lasiodiplodia theobromae** | Sangeetha et al. 2009 |
|  | Inflorescence blight disease of cashew | *Lasiodiplodia theobromae** | Adeniyi et al. 2013 |
|  | Dry rot of black gram | *Macrophomina phaseolina** | Karthikeyan et al. 2015 |
|  | Leaf spot and blight disease of pigeon pea | *Macrophomina phaseolina* (*Rhizoctonia bataticola*)*** | Pradeep and Kumud 2000 |
|  | Phyllosticta leaf spot of pigeon pea | *Phyllosticta cajani** | Pradeep and Kumud 2000 |
|  | Late blight of potato | *Phytophthora infestans* | Zegeye et al. 2011 |
|  | Grey blight of tea | *Pseudopestalotiopsis theae* (*Pestalotiopsis theae*)*** | Barman et al. 2015 |
|  | Damping-off diseases of soybean | *Pythium arrhenomanes*@ | John et al. 2010 |
|  | Rhizoctonia stem canker and black scurf of potato | *Rhizoctonia solani** | Beagle-Ristaino and Papavizas 1985 |
|  | Web blight of pigeonpea | *Rhizoctonia solani** | Pradeep and Kumud 2000 |
|  | Black scurf disease of potato | *Rhizoctonia solani* | Somani and Arora 2010 |
|  | Sclerotinia rot of lettuce | *Sclerotinia sclerotiorum** | Gracia-Garza et al. 1997 |
|  | Sclerotinia stem rot of rapesee | *Sclerotinia sclerotiorum** | Liu and Wen 2005 |
|  | White mould of bean | *Sclerotinia sclerotiorum** | Boland and Inglis 1989 |
|  | Sugarcane smut | *Sporisorium scitamineum** | Lal et al. 2009 |
|  | White rot of onion | *Stromatinia cepivora* (*Sclerotium cepivorum*)*** | Harrison and Stewart 1988, Clarkson et al. 2002, 2004 |
|  | Karnal bunt of wheat | *Tilletia indica** | Sharma and Basandrai 2000 |
|  | Seed rot and seedling diseasesp of pigeon pea | *Trichothecium roseum** | Pradeep and Kumud 2000 |
|  | Verticillium wilt of hops | *Verticillium albo-atrum** | Solarska et al. 2001 |
|  | Verticillium wilt of tomato | *Verticillium albo-atrum** | Dutta et al. 1981, Deketelaere et al. 2017 |
|  | Verticillium wilt of eggplant | *Verticillium dahliae** | Marois et al. 1982 |
|  | Verticillium wilt of tomato | *Verticillium dahliae** | Jabnoun-Khiareddine et al. 2009 |
|  |  | *Verticillium tricorpus* | Jabnoun-Khiareddine et al. 2009 |
|  | Stem bleeding of coconut | *Ceratocystis paradoxa** (*Thielaviopsis paradoxa*) | Kannangara et al. 2017 |
| *Trichoderma viridescens* | White root disease of rubber | *Rigidoporus microporus** | Ogbebor et al. 2015 |
| *Trichosporon* sp.IMI 382212 and 382211 | Grey mould of apple | *Botrytis cinerea** | Tian et al. 2002a |
| *Trichosporon* sp.IMI 382212 and 382211 | Grey mould rot of pear | *Botrytis cinerea* | Tian et al. 2002a |
| *Trichosporon* sp.IMI 382212 and 382211 | Blue mould of apple | *Penicillium expansum** | Tian et al. 2002a |
| *Trichosporon* sp.IMI 382212 and 382211 | Blue mould of pear | *Penicillium expansum* | Tian et al. 2002a |
| *Trichothecium roseum* | Soybean rust | *Phakopsora pachyrhizi* | Kumar and Jha 2002 |
|  | White mould of dry bean | *Sclerotinia sclerotiorum* | Huang et al. 2000 |
|  | Verticillium wilt of cotton | *Verticillium dahliae* | Li et al. 2014 |
| *Typhula phacorrhiza* | Snow mould diseased of creeping bent grass | *Typhula ishikariensi*, Typhula incarnata** | Wu and Hsiang 1998, Wu et al. 1998 |
| *Verticillium albo-atrum* | Verticillium wilt of cotton | *Verticillium dahliae** | Schnatho. and Mathre 1966, Deketelaere et al. 2017 |
|  | Verticillium wilt of tomato | *Verticillium dahliae** | Matta and Garibaldi 1977 |
| *Verticillium biguttatum* | Stem canker of potato | *Rhizoctonia solani** | Brewer and Larkin 2005 |
| *Verticillium dahliae#* | Verticillium wilt of cotton | *Verticillium dahliae** | Zhu et al. 2013 |
|  | Verticillium wilt of tomato | *Verticillium dahliae** | Shittu et al. 2009 |
| *Verticillium isaacii* | Verticillium wilt of cauliflower | *Verticillium longisporum** | França et al. 2013, Tyvaert et al. 2014 |
| *Verticillium tricorpus* | Verticillium wilt of tomato | *Verticillium dahliae** | Matta and Garibaldi 1977 |
| *Verticillium tricorpus* | Crown rot of banana | *Ceratocystis paradoxa* (*Thielaviopsis paradoxa*) | Alvindia and Natsuaki 2008 |
|  |  | *Colletotrichum musae* | Alvindia and Natsuaki 2008 |
|  |  | *Lasiodiplodia theobromae* | Alvindia and Natsuaki 2008 |
|  | Verticillium wilt of lettuce | *Verticillium dahliae** | Qin et al. 2008 |
|  | Verticillium wilt of artichoke | *Verticillium dahliae** | Qin et al. 2008 |
| *Waitea circinata* (*Rhizoctonia zeae*) | Black scurf of potato | *Rhizoctonia solani** | Brewer and Larkin 2005 |
|  | Stem canker of potato | *Rhizoctonia solani* | Brewer and Larkin 2005 |
| *Wickerhamomyces anomalus* | Grey mould of apple | *Botrytis cinerea** | Pretscher et al. 2018 |
|  | Post-harvest bunch rot of table grape | *Botrytis cinerea** | Parafati et al. 2015 |
|  | Green mould of citrus | *Penicillium digitatum** | Perez et al. 2016 |
|  | Blue mould of citrus | *Penicillium italicum** | Perez et al. 2016 |
|  | Root rot of apple | *Roesleria subterranea** | Pretscher et al. 2018 |
| *Wickerhamomyces anomalus* (*Pichia anomala*) | Crown rot of banana | *Colletotrichum musae* | Lassois et al. 2008 |
|  | Sour rot decay of citrus | *Dipodascus geotrichum* (*Geotrichum candidum*)*** | Lahlali et al. 2004 |
|  | Eutypa dieback of grapes | *Eutypa lata** | Pretscher et al. 2018 |
|  | Crown rot of banana | *Fusarium fujikuroi* (*Fusarium moniliforme*) | Lassois et al. 2008 |
|  | Diplodia rot of guava | *Lasiodiplodia theobromae* (*Botryodiplodia theobromae*)*** | Mohamed and Saad 2009 |
|  | Brown rot of apple | *Monilinia fructigena** | Pretscher et al. 2018 |
|  | Green mould of citrus | *Penicillium digitatum** | Lahlali et al. 2004, |
|  | Blue mould of citrus | *Penicillium italicum** | Lahlali et al. 2004 |
| *Xylaria hypoxylon* | Armillaria root rot of peach | *Armillaria mellea and Desarmillaria tabescens* (*Armillaria tabescens*) *** | Cox and Scherm 2006 |
| *Zygosaccharomyces bailii* | Seedling blight and blast of rice | *Magnaporthe oryzae** | Pretscher et al. 2018 |

# non pathogenic strains (cross protection); * antagonists suppressed the disease/pathogen by equal or greater than 50% (the maximum inhibition under *in vitro*, *in vivo*, greenhouse or field condition is considered); @ *fungal-like* species (Oomycota, Chromista); Current names of the fungal species are given here (used names in each particular research paper are mentioned in brackets where applicable).

**REFERENCES**

Abdullah, F., Ilias, G. N. M., Nelson, M., Izzati, N. A. M. Z., and Yusuf, U. K. (2003). Disease asessment and the efficacy of *Trichoderma* as a biocontrol agent of basal stem rot of oil palms. *Res. Bull. Sci. Putra*11, 31–33.

Adams, P. B., and Ayers, W. A. (1982). Biological control of Sclerotinia lettuce drop in thefield by *Sporidesmium sclerotivorum*. *Phytopathology*  72 (5), 485–488. doi: 10.1094/Phyto-72-485

Adams, P. B., and Fravel, D. R. (1990). Economical biological control of Sclerotinia lettuce drop by *Sporidesmium sclerotivorum*. *Phytopathology*  80 (10), 1120–1124. doi: 10.1094/Phyto-80-1120

Adebanjo, A., and Bankole, S. A. (2004). Evaluation of some fungi and bacteria for biocontrol of anthracnose disease of cowpea. *J. Basic Microbiol*. 44 (1), 3–9. doi: 10.1002/jobm.200310310

Adejumo, T. O., Ikotun, T., and Florini, D. A. (1999). Biological control of *Protomycopsis phaseoli*, the causal agent of leaf smut of cowpea. *J. Phytopathol.* 147 (6), 371–375. doi: 10.1046/j.1439-0434.1999.00371.x

Adekunle, A. T., Cardwell, K. F., Florini, D. A., and Ikotun, T. (2001). Seed treatment with *Trichoderma* species for control of damping-off of cowpea caused by *Macrophomina phaseolina*. *Biocontrol Sci. Technol.* 11 (4), 449–457. doi: 10.1080/0958315012006748

Adeniyi, D. O., Adedeji, A. R., Oduwaye, O. F., and Kolawole, O. O. (2013). Evaluation of biocontrol agents against *Lasiodiplodia theobromae* causing inflorescence blight of cashew in Nigeria. *IOSR. J. Agric. Vet. Sci*. 5 (3), 46–48. doi:10.9790/2380-0534648

Agarwal, T., Malhotra, A., Trivedi, P. C., and Biyani, M. (2011). Biocontrol potential of *Gliocladium virens* against fungal pathogens isolated from chickpea, lentil and black gram seeds. *J. Agric. Technol*. 7 (6), 1833–1839

Aggarwal, R., Tewari, A. K., Srivastava, K. D., and Singh, D. V. (2004). Role of antibiosis in the biological control of spot blotch (*Cochliobolus sativus*) of wheat by *Chaetomium globosum*. *Mycopathologia* 57 (4), 369–377. doi:10.1023/B:MYCO.0000030446.86370.14

Ahanger, R. A., Qazi, N. A., Bhat, H. A., Bhat, A. H., and Dar, N. A. (2018). Management of powdery mildew of *Euonymus japonicas* in Kashmir Valley. *Indian Phytopathol*.71 (3), 377–384. doi: 10.1007/s42360-018-0041-z

Adikaram, N. K. B., and Karunaratne, A. (1998). “Suppression of Avocado Anthracnose and Stem-end Rot Pathogens by Endogenous Antifunga1 Substances and a Surface Inhabiting *Pestalotiopsis* sp,” in ACIAR PROCEEDINGS (Australian centre for international agricultural research, Canberra, Australia), 72–77.

Akinbode, O. A., and Ikotun, T. (2011). Potentials of two *Trichoderma* species as antagonistic agents against *Colletotrichum destructivum* of cowpea. *African. J. Microbiol. Res.* 5 (5), 551–554. doi.org/10.5897/AJMR10.151

Alexander, B. J. R., and Stewart, A. (2001). Glasshouse screening for biological control agents of *Phytophthora cactorum* on apple (Malus domestica). *New Z. J. Crop Hortic. Sci.* 29 (3), 159–169. doi: 10.1080/01140671.2001.9514174

Alvindia, D. G., and Natsuaki, K. T. (2008). Evaluation of fungal epiphytes isolated from banana fruit surfaces for biocontrol of banana crown rot disease. *Crop Prot*. 27 (8), 1200–1207. doi: 10.1016/j.cropro.2008.02.007

Alström, S. (2000). Root-colonizing Fungi from Oilseed Rape and their Inhibition of *Verticillium dahliae*. *J. Phytopathol.* 148 (7-8), 417–423. doi: 10.1046/j.1439-0434.2000.00511.x

Anand, S., and Reddy, J. (2009). Biocontrol potential of *Trichoderma* sp. Against plant pathogens. *Int. J. Agric. Sci.* 1 (2), 30. doi: 10.9735/0975-3710.1.2.30-39

Andrade-Linares, D. R., Grosch, R., Restrepo, S., Krumbein, A., and Franken, P. (2011). Effects of dark septate endophytes on tomato plant performance. *Mycorrhiza* 21 (5), 413–422. doi: 10.1007/s00572-010-0351-1

Anita, S., and Ponmurugan, P. (2011). In vitro evaluation of *Trichoderma atroviride* against *Phomopsis theaea* casual agent of collar canker disease in tea plants. *Int. J. Agric. Res*. 6 (8), 620–631. doi: 10.3923/ijar.2011.620.631

Arisan-Atac, I., Heidenreich, E., and Kubicek, C. P. (1995). Randomly amplified polymorphic DNA fingerprinting identifies subgroups of *Trichoderma viride* and other *Trichoderma* sp. capable of chestnut blight biocontrol. *FEMS Microbiol. Lett*. 126 (3), 249–255. doi: 10.1111/j.1574-6968.1995.tb07426.x

Arras, G., Cicco, V. D., Arru, S., and Lima, G. (1998). Biocontrol by yeasts of blue mould of citrus fruits and the mode of action of an isolate of *Pichia guilliermondii*. *J. Hortic. Sci. Biotechnol*. 73 (3), 413–418. doi: 10.1080/14620316.1998.11510993

Askary, H., Carriere, Y., Belanger, R. R., and Brodeur, J. (1998). Pathogenicity of the fungus *Verticillium lecanii* to aphids and powdery mildew. *Biocontrol Sci. Technol*. 8 (1), 23–32. doi: 10.1080/09583159830405

Bacon, C. W., Yates, I. E., Hinton, D. M., and Meredith, F. (2001). Biological control of *Fusarium moniliforme* in maize.Environ. Health Perspect.109 (suppl 2), 325–332. doi.org/10.1289/ehp.01109s2325

Bankole, S. A., and Adebanjo, A. (1996). Biocontrol of brown blotch of cowpea caused by *Colletotrichum truncatum* with.*Trichoderma Viride* *Crop Protect*. 15 (7), 633–636. doi: 10.1016/0261-2194(96)00028-2

Barman, H., Roy, A., and Das, S. K. (2015). Evaluation of plant products and antagonistic microbes against grey blight (*Pestalotiopsis theae*), a devastating pathogen of tea. *Afr. J. Microbiol. Res.* 9 (18), 1263–1267. doi.org/10.5897/AJMR2015.7391

Batta, Y. A. (2001). Effect of fungicides and antagonistic microorganisms on the black fruit spot disease on persimmon. *Dirasat.: Agric. Sci*. 28 (2&3), 165–171.

Batta, Y. A. (2004). Effect of treatment with *Trichoderma harzianum* Rifai formulated in invert emulsion on postharvest decay of apple blue mold. *Int. J. Food Microbiol*. 96 (3), 281–288. doi: 10.1016/j.ijfoodmicro.2004.04.002

Batta, Y. A. (2007). Control of postharvest diseases of fruit with an invert emulsion formulation of *Trichoderma harzianum* Rifai. *Postharvest Biol. Technol*. 43 (1), 143–150. doi: 10.1016/j.postharvbio.2006.07.010

Beagle-Ristaino, J. E., and Papavizas, G. C. (1985). Biological control of Rhizoctonia stem canker and black scurf of potato. *Phytopathology* 75 (5), 560–564. doi: 10.1094/Phyto-75-560

Beale, R. E., and Pitt, D. (1990). Biological and integrated control of Fusarium basal rot of Narcissususing *Minimedusa polyspora* and other micro-organisms. *Plant Pathol*. 39 (3), 477–488. doi: 10.1111/j.1365-3059.1990.tb02524.x

Begum, M. M., Sariah, M., Abidin, M. Z., Puteh, A. B., and Rahman, M. A. (2008). Ultrastructural studies of Soybean seed-borne infection by *Diaporthe phaseolorum* var. *sojae* and screening of antagonistic potentiality by selected biocontrol agents *in vitro*. *Pertanika J. Trop. Agric. Sci*. 31 (2), 247–256.

Benbow, J. M., and Sugar, D. (1999). Fruit surface colonization and biological control of postharvest diseases of pear by preharvest yeast applications. *Plant Dis*. 83 (9), 839–844. doi: 10.1094/PDIS.1999.83.9.839

Benhamou, N., Garand, C., and Goulet, A. (2002). Ability of nonpathogenic Fusarium *oxysporum* strain Fo47 to induce resistance against *Pythium ultimum* infection in cucumber. *Appl. Environ. Microbiol*. 68 (8), 4044–4060. doi: 10.1128/AEM.68.8.4044-4060.2002

Bérubé, J. A., Trudelle, J. G., Carisse, O., and Dessureault, M. (1998). “Endophytic fungalflora from eastern white pine needles and apple tree leaves as a means of biological control for white pine blister rust,” in Proceedings, of 1^st^ IUFRO Rusts of Forest Trees Working Party Confe rence, Saariselkä, Finland. 2–7.

Bhadra, M., Khair, A., Hossain, M. A., and Sikder, M. M. (2014). Efficacy of *Trichoderma* spp. and fungicides against *Lasiodiplodia theobromae*. *Bangladesh J. Sci. Ind. Res*. 49 (2), 125–130. doi.org/10.3329/bjsir.v49i2.22008

Bhagat, S., and Pan, S. (2007). Mass multiplication of *Trichoderma harzianumon* agricultural byproducts and their evaluation against seedling blight (*Rhizoctonia solani*) of mungbean and collar rot (*Sclerotium rolfsii*) of groundnut. *Indian J. Agric. Sci*. 77 (9), 583-588.

Bhaskaran, R. (2000).“ Managment of the basal stem rot disease of coconut caused by *Ganoderma lucidum*,” in Ganoderma Diseases in Perennial Crops. Eds. J. Flood, P. D. Bridge and M. Holderness (Wallingford: CABI), 121–129

Biswas, S. K., Srivastava, K. D., Aggarwal, R., Prem, D., and Singh, D. V. (2000). Antagonism of *Chaetomium globosum* to *Drechslera sorokiniana*, the spot blotch pathogen of wheat. *Indian Phytopathol*. 53 (4), 436–440.

Bitsadze, N., Siebold, M., Koopmann, B., and von Tiedemann, A. (2015). Single and combined colonization of *Sclerotinia sclerotiorum* sclerotia by the fungal mycoparasites *Coniothyrium minitans* and *Microsphaeropsis Ochracea*. *Plant Pathol*. 64 (3), 690–700. doi: 10.1111/ppa.12302

Boland, G. J., and Inglis, G. D. (1989). Antagonism of white mold (*Sclerotinia sclerotiorum*) of bean by fungi from bean and rapeseed flowers. *Can. J. Bot*. 67 (6), 1775–1781. doi: 10.1139/b89-225

Bosshard, E., Schüepp, H., and Siegfried, W. (1987). Concepts and methods in biological control of diseases in apple orchards 1. *EPPO Bull*. 17 (4), 655–663. doi: 10.1111/j.1365-2338.1987.tb00087.x

Brewer, M. T., and Larkin, R. P. (2005). Efficacy of several potential biocontrol organisms against *Rhizoctonia solani* on potato. *Crop Prot*. 24 (11), 939–950. doi: 10.1016/j.cropro.2005.01.012

Budge, S. P., and Whipps, J. (1991). Glasshouse trials of *Coniothyrium minitans* and *Trichoderma* species for the biological control of *Sclerotinia sclerotiorum* in celery and lettuce. *Plant Pathol*. 40 (1), 59–66. doi: 10.1111/j.1365-3059.1991.tb02293.x

Campanile, G., Ruscelli, A., and Luisi, N. (2007). Antagonistic activity of endophytic fungi towards *Diplodia corticola* assessed by in vitro and in planta tests. *Eur. J. Plant Pathol*. 117 (3), 237–246. doi: 10.1007/s10658-006-9089-1

Cao, B., Li, H., Tian, S., and Qin, G. (2012). Boron improves the biocontrol activity of *Cryptococcus laurentii* against *Penicillium expansum* in jujube fruit. *Postharvest Biol. Technol*. 68, 16–21. doi: 10.1016/j.postharvbio.2012.01.008

Cao, J., Zhang, H., Yang, Q., and Ren, R. (2013). Efficacy of *Pichia caribbica* in controlling blue mold rot and patulin degradation in apples. *Int. J. Food Microbiol*. 162 (2), 167–173. doi: 10.1016/j.ijfoodmicro.2013.01.007

Card, S. D., Walter, M., Jaspers, M. V., Sztejnberg, A., and Stewart, A. (2009). Targeted selection of antagonistic microorganisms for control of *Botrytis cinerea* of strawberry in New Zealand. *Australas. Plant Pathol*. 38 (2), 183–192. doi: 10.1071/AP08097

Carrero-Carrón, I., Trapero-Casas, J. L., Olivares-Garcı́ a, C., Monte, E., Hermosa, R., and Jiménez-Dı́ az, R. M. (2016). *Trichoderma asperellum* is effective for biocontrol of Verticillium wilt in olive caused by the defoliating pathotype of *Verticillium dahliae*. *Crop Protect*. 88, 45–52. doi: 10.1016/j.cropro.2016.05.009

Chalutz, E., and Wilson, C. L. (1990). Postharvest biocontrol of green and blue mold and sour rot of citrus fruit by *Debaryomyces Hansenii. Plant Dis*. 74 (2), 134–137. doi : 10.1094/PD-74-0134

Chanchaichaovivat, A., Ruenwongsa, P., and Panijpan, B. (2007). Screening and identification of yeast strains from fruits and vegetables: Potential for biological control of postharvest chilli anthracnose (*Colletotrichum capsici*). *Biol. Control* 42 (3), 326–335. doi: 10.1016/j.biocontrol.2007.05.016

Chand-Goyal, T., and Spotts, R. A. (1996a). Postharvest biological control of blue mold of apple and brown rot of sweet cherry by natural saprophytic yeasts alone or in combination with low doses of fungicides. *Biol. Control* 6 (2), 253–259. doi: 10.1006/bcon.1996.0032

Chand-Goyal, T., and Spotts, R. A. (1996b). Control of postharvest pear diseases using natural saprophytic yeast colonists and their combination with a low dosage of thiabendazole. *Postharvest Biol. Technol*. 7 (1-2), 51–64. doi: 10.1016/0925-5214(95)00031-3

Chen, K., and Zhuang, W. Y. (2017). Three new soil-inhabiting species of *Trichoderma* in the Stromaticum clade with test of their antagonism to pathogens. *Curr. Microbiol.* 74 (9), 1049–1060. doi: 10.1007/s00284-017-1282-2

Chen, X., Li, J., Zhang, L., Xu, X., Wang, A., and Yang, Y. (2012). Control of postharvest radish decay using a Cryptococcus albidus yeast coating formulation. Crop Protect.41, 88–95. doi: 10.1016/j.cropro.2012.05.015

Chen, J. L., Sun, S. Z., Miao, C. P., Wu, K., Chen, Y. W., Xu, L. H., et al. (2016). Endophytic *Trichoderma gamsii* YIM PH30019: a promising biocontrol agent with hyperosmolar, mycoparasitism, and antagonistic activities of induced volatile organic compounds on root-rot pathogenic fungi of *Panax notoginseng*. *J. ginseng Res*. 40 (4), 315–324. doi: 10.1016/j.jgr.2015.09.006

Clarkson, J. P., Payne, T., Mead, A., and Whipps, J. M. (2002). Selection of fungal biological control agents of *Sclerotium cepivorum* for control of white rot by sclerotial degradation in a UK soil. *Plant Pathol*. 51 (6), 735–745. doi: 10.1046/j.1365-3059.2002.00787.x

Clarkson, J.P., Mead, A., Payne, T., and Whipps, J.M. (2004). Effectof environmental factors and *Sclerotium cepivorum* isolate on sclerotial degradation and biological control of white rot by *Trichoderma*. *Plant Pathol*. 53 (3), 353–362. doi: 10.1111/j.0032-0862.2004.01013.x

Conway, W. S., Leverentz, B., Janisiewicz, W. J., Blodgett, A. B., and Saftner, R. A. and Camp, M.J. (2004). Integrating heat treatment, biocontrol and sodium bicarbonate to reduce postharvest decay of apple caused by *Colletotrichum acutatum* and *Penicillium expansum*. *Postharvest Biol. Technol*. 34 (1), 11–20. doi.org/10.1016/j.postharvbio.2004.05.011

Cook, D. W. M., Long, P. G., and Ganesh, S. (1999). The combined effect of delayed application of yeast biocontrol agents and fruit curing for the inhibition of the postharvest pathogen *Botrytis cinerea* in kiwifruit. *Postharvest Biol. Technol*. 16 (3), 233–243. doi: 10.1016/S0925-5214(99)00003-4

Cordier, C., Pozo, M. J., Barea, J. M., Gianinazzi, S., and Gianinazzi-Pearson, V. (1998). Cell defense responses associated with localized and systemic resistance to *Phytophthora parasitica* induced in tomato by an arbuscular mycorrhizal fungus. *Mol. Plant-Microbe Interact*. 11 (10), 1017–1028. doi: 10.1094/MPMI.1998.11.10.1017

Coşkuntuna, A., and Özer, N. (2008). Biological control of onion basal rot disease using *Trichoderma harzianum* and induction of antifungal compounds in onion set following seed treatment.*Crop Prot*. 27 (3-5), 330–336. doi: 10.1016/j.cropro.2007.06.002

Costa, J. L. D. S., Menge, J. A., and Casale, W. L. (2000). Biological control of Phytophthora root rot of avocato with microorganisms grown in organic mulches. *Braz. J. Microbiol*. 31 (4), 239–246. doi: 10.1590/S1517-83822000000400002

Cotxarrera, L., Trillas-Gay, M. I., Steinberg, C., and Alabouvette, C. (2002). Use of sewage sludge compost and *Trichoderma asperellum* isolates to suppress *Fusarium wilt* of tomato. *Soil Biol. Biochem*. 34 (4), 467–476. doi: 10.1016/S0038-0717(01)00205-X

Cox, K. D., and Scherm, H. (2006). Interaction dynamics between saprobic lignicolous fungi and *Armillaria* in controlled environments: Exploring the potential for competitive exclusion of *Armillaria* on peach. *Biol. Control* 37 (3), 291–300. doi: 10.1016/j.biocontrol.2006.01.012

Dal Bello, G. M., Monaco, C. I., and Simon, M. R. (2002). Biological control of seedling blight of wheat caused by *Fusarium graminearum* with beneficial rhizosphere microorganisms. *World J. Microbiol. Biotechnol*. 18 (7), 627–636.doi: 10.1023/A:1016898020810

Datnoff, L. E., Nemec, S., and Pernezny, K. (1995). Biological control of Fusarium crown and root rot of tomato in Florida using *Trichoderma harzianum* and *Glomus intraradices*. *Biol. Control* 5 (3), 427–431. doi: 10.1006/bcon.1995.1051

Das, S. N., Sarma, T. C., and Tapadar, S. A. (2014). In vitro evaluation of fungicides and two species of *Trichoderma* against *Phomopsis vexans* causing fruit rot of brinjal (*Solanum melongena* L.). Int. J. Sci. Res. Publ.4 (9), 1–2.

De Cal, A., M.-Sagasta, E., and Melgarejo, P. (1990). Biological control of peach twig blight (*Monilinia laxa*) with *Penicillium frequentans*. *Plant Pathol*. 39 (4), 612–618. doi: 10.1111/j.1365-3059.1990.tb02542.x

De Cal, A., Larena, I., Guijarro, B., and Melgarejo, P. (2002). Mass production of conidia of *Penicillium frequentans*, a biocontrol agent against brown rot of stone fruits. *Biocontrol Sci. Technol*.12 (6), 715–725. doi: 10.1080/0958315021000039897

Deketelaere, S., Tyvaert, L., França, S. C., and Höfte, M. (2017). Desirable traits of a good biocontrol agent against Verticillium wilt. *Front. Microbiol*. 8, 1186. doi:10.3389/fmicb.2017.01186

De los Santos-Villalobos, S., Guzmán-Ortiz, D. A., Gómez-Lim, M. A., Délano Frier, J. P., de-Folter, S., Sánchez-Garcı́ a, P., et al. (2013). Potential use of *Trichoderma asperellum* (Samuels, Liechfeldt et Nirenberg) T8a as a biological control agent against anthracnose in mango (*Mangifera indica* L.). *Biol. Control* 64 (1), 37–44. doi: 10.1016/j.biocontrol.2012.10.006

De Marco, J. L., and Felix, C. R. (2002). Characterization of a protease produced by a *Trichoderma harzianum* isolate which controls cocoa plant witches’ broom disease. *BMC Biochem*. 3(1), 1-7. doi.org/10.1186/1471-2091-3-3

Demir, S., Şensoy, S., Ocak, E., Tüfenkci, Ş., Durak, E. D., Erdinc, C., et al. (2015). Effects of arbuscular mycorrhizal fungus, humic acid, and whey on wilt diseasecaused by *Verticillium dahlia* Kleb. in three solanaceous crops. *Turkish J. Agric. For*. 39 (2), 300–309. doi:10.3906/tar-1403-39

Devi, M. C., and Prasad, R. D. (2009). Biointensive management of collar rot of groundnut caused by *Aspergillus niger*. J. *Biol. Control* 23 (1), 21–24. doi.org/10.18311/jbc/2009/3610

Dharmaputra, O. S., Tjitrosomo, H. S. S., and Abadi, A. L. (1989). Antagonistic effect of four fungal isolates to *Ganoderma boninense*, the causal agent of basal stem rot of oil palm. *Biotropia* 3, 41–49.

Diamond, H., and Cooke, B. M. (2003). Preliminary studies on biological control of the Fusarium ear blight complex of wheat. *Crop Prot*. 22 (1), 99–107. doi:10.1016/S0261-2194(02)00117-5

Di Francesco, A., Ugolini, L., D’Aquino, S., Pagnotta, E., and Mari, M. (2017). Biocontrol of *Monilinia laxa* by *Aureobasidium pullulans* strains: insights on competition for nutrients and space. *Int. J. Food Microbiol*. 248, 32–38. doi:10.1016/j.ijfoodmicro.2017.02.007

Diaz, J., Silvar, C., Varela, M. M., Bernal, A., and Merino, F. (2005). *Fusarium* confers protection against several mycelial pathogens of pepper plants. *Plant Pathol*. 54 (6), 773–780. doi: 10.1111/j.1365-3059.2005.01285.x

Dik, A. J., Verhaar, M. A., and Bélanger, R. R. (1998). Comparison of three biological control agents against cucumber powdery mildew (*Sphaerotheca fuliginea*) in semi-commercial-scale glasshouse trials. *Eur. J. Plant Pathol*. 104 (4), 413–423. doi: 10.1023/A:1008025416672

Dimakopoulou, M., Tjamos, S. E., Antoniou, P. P., Pietri, A., Battilani, P., Avramidis, N., et al. (2008). Phyllosphere grapevine yeast *Aureobasidium pullulans* reduces Aspergillus carbonarius(sour rot) incidence in wineproducing vineyards in Greece. *Biol. Control* 46 (2), 158–165. doi: 10.1016/j.biocontrol.2008.04.015

Dominic, M., and Marthamakobe, M. (2017). Biological control of cashew powdery mildew using *Ampelomyces quisqualis* Ces. *J. Biol. Control* 30 (4), 226–235. doi: 10.18311/jbc/2016/15591

Dong, H., Li, W., Zhang, D., and Tang, W. (2003). Differential expression of induced resistance by an aqueous extract of killed *Penicillium chrysogenum* against Verticillium wilt of cotton. Crop Prot. 22 (1), 129–134. doi: 10.1016/S0261-2194(02)00122-9

Dong, H., Zhang, X., Choen, Y., Zhou, Y., Li, W., and Li, Z. (2006). Dry mycelium of *Penicillium chrysogenum* protects cotton plants against wilt diseases and increases yield under field conditions. *Crop Protect*. 25 (4), 324–330. doi:10.1016/j.cropro.2005.05.003

Droby, S., Chalutz, E., Wilson, C. L., and Wisniewski, M. (1989). Characterization of the biocontrol activity of *Debaryomyces hansenii* in the control of Penicillium digitatumon grapefruit. *Can. J. Microbiol*. 35 (8), 794–800. doi:10.1139/m89-132

Droby, S., Hofstein, R., Wilson, C. L., Wisniewski, M., Fridlender, B., Cohen, L., et al. (1993). Pilot testing of *Pichia guilliermondii*: a biocontrol agent of postharvest diseases of citrus fruit. *Biol. Control* 3 (1), 47–52. doi: 10.1006/bcon.1993.1008

Droby, S., Wisniewski, M. E., Cohen, L., Weiss, B., Touitou, D., Eilam, Y., et al. (1997). Influence of CaCl_2_ on *Penicillium digitatum*, grapefruit peel tissue, and biocontrol activity of *Pichia guilliermondii*. *Phytopathology* 87 (3), 310–315. doi: 10.1094/PHYTO.1997.87.3.310

Dubos, B. (1984).“Biocontrol of Botrytis cinerea on grapevines by antagonistic strain of Trichoderma harzianum,”in *Current perspectives in microbiol ecology*. Eds. M. J. Klub and C. A. Reedy (Washington, D.C: Amer. Soc. Microbial.), 26–32.

Durga, P., Sharma, I. M., Sharma, M., and Gaurav, S. (2014). Potential of biological control agents against pink canker (*Corticium salmonicolor* Berk. & Br.) in apple. *J. Biol. Control* 28 (1), 43–47.

Dutta, B. K. (1981). Studies on some fungi isolated from the rhizosphere of tomato plants and the consequent prospect for the control of Verticillium wilt. *Plant Soil* 63 (2), 209–216. doi: 10.1007/BF02374599

Ekefan, E. J., Jama, A., and Gowen, S. R. (2009). Potential of *Trichoderma harzianum* isolates in biocontrol of *Colletotrichum capsici* causing anthracnose of Pepper (*Capsicum* spp.) in Nigeria.J. Appl. Biosci. 20, 1138–1145.

El Ghaouth, A., Wilson, C. L., and Wisniewski, M. (2003). Control of postharvest decay of apple fruit with *Candida saitoana* and induction of defense responses. *Phytopathology*  93 (3), 344–348. doi: 10.1094/PHYTO.2003.93.3.344

El Komy, M.H., Saleh, A.A., Eranthodi, A.,and Molan, Y.Y. (2015). Characterization of novel Trichoderma asperellum isolates to select effective biocontrol agents against tomato Fusarium wilt. *Plant Pathol*. J.31 (1), 50-60. doi: 10.5423/PPJ.OA.09.2014.0087

Elad, Y., Chet, I., and Katan, J. (1980). *Trichoderma harzianum*: A biocontrol agent effective against *Sclerotium rolfsii* and *Rhizoctonia solani*. *Phytopathology* 70 (2), 119–121. doi: 10.1094/Phyto-70-119

Elad, Y., Zvieli, Y., and Chet, I. (1986). Biological control of Macrophomina phaseolina (Tassi) Goid by Trichoderma harzianum. *Crop Protect*. 5 (4), 288–292. doi: 10.1016/0261-2194(86)90065-7

Elad, Y., Kirshner, B., Yehuda, N., and Sztejnberg, A. (1998). Management of powdery mildew and gray mold of cucumber by *Trichoderma harzianum* T39 and *Ampelomyces quisqualis* AQ10. *Bio Control* 43 (2), 241–251. doi.org/10.1023/A:1009919417481

Elad, Y. (1994). Biological control of grape grey mould by *Trichoderma harzianum*. *Crop Protect*. 13 (1), 35–38. doi: 10.1016/0261-2194(94)90133-3

Elad, Y. (2000). Biological control of foliar pathogens by means of *Trichoderma harzianum* and potential modes of action. *Crop Protect*. 19 (8-10), 709–714. doi: 10.1016/S0261-2194(00)00094-6

Elmer, P. A. G., and Reglinski, T. (2006). Biosuppression of *Botrytis cinerea* in grapes. *Plant Pathol*. 55 (2), 155–177. doi: 10.1111/j.1365-3059.2006.01348.x

El-Neshawy, S. M., and Wilson, C. L. (1997). Nisin enhancement of biocontrol of postharvest diseases of apple with *Candida oleophila*. *Postharvest Biol. Technol*. 10 (1), 9–14. doi: 10.1016/S0925-5214(96)00053-1

El-Rafai, I. M., Asswah, S. M., and Awdalla, O. A. (2003). Biocontrol of some tomato disease using some antagonistic microorganisms.Pak. J. Bio. Sci.6 (4), 399–406. doi: 10.3923/pjbs.2003.399.406

El-Sharkaway, M. M., Kamel, S. M., and El-Khateeb, N. M. (2014). Biological control of powdery and downy mildews of cucumber under greenhouse conditions. *Egypt J. Biol. Pest Control* 24 (2), 407.

Escande, A. R., Laich, F. S., and Pedraza, M. V. (2002). Field testing of honey bee dispersed *Trichoderma* spp. to manage sunflower head rot (*Sclerotinia sclerotiorum*). *Plant Pathol*. 51 (3), 346–351. doi.org/10.1046/j.1365-3059.2002.00723.x

Etebarian, H. R., Scott, E. S., and Wicks, T. J. (2000). *Trichoderma harzianum* T39 and *T. virens* DAR 74290 as potential biological control agents for *Phytophthora erythroseptica*. *Eur. J. Plant Pathol*. 106 (4), 329–337. doi.org/10.1023/A:1008736727259

Evueh, G., and Osemwegie, O. (2011). Evaluation Phylloplane Fungi as Biocontrol Agent of Corynespora Leaf Disease of Rubber (*Hevea brasiliensis* Muell. ARG.). *World J. Fungal Plant Biol*. 2 (1), 01–05

Fakhro, A., Andrade-Linares, D. R., von Bargen, S., Bandte, M., Büttner, C., Grosch, R., et al. (2010). Impact of *Piriformospora indica* on tomato growth and on interaction with fungal and viral pathogens. *Mycorrhiza* 20 (3), 191–200. doi: 10.1007/s00572-009-0279-5

Falk, S. P., Gadoury, D. M., Pearson, R. C., and Seem, R. C. (1995a). Partial control of grape powdery mildew by the mycoparasite *Ampelomyces quisqualis*. *Plant Dis*.79 (5), 483–490. doi: 10.1094/PD-79-0483

Falk, S. P., Gadoury, D. M., CortesI, P., Pearson, R. C., and Seem, R. C. (1995b). Parasitism of *Uncinula necatorascomata* by the mycoparasite *Ampelomyces quisqualis*. *Phytopathology*  85, 794–800. doi: 10.1094/Phyto-85-794

Fan, Q., and Tian, S. (2001). Postharvest biological control of grey mold and blue mold on apple by *Cryptococcus albidus* (Saito) Skinner. *Postharvest Biol. Technol*. 21 (3), 341–350. doi: 10.1016/S0925-5214(00)00182-4

Fang, J. G., and Tsao, P. H. (1995). Efficacy of *Penicillium funiculosum* as a biological control agent against Phytophthora root rots of azalea and citrus. *Phytopathology* 85 (8), 871–878. doi: 10.1094/Phyto-85-871

Foroutan, A. (2013). Evaluation of *Trichoderma* isolates for biological control of wheat Fusarium foot and root rot. *Rom. Agr. Res*.30, 35–44.

Fowler, S. R., Jaspers, M. V., Walter, M., and Stewart, A. (1999). “ Suppression of overwintering *Botrytis cinerea* inoculum on grape rachii using antagonistic fungi,” in Proceedings of the New zealand plant protection conference (New Zealand Plant Protection Society, New Zealand), 141–147

França, S. C., Spiessens, K., Pollet, S., Debode, J., De Rooster, L., Callens, D., et al. (2013). Population dynamics of *Verticillium* species in cauliflower fields: influence of crop rotation, debris removal and ryegrass incorporation. *Crop Protect*. 54, 134–141. doi: 10.1016/j.cropro.2013.08.008

Freeman, S., Minz, D., Kolesnik, I., Barbul, O., Zveibil, A., Maymon, M., et al. (2004). *Trichoderma* biocontrol of *Colletotrichum acutatum* and *Botrytis cinerea* and survival in strawberry. *Eur. J. Plant Pathol*. 110 (4), 361–370. doi: 10.1023/B:EJPP.0000021057.93305.d9

Gafni, A., Calderon, C. E., Harris, R., Buxdorf, K., Dafa-Berger, A., ZeilingerReichert, E., et al. (2015). Biological control of the cucurbit powdery mildew pathogen *Podosphaera xanthii* by means of the epiphytic fungus *Pseudozyma aphidis* and parasitism as a mode of action. Front. *Plant Sci*. 6, 132. doi:10.3389/fpls.2015.00132

Gamagae, S. U., Sivakumar, D., Wijeratnam, R. W., and Wijesundera, R. L. C. (2003). Use of sodium bicarbonate and *Candida oleophila* to control anthracnose in papaya during storage. *Crop Prot*. 22 (5), 775–779. doi:10.1016/S0261-2194(03)00046-2

Garmendia, I., Goicoechea, N., and Aguirreolea, J. (2004). Antioxidant Metabolism in Asymptomatic Leaves of Verticillium-infected Pepper Associated with an Arbuscular Mycorrhizal Fungus. *J. Phytopathol*. 152 (11-12), 593–599. doi: 10.1111/j.1439-0434.2004.00901.x

Gauthier, N. W., Maruthachalam, K., Subbarao, K. V., Brown, M., Xiao, Y., Robertson, C. L., et al. (2014). Mycoparasitism of *Phakopsora pachyrhizi*, the soybean rust pathogen, by *Simplicillium lanosoniveum*. *Biol. Control* 76, 87–94.doi: 10.1016/j.biocontrol.2014.05.008

Gees, R., and Coffey, M. 1. (1989). Evaluation of a strain of *Myrothecium roridum* as a potential biocontrol agent against *Phytophthora cinnamomi*. *Phytopathology* 79 (10), 1079–1084. doi: 10.1094/Phyto-79-1079

Gizi, D., Stringlis, I. A., Tjamos, S. E., and Paplomatas, E. J. (2011). Seedling vaccination by stem injecting a conidial suspension of F2, a non-pathogenic *Fusarium oxysporum* strain, suppresses Verticillium wilt of eggplant. *Biol. Control* 58 (3), 387–392. doi: 10.1016/j.biocontrol.2011.06.009

Gnanamangai, B. M., and Ponmurugan, P. (2012). Evaluation of various fungicides and microbial based biocontrol agents against bird’s eye spot disease of tea plants. *Crop Protect*. 32, 111–118. doi: 10.1016/j.cropro.2011.10.001

Goates, B. J., and Mercier, J. (2011). Control of common bunt of wheat underfield conditions with the biofumigant fungus *Muscodor albus*. *Eur. J. Plant Pathol*. 131 (3), 403–407. doi: 10.1007/s10658-011-9817-z

Goodman, D. M., and Burpee, L. L. (1991). Biological control of dollar spot disease of creeping bentgrass. *Phytopathology* 81 (11), 1438–1446. doi: 10.1094/Phyto-81-1438

Gowdu, B. J., and Balasubramanian, R. (1993). Biocontrol potential of rust of groundnut by *Acremonium obclavatum*. *Can. J. Bot*. 71 (4), 639–643. doi:10.1139/b93-073

Gracia-Garza, J. A., Reeleder, R. D., and Paulitz, T. C. (1997). Degradation of sclerotia of *Sclerotinia sclerotiorum* by fungus gnats (*Bradysia coprophila*) and the biocontrol fungi *Trichoderma* spp. *Soil Biol. Biochem*. 29 (2), 123–129. doi:10.1016/S0038-0717(96)00299-4

Grondona, I., Hermosa, R., Tejada, M., Gomis, M. D., Mateos, P. F., Bridge, P. D., et al. (1997). Physiological and biochemical characterization ofTrichoderma harzianum, a biological control agent against soilborne fungal plant pathogens. *Appl. Environ. Microbiol*. 63 (8), 3189–3198. doi: 10.1128/AEM.63.8.3189-3198.1997

Guijarro, B., Larena, I., Melgarejo, P., and De Cal, A. (2006). Effect of drying on conidial viability of *Penicillium frequentans*, a biological control agent against peach brown rot disease caused by *Monilinia* spp. *Biocontrol Sci. Technol*.16(3), 257–269. doi: 10.1080/09583150500335897

Guijarro, B., Melgarejo, P., Torres, R., Lamarca, N., Usall, J., and De Cal, A. (2007). Effects of different biological formulations of *Penicillium frequentans* on brown rot of peaches. *Biol. Control* 42 (1), 86–96. doi: 10.1016/j.biocontrol.2007.03.014

Guzzon, R., Franciosi, E., and Larcher, R. (2014). A new resource from traditional wines: characterisation of the microbiota of “Vino Santo” grapes as a biocontrol agent against *Botrytis cinerea*. *Eur. Food Res. Technol*. 239 (1), 117–126. doi: 10.1007/s00217-014-2195-y

Gveroska, B., and Ziberoski, J. (2012).*Trichoderma harzianum* as a biocontrol agent against *Alternaria alternata* on tobacco. *ATI-Applied Technol. Innov*. 7 (2), 67–76. doi: 10.15208/ati.2012.9

Hanada, R. E., Pomella, A. W., Soberanis, W., Loguercio, L. L., and Pereira, J. O. (2009). Biocontrol potential of *Trichoderma martiale* against the black-pod disease (*Phytophthora palmivora*) of cacao. *Biol. Control* 50 (2), 143–149. doi:10.1016/j.biocontrol.2009.04.005

Hanada, R. E., Pomella, A. W. V., Costa, H. S., Bezerra, J. L., Loguercio, L. L., and Pereira, J. O. (2010). Endophytic fungal diversity in *Theobroma cacao* (cacao) and *T. grandiflorum* (cupuaçu) trees and their potential for growth promotion and biocontrol of black-pod disease. *Fungal Biol*. 114 (11-12), 901–910. doi: 10.1016/j.funbio.2010.08.006

Hanson, L. E. (2000). Reduction of Verticillium wilt symptoms in cotton following seed treatment with *Trichoderma virens*. *J. Cotton Sci*. 4 (4), 224–231.

Harish, S., Saravanakumar, D., Radjacommare, R., Ebenezar, E. G., and Seetharaman, K. (2008). Use of plant extracts and biocontrol agents for the management of brown spot disease in rice. *Biol. Control* 53 (3), 555. doi: 10.1007/s10526-007-9098-9

Harrison, Y. A., and Stewart, A. (1988). Selection of fungal antagonists for biological control of onion white rot in New Zealand.New Z. J. Exp. Agric. 16 (3), 249–256. doi: 10.1080/03015521.1988.10425647

Haugaard, H., Lyngs Jørgensen, H. J., Lyngkjær, M. F., Smedegaard-Petersen, V., and Collinge, D. B. (2001). Control of *Blumeria graminis* f. sp. *hordei* by treatment with mycelial extracts from cultured fungi. *Plant Pathol*. 50 (5), 552–560. doi.org/10.1046/j.1365-3059.2001.00595.x

Hazarika, D. K., Phookan, A. K., Saikia, G. K., Borthakur, B. K., and Sarma, D. (2000). Management of charcoal stump rot of tea with bio control agents. *J. Plantation Crops* 28 (2), 149–153.

Highley, T. L., and Ricard, J. (1988). Antagonism of *Trichoderma* spp. and *Gliocladium virens* againstwood decayfungi. *Mater. und Org*. 23(3),157-169.

Hijwegen, T. (1992). Biological control of cucumber powdery mildew with *Tilletiopsis minorunder* greenhouse conditions. *Netherlands J. Plant Pathol*. 98 (4), 221–225. doi: 10.1007/BF02000089

Holmes, K. A., Schroers, H. J., Thomas, S. E., Evans, H. C., and Samuels, G. J. (2004). Taxonomy and biocontrol potential of a new species of *Trichoderma* from the Amazon basin of South America. *Mycol. Prog*. 3 (3), 199–210. doi:10.1007/s11557-006-0090-z

Huang, H. C., Bremer, E., Hynes, R. K., and Erickson, R. S. (2000). Foliar application of fungal biocontrol agents for the control of white mold of dry bean caused by *Sclerotinia sclerotiorum*. *Biol. Control* 18 (3), 270–276. doi:10.1006/bcon.2000.0829

Hung, P. M., Wattanachai, P., Kasem, S., and Poaim, S. (2015). Biological Control of *Phytophthora palmivora* Causing Root Rot of Pomelo Using *Chaetomium* spp. Mycobiology 43 (1), 63–70. doi: 10.5941/MYCO.2015.43.1.63

Hussain, S., Ghaffar, A., and Aslam, M. (1990). Biological control of *Macrophomina phaseolina* charcoal rot of sunflower and mung bean. *J. Phytopathol*. 130 (2), 157–160. doi: 10.1111/j.1439-0434.1990.tb01163.x

Hwang, S. F., Chang, K. F., and Chakravarty, P. (1992). Effects of vesicular-arbuscular mycorrhizal fungi on the development of Verticillium and Fusarium wilts of alfalfa. *Plant Dis*. 76 (3), 239–243. doi: 10.1094/PD-76-0239

Ippolito, A., El Ghaouth, A., Wilson, C. L., and Wisniewski, M. (2000). Control of postharvest decay of apple fruit by *Aureobasidium pullulans* and induction of defense responses. *Postharvest Biol*. *Technol*.19 (3), 265–272. doi: 10.1016/S0925-5214(00)00104-6

Jabnoun-Khiareddine, H., Daami-Remadi, M., Ayed, F., and El-Mahjoub, M. (2009). Biological control of tomato Verticillium wilt by using indigenous *Trichoderma* spp. *Afr. J. Plant Sci. Biotechnol*. 3 (1), 26–36.

Jackson, D., Skillman, J., and Vandermeer, J. (2012). Indirect biological control of the coffee leaf rust, *Hemileia vastatrix*, by the entomogenous fungus *Lecanicillium lecaniiin* a complex coffee agroecosystem. *Biol. Control* 61 (1), 89–97. doi: 10.1016/j.biocontrol.2012.01.004

Jagtap, G. P., Mali, A. K., and Dey, U. (2013). Bioefficacy of fungicides, bio-control agents and botanicals against leaf spot of turmeric incited by *Colletortricum capsici*. *Afr. J. Microbiol. Res*.7 (18), 1865–1873. doi: 10.5897/AJMR12.2252

Janisiewicz, W. J. (1988). Biocontrol of postharvest diseases of apples with antagonist mixtures. *Phytopathology* 78, 194–198. doi: 10.1094/Phyto-78-194

Jayasuriya, K. E., and Thennakoon, B. I. (2007). Biological control of *Rigidoporus microporus*, the cause of white root disease in rubber. *Cey. J. Sci. (Bio. Sci)*. 36 (1), 9–16.

Jima, T. A. (2013). Postharvest biological control of Fusarium dry-rot disease in potato tubers using *Clonostachys rosea* strain IK726 (Uppsala: Swedish University of Agricultural Sciences (SLU), Department of Forest Mycology and Plant Pathology), 42 p.

John, R. P., Tyagi, R. D., Prévost, D., Brar, S. K., Pouleur, S., and Surampalli, R. Y. (2010). Mycoparasitic *Trichoderma viride* as a biocontrol agent against *Fusarium oxysporum* f. sp. *adzuki* and *Pythium arrhenomanes* and as a growth promoter of soybean. *Crop Prot*. 29 (12), 1452–1459. doi:10.1016/j.cropro.2010.08.004

Jollands, P. (1983). Laboratory investigations on fungicides and biological agents to control three diseases of rubber and oil palm and their potential applications. *Int. J. Pest Manage*. 29 (1), 33–38. doi: 10.1080/09670878309370766

Jones, E. E., and Whipps, J. M. (2002). Effect of inoculum rates and sources of *Coniothyrium minitans* oncontrol of *Sclerotinia sclerotiorum* disease in glasshouse lettuce. *Eur. J. Plant Pathol*. 108 (6), 527–538. doi: 10.1023/A:1019940820230

Kaewchai, S., and Soytong, K. (2010). Application of biofungicides against *Rigidoporus microporus* causing white root disease of rubber trees. *J. Agric. Technol*. 6 (2), 349–363.

Kannangara, S., Dharmarathna, R.M.G.C.S., and Jayarathna, D. L. (2017). Isolation, identification and characterization of *Trichoderma* species as a potential biocontrol agent against *Ceratocystis paradoxa*. *J. Agric. Sciences–Sri Lanka*. 12 (1), 51–62. doi: 10.4038/jas.v12i1.8206

Karabulut, O. A., Smilanick, J. L., Gabler, F. M., Mansour, M., and Droby, S. (2003). Near-harvest applications of *Metschnikowia fructicola*, ethanol, and sodium bicarbonate to control postharvest diseases of grape in central California. *Plant Dis*. 87 (11), 1384–1389. doi: 10.1094/PDIS.2003.87.11.1384

Karabulut, O. A., and Baykal, N. (2004). Integrated control of postharvest diseases of peaches with a yeast antagonist, hot water and modified atmosphere packaging. *Crop Prot*. 23 (5), 431–435. doi: 10.1016/j.cropro.2003.09.012

Karabulut, O. A., Arslan, U., Ilhan, K., and Kuruoglu, G. (2005). Integrated control of postharvest diseases of sweet cherry with yeast antagonists and sodium bicarbonate applications within a hydrocooler. *Postharvest Biol. Technol*. 37 (2), 135–141. doi: 10.1016/j.postharvbio.2005.03.003

Karthikeyan, V., Brindha, S., Annadurai, B., and Gangwar, S. K. (2015). Biological control of *Macrophomina phaseolina* (Tassi) Goid root rot in Vigna mungo (Black gram) with *Trichoderma* spp. *Int. J. Adv. Biol. Res*. 5 (2), 118–127.

Kefialew, Y., and Ayalew, A. (2008). Postharvest biological control of anthracnose (*Colletotrichum gloeosporioides*) on mango (*Mangifera indica*). *Postharvest Biol. Technol.* 50 (1), 8–11. doi: 10.1016/j.postharvbio.2008.03.007

Kessel, G. J. T., De Haas, B. H., Lombaers-Van der Plas, C. H., Van den Ende, J. E., Pennock-Vos, M. G., Van der Werf, W., et al. (2001). Comparative analysis of the role of substrate specificity in biological control of *Botrytis elliptica* in lily and *B. cinerea* in cyclamen with *Ulocladium atrum*. *Eur. J. Plant Pathol*. 107(3), 273–284. doi.org/10.1023/A:1011273613406

Kexiang, G., Xiaoguang, L., Yonghong, L., Tianbo, Z., and Shuliang, W. (2002). Potential of *Trichoderma harzianum* and *T. atroviride* to control *Botryosphaeria berengeriana* f. sp. piricola, the cause of apple ring rot*. J. Phytopathol*. 150 (4-5), 271–27. doi.org/10.1046/j.1439-0434.2002.00754.x

Kheireddine, A., Essghaier, B., Hedi, A., Dhieb, C., and Zouaoui, N. S. (2018). New epiphytic yeasts able to reduce grey mold disease on apples. *Plant Prot. Sci*. 54 (4), 248–257. doi.org/10.17221/103/2017-PPS

Khokhar, I., Haider, M. S., Mukhtar, I., and Mushtaq, S. (2013). Biological control of *Aspergillus niger*, the cause of Black-rot disease of *Allium cepa* L. (onion), by *Penicillium* species. *J. Agrobiol*. 29 (1), 23–28. doi: 10.2478/v10146-012-0003-5

Kim, J. J., Goettel, M. S., and Gillespie, D. R. (2007a). Potential of *Lecanicillium* species for dual microbial control of aphids and the cucumber powdery mildew fungus, *Sphaerotheca fuliginea*. *Biol. Control* 40 (3), 327–332. doi: 10.1016/j.biocontrol.2006.12.002

Kim, H. Y., Choi, G. J., Lee, H. B., Lee, S. W., Lim, H. K., Jang, K. S., et al. (2007b). Some fungal endophytes from vegetable crops and their anti-oomycete activities against tomato late blight. *Lett. Appl. Microbiol*. 44 (3), 332–337. doi: 10.1111/j.1472-765X.2006.02093.x

Kiss, L., Russell, J. C., Szentiványi, O., Xu, X., and Jeffries, P. (2004). Biology and biocontrol potential of *Ampelomyces mycoparasites*, natural antagonists of powdery mildew fungi. *Biocontrol Sci. Technol*. 14 (7), 635–651. doi: 10.1080/09583150410001683600

Knudsen, I. M., and Skou, J. P. (1993). The effectivity of *Tilletiopsis albescens* in biocontrol of powdery mildew. *Ann. Appl. Biol*. 123 (1), 173–185. doi: 10.1111/j.1744-7348.1993.tb04084.x

Knudsen, I. M., Hockenhull, J., and Jensen, D. F. (1995). Biocontrol of seedling diseases of barley and wheat caused by *Fusarium culmorum* and *Bipolaris sorokiniana*: effects of selected fungal antagonists on growth and yield components. *Plant Pathol*. 44 (3), 467–477. doi: 10.1111/j.1365-3059.1995.tb01669.x

Köhl, J. (2004). Biological control of *Botrytis* spp. by *Ulocladium atrum* through competitive colonisation of necrotic plant tissues (Wageningen, The Netherlands: Plant Research International. B.V.). pp 1-213.

Kotze, C.,Van Niekerk, J., Mostert, L., Halleen, F., and Fourie, P. (2011). Evaluation of biocontrol agents for grapevine pruning wound protection against trunk pathogen infection. *Phytopathol. Mediterranea*. 50, S247–S263

Krauss, U., and Soberanis, W. (2001). Biocontrol of cocoa pod diseases with mycoparasite mixtures. *Biol. Control*. 22 (2), 149–158. doi: 10.1006/bcon.2001.0956

Kumar, S., and Jha, D. K. (2002).*Trichothecium roseum*: a potential agent for the biological control of soybean rust. *Indian Phytopathol*. 55 (2), 232–234.

Kumari, M., and Singh, M. (2017). Management of collar rot disease of groundnut (*Arachis hypogaea* L.) Caused by *Aspergillus niger* through bio-agents. *Int. J. Chem. Stud*. 5 (4), 73–76.

Kusari, P., Kusari, S., Spiteller, M., and Kayser, O. (2013). Endophytic fungi harbored in *Cannabis sativa* L.: diversity and potential as biocontrol agents against host plant-specific phytopathogens. *Fungal Divers*. 60 (1), 137–151. doi: 10.1007/s13225-012-0216-3

La, N. H., Thiep, N. V., and Soytong, K. (2016). Research to Produce Biological
Products of *Chaetonium* to Control Fungal Diseases on Tea, Coffee and
Rubber. *Int. J. Agric. Technol*. 12 (6), 993–1004.

Lahlali, R., Serrhini, M. N., and Jijakli, H. (2004). Efficacy assessment of *Candida
oleophila* (strain O) and *Pichia anomala* (strain K) against major postharvest
diseases of citrus fruits in Morocco. *Commun. Appl. Biol. Sci*. *Ghent University* 69 (4), 601–609.

Lal, R. J., Sinha, O. K., Bhatnagar, S., Lal, S., and Awasthi, S. K. (2009). Biological control of sugarcane smut (*Sporisorium scitamineum*) through botanicals and *Trichoderma viride*. *Sugar Tech*. 11 (4), 381–386. doi: 10.1007/s12355-009-0065-x

Landum, M. C., do Rosário Félix, M., Alho, J., Garcia, R., Cabrita, M. J., Rei, F.,
et al. (2016). Antagonistic activity of fungi of *Olea europaea* L. against
*Colletotrichum acutatum. Microbiol. Res.* 183, 100–108. doi: 10.1016/
j.micres.2015.12.001

Larena, I., Sabuquillo, P., Melgarejo, P., and De Cal, A. (2003). Biocontrol of Fusarium and Verticillium wilt of tomato by *Penicillium oxalicum* under greenhouse and field conditions. *J. Phytopathol*. 151 (9), 507–512. doi:10.1046/j.1439-0434.2003.00762.x

Larkin, R. P., and Fravel, D. R. (1998). Efficacy of various fungal and bacterial biocontrol organisms for control of Fusarium wilt of tomato. *Plant Dis*. 82 (9), 1022–1028. doi: 10.1094/PDIS.1998.82.9.1022

Larran, S., Simon, M. R., Moreno, M. V., Siurana, M. S., and Perelló, A. (2016). Endophytes from wheat as biocontrol agents against tan spot disease. *Biol. Control*. 92, 17–23. doi: 10.1016/j.biocontrol.2015.09.002

Lassois, L., de Bellaire, L. D. L., and Jijakli, M. H. (2008). Biological control of
crown rot of bananas with *Pichia anomala* strain K and *Candida oleophila*
strain O. *Biol. Control* 45 (3), 410–418. doi: 10.1016/j.biocontrol.2008.01.013

Latunde-Dada, A. O. (1993). Biological control of southern blight disease of
tomato caused by *Sclerotium rolfsii* with simplified mycelial formulations of
*Trichoderma koningii*. *Plant Pathol*. 42 (4), 522–529. doi: 10.1111/j.1365-
3059.1993.tb01532.x

Lee, J. T., Bae, D. W., Park, S. H., Shim, C. K., Kwak, Y. S., and Kim, H. K. (2001).
Occurrence and biological control of postharvest decay in onion caused by
fungi. *Plant Pathol. J.* 17 (3), 141–148.

Lee, J. (2018). Bio-control of the soil-borne pathogen *Rhizoctonia solani* of radish (*Raphanus sativus* L.) by *Trichoderma* species (Doctoral dissertation, Lincoln University, New Zealand).

Li, B. C., and Sutton, J. C. (1995). Evaluation of leaf-associated microorganisms for
biocontrol of tan spot in wheat foliage. *Fitopatol. Brasileira*. 20 (4), 545–552.

Li, Z. F., Wang, L. F., Feng, Z. L., Zhao, L. H., Shi, Y. Q., and Zhu, H. Q. (2014).
Diversity of endophytic fungi from different Verticillium-wilt-resistant
*Gossypium hirsutum* and evaluation of antifungal activity against
*Verticillium dahliae* *in vitro*. *J. Microbiol. Biotechnol*. 24 (9), 1149–1161. doi:
10.4014/jmb.1402.02035

Lim, T. K., and Rohrbach, K. G. (1980). Role of *Penicillium funiculosum* strains in
the development of pineapple fruit diseases. *Phytopathology* 70 (7), 663–665.
doi: 10.1094/Phyto-70-663

Liu, S. J., and Wen, C. J. (2005). Screening for *Trichoderma* spp. and *Gliocladium*
spp. Against Rapesee Sclerotinia Stem Rot and Primary Study of Biocontrol.
*Sichuan Nongye Daxue Xuebao*. 23 (1), 33-38.

Liu, X., Fang, W., Liu, L., Yu, T., Lou, B., and Zheng, X. (2010a). Biological control
of postharvest sour rot of citrus by two antagonistic yeasts. *Lett. Appl.
Microbiol.* 51 (1), 30–35. doi: 10.1111/j.1472-765X.2010.02851.x

Liu, H. M., Guo, J. H., Liu, P., Cheng, Y. J., Wang, B. Q., Long, C. A., et al. (2010b).
Inhibitory activity of tea polyphenol and *Candida ernobii* against *Diplodia
natalensis* infections. *J. Appl. Microbiol.* 108 (3), 1066–1072. doi: 10.1111/
j.1365-2672.2009.04511.x

Liu, J., Wisniewski, M., Droby, S., Vero, S., Tian, S., and Hershkovitz, V. (2011a).
Glycine betaine improves oxidative stress tolerance and biocontrol efficacy of
the antagonistic yeast *Cystofilobasidium infirmominiatum. Int. J. Food
Microbiol.* 146 (1), 76–83. doi: 10.1016/j.ijfoodmicro.2011.02.007

Liu, J., Wisniewski, M., Droby, S., Tian, S., Hershkovitz, V., and Tworkoski, T.
(2011b). Effect of heat shock treatment on stress tolerance and biocontrol
efficacy of *Metschnikowia fructicola. FEMS Microbiol. Ecol.* 76 (1), 145–155.
doi: 10.1111/j.1574-6941.2010.01037.x

Liu, R. J. (1995). Effect of vesicular-arbuscular mycorrhizal fungi on Verticillium
wilt of cotton. *Mycorrhiza* 5 (4), 293–297. doi: 10.1007/BF00204965

Long, C. A., Deng, B. X., and Deng, X. X. (2006). Pilot testing of *Kloeckera
apiculata* for the biological control of postharvest diseases of citrus. *Ann.
Microbiol*. 56 (1), 13 doi.org/10.1007/BF03174963

Lopatecki, L. E., and Peters, W. (1972). A rot of pears in cold storage caused by
*Mucor piriformis. Can. J. Plant Sci*. 52 (6), 875–879. doi: 10.4141/cjps72-151

Ma, Y., Chang, Z. Z., Zhao, J. T., and Zhou, M. G. (2008). Antifungal activity of
*Penicillium striatisporum* Pst10 and its biocontrol effect on Phytophthora root
rot of chilli pepper. *Biol. Control* 44 (1), 24–31. doi: 10.1016/
j.biocontrol.2007.10.005

Madi, L., Katan, T., Katan, J., and Henis, Y. (1997). Biological control of *Sclerotium
rolfsii* and *Verticillium dahliae* by *Talaromyces flavus* is mediated by different
mechanisms. *Phytopathology* 87 (10), 1054–1060. doi: 10.1094/
PHYTO.1997.87.10.1054

Madrigal, C., Pascual, S., and Melgarejo, P. (1994). Biological control of peach twig
blight (*Monilinia laxa*) with *Epicoccum nigrum*. *Plant Pathol*. 43 (3), 554–561.
doi: 10.1111/j.1365-3059.1994.tb01590.x

Maghazy, S. M. N., Abdelzaher, H. M. A., Haridy, M. S., and Moustafa, S. M. N.
(2008). Biological control of damping-off disease of *Trifolium alexandrinum* L.
caused by *Pythium spinosum* Sawada var. spinosum using some soil fungi.
*Arch. Phytopathol. Plant Prot*. 41 (6), 431–450 doi.org/10.1080/03235400600813680

Malandraki, I., Tjamos, S. E., Pantelides, I. S., and Paplomatas, E. J. (2008).
Thermal inactivation of compost suppressiveness implicates possible biological
factors in disease management. *Biol. Control* 44 (2), 180–187. doi: 10.1016/
j.biocontrol.2007.10.006

Mandal, G., Singh, D., and Sharma, R. R. (2007). Effect of hot water treatment and
biocontrol agent (*Debaryomyces hansenii*) on shelf-life of peach. *Indian J.
Horticult*. 64 (1), 25–28 doi: 10.1094/Phyto-71-1257.

Mao, W., Lewis, J. A., Hebbar, P. K., and Lumsden, R. D. (1997). Seed treatment
with a fungal or a bacterial antagonist for reducing corn damping-off caused by
species of *Pythium* and *Fusarium. Plant Dis.* 81 (5), 450–454. doi: 10.1094/
PDIS.1997.81.5.450

Mari, M., Martini, C., Guidarelli, M., and Neri, F. (2012). Postharvest biocontrol of
*Monilinia laxa, Monilinia fructicola* and *Monilinia fructigena* on stone fruit by
two *Aureobasidium pullulans* strains. *Biol. Control* 60 (2), 132–140. doi:
10.1016/j.biocontrol.2011.10.013

Marois, J. J., Mitchell, D. J., and Sonoda, R. M. (1981). Biological control of
Fusarium crown and root rot of tomato under field conditions. *Phytopathology*71, 1257–1260 doi: 10.1094/Phyto-71-1257.

Marois, J. J., Johnston, S. A., Dunn, M. T., and Papavizas, G. C. (1982). Biological
control of Verticillium wilt of eggplant in the field. *Plant Dis.* 66 (12), 1166–
1168. doi: 10.1094/PD-66-1166

Mathivanan, N., and Murugesan, K. (2000). *Fusarium chlamydosporum*, a potent biocontrol agent to groundnut rust, *Puccinia arachidis*/*Fusarium chlamydosporum*, ein potenter Organismus für die biologische Bekämpfung von Rost (Puccinia arachidis) an Erdnuss. *Z. für Pflanzenkrankheiten und Pflanzenschutz/Journal Plant Dis. Prot*., 107 (3), 225–234.

Matsubara, Y. I., Tamura, H., and Harada, T. (1995). Growth enhancement and
Verticillium wilt control by vesicular-arbuscular mycorrhizal fungus
inoculation in eggplant. *J. Japanese Soc. Hortic. Sci.* 64 (3), 555–561. doi:
10.2503/jjshs.64.555

Matta, A., and Garibaldi, A. (1977). Control of Verticillium wilt of tomato by preinoculation with avirulent fungi. *Netherlands J. Plant Pathol*. 83 (1), 457–462. doi: 10.1007/BF03041463

McLaren, D. L., Huang, H. C., Kozub, G. C., and Rimmer, S. R. (1994). Biological
control of *sclerotinia wilt* of sunflower by *Talaromyces flavus* and
*Coniothyrium minitans. Plant Dis*. 78, 231–235. doi: 10.1094/PD-78-0231

McLaughlin, R. J., Wisniewski, M. E., Wilson, C. L., and Chalutz, E. (1990). Effect of inoculum concentration and salt solutions on biological control of postharvest diseases of apple with *Candida* sp. *Phytopathology* 80 (5), 456–461. doi: 10.1094/Phyto-80-456

Meena, P. D., Meena, R. L., Chattopadhyay, C., and Kumar, A. (2004).
Identification of critical stage for disease development and biocontrol of
Alternaria blight of Indian mustard (*Brassica juncea*). *J. Phytopathol*. 152
(4), 204–209. doi: 10.1111/j.1439-0434.2004.00828.x

Mejıa, L. C., Rojas, E. I., Maynard, Z., Van Bael, S., Arnold, A. E., Hebbar, P., et al. ́
(2008). Endophytic fungi as biocontrol agents of *Theobroma* *cacao* pathogens.
*Biol. Control* 46 (1), 4–14. doi: 10.1016/j.biocontrol.2008.01.012

Melgarejo, P., Carrillo, R., and Sagasta, E. M. (1986). Potential for biological
control of *Monilinia laxa* in peach twigs. *Crop Prot.* 5 (6), 422–426. doi:
10.1016/0261-2194(86)90076-1

Melouk, H. A., and Horner, C. E. (1975). Cross protection in mints by *Verticillium
nigrescens* against *V. dahliae. Phytopathology* 65, 767–769. doi: 10.1094/Phyto-
65-767

Menendez, A. B., and Godeas, A. (1998). Biological control of *Sclerotinia
sclerotiorum* attacking soybean plants. Degradation of the cell walls of this
pathogen by *Trichoderma harzianum* (BAFC 742). *Mycopathologia* 142 (3),
153–160 doi.org/10.1023/A:1006910707804

Metcalf, D. A., and Wilson, C. R. (2001). The process of antagonism of *Sclerotium
cepivorum* in white rot affected onion roots by. *Trichoderma koningii. Plant
Pathol*. 50 (2), 249–257. doi: 10.1046/j.1365-3059.2001.00549.x

Metcalf, D. A. (1997). Biological control of *Sclerotium cepivorum* Berk. using
*Trichoderma koningii* Oudem (Hobart, Australia: University of Tasmania, PhD
thesis).

Michereff, S. J., Silva, J. B., Silveira, N. S. S., Pedrosa, R. A., Mariano, R. L. R.,
Tavares, L. A., et al. (1997). Postharvest biocontrol of Lasiodiplodia rot of
mango fruits by saprophytic yeasts. *Arquivos Biol. e Tecnol.* 40 (1), 29–37.

Mirmajlessi, S. M., MÄND, M., Najdabbasi, N., Larena, I., and Loit, E. (2016). Screening of native *Trichoderma harzianum* isolates for their ability to control Verticillium wilt of strawberry.Seyed mahyar mirmajlessi.136. doi: 10.13080/z-a.2016.103.051

Mishra, P. K., Mukhopadhyay, A. N., and Fox, R. T. V. (2000). Integrated and
biological control of gladiolus corm rot and wilt caused by *Fusarium
oxysporum* f. sp. *gladioli. Ann. Appl. Biol.* 137 (3), 361–364. doi: 10.1111/
j.1744-7348.2000.tb00078.x

Mishra, P., Kumar, R., Singh, V., and Singh, G. (2009). Integration of organic
amendments and antagonists for the management of sheath blight in aromatic
rice. *J. Biol. Control* 23 (3), 305–309.

Mishra, B. K., Mishra, R. K., Mishra, R. C., Tiwari, A. K., Yadav, R. S., and Dikshit,
A. (2011). Biocontrol efficacy of *Trichoderma viride* isolates against fungal
plant pathogens causing disease in *Vigna radiata* *L. Arch. Appl. Sci. Res.* 3 (2),
361–369.

Mohamed, H. A. L. A., and Haggag, W. M. (2006). Biocontrol potential of salinity
tolerant mutants of *Trichoderma harzianum* against. *Fusarium oxysporum*.
*Braz. J. Microbiol*. 37 (2), 181–191.

Mohamed, H., and Saad, A. (2009). The biocontrol of postharvest disease (*Botryodiplodia theobromae*) of guava (*Psidium guajava* L.) by the application of yeast strains. *Postharvest Biol. Technol*. 53 (3), 123–130. doi: 10.1016/j.postharvbio.2009.04.001

Mónaco, C., Dal Bello, G., Rollán, M. C., Ronco, L., Lampugnani, G., Arteta, N.,
et al. (2009). Biological control of *Botrytis cinerea* on tomato using naturally
occurring fungal antagonists. *Arch. Phytopathol. Plant Prot.* 42 (8), 729–737.
doi: 10.1080/03235400701390646

Morales, H., Sanchis, V., Usall, J., Ramos, A. J., and Marın, S. (2008). Effect of ́
biocontrol agents *Candida sake* and *Pantoea agglomerans* on *Penicillium
expansum* growth and patulin accumulation in apples. *Int. J. Food Microbiol.*122 (1-2), 61–67. doi: 10.1016/j.ijfoodmicro.2007.11.056

Moreno, L. S., and Paningbatan, R. A. (1995). Biological control of mango stemend rot caused by *Diplodia natalensis* with. *Trichoderma Viride. Philippine
Phytopathol.* 31, 103–116.

Morita, S., Azuma, M., Aoba, T., Satou, H., Narisawa, K., and Hashiba, T. (2003).
Induced systemic resistance of Chinese cabbage to bacterial leaf spot and Alternaria leaf spot by the root endophytic fungus, *Heteroconium chaetospira.
J. Gen. Plant Pathol*. 69 (1), 71–75. doi: 10.1007/s10327-002-0005-z

Mortuza, M. G., and Ilag, L. L. (1999). Potential for biocontrol of *Lasiodiplodia
theobromae* (Pat.) Griff. & Maubl. in banana fruits by *Trichoderma* species.
*Biol. Control* 15 (3), 235–240

Mosquera-Espinosa, A. T., Bayman, P., Prado, G. A., Gómez-Carabalı, A., and ́
Otero, J. T. (2013). The double life of *Ceratobasidium*: orchid mycorrhizal
fungi and their potential for biocontrol of *Rhizoctonia solani* sheath blight of
rice. *Mycologia* 105 (1), 141–150. doi: 10.3852/12-079

Munkvold, G. P., and Marois, J. J. (1993). Efficacy of natural epiphytes and
colonizers of grapevine pruning wounds for biological control of Eutypa
dieback. *Phytopathology* 83 (6), 624–629. doi: 10.1094/Phyto-83-624

Nagtzaam, M. P. M., Bollen, G. J., and Termorshuizen, A. J. (1998). Efficacy of *Talaromyces flavus* alone or in combination with other antagonists in controlling *Verticillium dahlia* in growth chamber experiments. *J. Phytopathol*. 146 (4), 165–173. doi: 10.1111/j.1439-0434.1998.tb04674.x

Naguleswaran, V., Pakeerathan, K., and Mikunthan, G. (2014). Biological control:
a promising tool for bulb-rot and leaf twisting fungal diseases in red onion
(*Allium cepa* L.) in Jaffna district. *World Appl. Sci. J*. 31 (6), 1090–1095

Nakayama, T., and Sayama, M. (2013). “Suppression of potato powdery scab caused by *Spongospora subterranea* using an antagonistic fungus *Aspergillus versicolor* isolated from potato roots [Conference poster],” in Proceedings of the Ninth Symposium of the International Working Group on Plant Viruses with Fungal Vectors, Obihiro, Hokkaido, Japan, 19-22 August 2013. 53–54 (International Working Group on Plant Viruses with Fungal Vectors).

Naraghi, L., Heydari, A., and Ershad, D. (2006). Sporulation and survival of *Talaromyces flavus* on different plant material residues for biological control of cotton wilt caused by *Verticillium dahliae*. *Iranian J. Plant Pathol*. 42 (3): 381-397.

Naraghi, L., Heydari, A., Rezaee, S., Razavi, M., and Jahanifar, H. (2010a). Study on
antagonistic effects of *Talaromyces flavus* on *Verticillium albo-atrum*, the
causal agent of potato wilt disease. *Crop Protect.* 29 (7), 658–662. doi:
10.1016/j.cropro.2010.01.011

Naraghi, L., Heydari, A., Rezaee, S., Razavi, M., and Afshari-Azad, H. (2010b).
Biological control of Verticillium wilt of greenhouse cucumber by Talaromyces
flavus. *Phytopathol. Mediterranea*. 49 (3), 321–329. doi: 10.2478/v10045-010-
0061-x

Narisawa, K., Ohki, K. T., and Hashiba, T. (2000). Suppression of clubroot and
Verticillium yellows in Chinese cabbage in the field by the root endophytic
fungus, *Heteroconium chaetospira. Plant Pathol.* 49 (1), 141–146. doi: 10.1046/
j.1365-3059.2000.00425.x

Narisawa, K., Kawamata, H., Currah, R. S., and Hashiba, T. (2002). Suppression of
Verticillium wilt in eggplant by some fungal root endophytes*. Eur. J. Plant
Pathol.* 108 (2), 103–109. doi: 10.1023/A:1015080311041

Narisawa, K., Usuki, F., and Hashiba, T. (2004). Control of Verticillium yellows in
Chinese cabbage by the dark septate endophytic fungus LtVB3. *Phytopathology*94 (5), 412–418. doi: 10.1094/PHYTO.2004.94.5.412

Ngullie, M., Daiho, L., and Upadhyay, D. (2010). Biological management of fruit
rot in the world’s hottest chilli (*Capsicum chinense* Jacq.). *J. Plant Prot. Res*. 50
(3), 269–273

Nicolotti, G., Gonthier, P., and Varese, G. C. (1999). Effectiveness of some
biocontrol and chemical treatments against *Heterobasidion annosum* on
Norway spruce stumps. *Eur.* *J. For. Pathol*. 29 (5), 339–346. doi: 10.1046/
j.1439-0329.1999.00159.x

Nuangmek, W., McKenzie, E. H. C., and Lumyong, S. (2008). Endophytic fungi
from wild banana (*Musa acuminata* Colla) works against anthracnose disease
caused by *Colletotrichum musae. Res. J. Microbiol*. 3 (5), 368–374 doi: 10.3923/jm.2008.368.374

O’Brien, P. A. (2017). Biological control of plant diseases. *Australas. Plant Pathol*. 46 (4), 293–304. doi: 10.1007/s13313-017-0481-4

Odebode, A. C. (2006). Control of postharvest pathogens of fruits by culture
filtrate from antagonistic fungi. *J. Plant Prot. Res*. 46 (1), 1–5.

Ogbebor, N., Adekunle, A., Eghafona, N., and Ogboghodo, A. (2010). *Ganoderma
psuedoferreum:* biological control possibilities with microorganisms isolated
from soils of rubber plantations in Nigeria. *Afr. J. Agric. Res*. 6, 301–305.

Ogbebor, N. O., Adekunle, A. T., Eghafona, O. N., and Ogboghodo, A. I. (2015).
Biological control of *Rigidoporus lignosus* in *Hevea brasiliensis* in Nigeria.
*Fungal Biol.* 119 (1), 1–6. doi: 10.1016/j.funbio.2014.10.002

Okigbo, R. N., and Ikediugwu, F. E. O. (2000). Studies on biological control of
postharvest rot in yams (*Dioscorea* spp.) using. *Trichoderma Viride*. *J.
Phytopathol.* 148 (6), 351–355 doi.org/10.1046/j.1439-0434.2000.00515.x

Ordentlich, A., Nachmias, A., and Chet, I. (1990). Integrated control of *Verticillium dahlia* in potato by *Trichoderma harzianum* and captan. *Crop Prot*. 9 (5), 363–366. doi: 10.1016/0261-2194(90)90008-U

Padder, B. A., and Sharma, P. N. (2011).In vitro and in vivo antagonism of biocontrol agents against *Colletotrichum lindemuthianum* causing bean anthracnose. *Arch. Phytopathol. PlantProtect*. 44 (10), 961–969. doi:10.1080/03235400903460619

Padder, B. A., Sharma, P. N., Kapil, R., Pathania, A., and Sharma, O. P. (2010).
Evaluation of bioagents and biopesticides against *Colletotrichum
lindemuthianum* and its Integrated Management in Common Bean. *Notulae
Sci. Biol*. 2 (3), 72. doi: 10.15835/nsb234772

Pandey, R. R., Arora, D. K., and Dubey, R. C. (1993). Antagonistic interactions
between fungal pathogens and phylloplane fungi of guava. *Mycopathologia* 124
(1), 31–39. doi: 10.1007/BF01103054

Papasotiriou, F. G., Varypatakis, K. G., Christofi, N., Tjamos, S. E., and
Paplomatas, E. J. (2013). Olive mill wastes: a source of resistance for plants
against *Verticillium dahliae* and a reservoir of biocontrol agents. *Biol. Control.*67 (1), 51–60. doi: 10.1016/j.biocontrol.2013.07.008

Papavizas, G. C., and Lewis, J. A. (1989). Effect of *Gliocladium* and *Trichoderma* on
damping-off and blight of snapbean caused by *Sclerotium rolfsii* in the
greenhouse. *Plant Pathol*. 38 (2), 277–286. doi: 10.1111/j.1365-
3059.1989.tb02144.x

Parafati, L., Vitale, A., Restuccia, C., and Cirvilleri, G. (2015). Biocontrol ability
and action mechanism of food-isolated yeast strains against *Botrytis cinerea*
causing post-harvest bunch rot of table grape. *Food Microbiol.* 47, 85–92. doi:
10.1016/j.fm.2014.11.013

Paulitz, T. C., Park, C. S., and Baker, R. (1987). Biological control of Fusarium wilt
of cucumber with nonpathogenic isolates of *Fusarium oxysporum. Can. J.
Microbiol.* 33 (5), 349–353. doi: 10.1139/m87-061

Peng, G., McGregor, L., Lahlali, R., Gossen, B. D., Hwang, S. F., Adhikari, K. K.,
et al. (2011). Potential biological control of clubroot on canola and crucifer vegetable crops. *Plant Pathol.* 60 (3), 566–574. doi: 10.1111/j.1365-
3059.2010.02400.x

Perello, A. E., Monaco, C. I., Moreno, M. V., Cordo, C. A., and Simon, M. R.
(2006). The effect of *Trichoderma harzianum* and *T. koningii* on the control of
tan spot (*Pyrenophora tritici-repentis*) and leaf blotch (*Mycosphaerella
graminicola*) of wheat under field conditions in Argentina. *Biocontrol Sci.
Technol.* 16 (8), 803–813 doi.org/10.1080/09583150600700099

Perello, A., Monaco, C., Simon, M. R., Sisterna, M., and Dal Bello, G. S. (2003).
Biocontrol efficacy of Trichoderma isolates for tan spot of wheat in Argentina.
Crop Prot. 22 (9), 1099–1106. doi: 10.1016/S0261-2194(03)00143-1

Perelló, A. E., Moreno, M. V., Mónaco, C., Simón, M. R., and Cordo, C. (2009). Biological control of *Septoria tritici* blotch on wheat by *Trichoderma* spp. underfield conditions in Argentina. *Bio Control* 54 (1), 113–122. doi: 10.1007/s10526-008-9159-8

Perez, M. F., Contreras, L., Garnica, N. M., Fernández-Zenoff, M. V., Farı́ as, M. E., Sepulveda, M., et al. (2016). Native killer yeasts as biocontrol agents of postharvest fungal diseases in lemons. *PloS One* 11 (10), p.e0165590. doi: 10.1371/journal.pone.0165590

Pertot, I., Zasso, R., Amsalem, L., Baldessari, M., Angeli, G., and Elad, Y. (2008).
Integrating biocontrol agents in strawberry powdery mildew control strategies
in high tunnel growing systems. *Crop Prot.* 27 (3-5), 622–631. doi: 10.1016/
j.cropro.2007.09.004

Piano, S., Neyrotti, V., Migheli, Q., and Gullino, M. L. (1997). Biocontrol capability of *Metschnikowia pulcherrima* against *Botrytis* postharvest rot of apple. *Postharvest Biol. Technol*. 11 (3), 131–140. doi: 10.1016/S0925-5214(97)00022-7

Postma, J., and Rattink, H. (1992). Biological control of Fusarium wilt of carnation
with a nonpathogenic isolate of *Fusarium oxysporum. Can. J. Bot*. 70 (6),
1199–1205. doi: 10.1139/b92-150

Pradeep, K., and Kumud, K. (2000). Bio-control of seed-borne fungal pathogens of
pigeonpea (*Cajanus cajan* (L.) Millsp.). *Ann. Plant Prot. Sci*. 8 (1), 30–32.

Prasad, R. D., and Rangeshwaran, R. (2000). Effect of soil application of a granular
formulation of *Trichoderma harzianum* on seed rot and damping-off of
chickpea incited by *Rhizoctonia solani*, saprophytic growth of the pathogen
and bioagent proliferation. *J. Mycol. Plant Pathol*. 30, 216–220.

Prasad, R. D., Rangeshwaran, R., Hegde, S. V., and Anuroop, C. P. (2002). Effect of
soil and seed application of *Trichoderma harzianum* on pigeonpea wilt caused
by *Fusarium udum* under field conditions. *Crop Protect.* 21 (4), 293–297. doi:
10.1016/S0261-2194(01)00100-4

Pratella, G. C., and Mari, M. (1993). Effectiveness of *Trichoderma, Gliocladium*
and *Paecilomyces* in postharvest fruit protection. *Postharvest Biol. Technol*. 3
(1), 49–56. doi: 10.1016/0925-5214(93)90026-Y

Pretscher, J., Fischkal, T., Branscheidt, S., Jäger, L., Kahl, S., Schlander, M., et al.
(2018). Yeasts from different habitats and their potential as biocontrol agents.
*Fermentation* 4 (2), 31. doi: 10.3390/fermentation4020031

Promwee, A., Yenjit, P., Issarakraisila, M., Intana, W., and Chamswarng, C.
(2017). Efficacy of indigenous *Trichoderma harzianum* in controlling
Phytophthora leaf fall (*Phytophthora palmivora*) in Thai rubber trees. *J.
Plant Dis. Prot*. 124 (1), 41–50. doi: 10.1007/s41348-016-0051-y

Prusky, D., Freeman, S., Rodriguez, R. J., and Keen, N. T. (1994). A nonpathogenic
mutant strain of *Colletotrichum magna* induces resistance to *C.
gloeosporioides* in avocado fruits. *MPMI-Molecular Plant Microbe Interact*. 7
(3), 326–333. doi: 10.1094/MPMI-7-0326

Punja, Z. K., and Yip, R. (2003). Biological control of damping-off and root rot
caused by Pythium aphanidermatum on greenhouse cucumbers. *Can. J. Plant
Pathol*. 25 (4), 411–417. doi: 10.1080/07060660309507098

Qin, G. Z., and Tian, S. P. (2004). Biocontrol of postharvest diseases of jujube fruit by *Cryptococcus laurentii* combined with a low dosage of fungicides under different storage conditions. *Plant Dis*. 88 (5), 497–501. doi: 10.1094/PDIS.2004.88.5.497

Qin, G. Z., Tian, S. P., Xu, Y., Chan, Z. L., and Li, B. Q. (2006). Combination of
antagonistic yeasts with two food additives for control of brown rot caused by
*Monilinia fructicola* on sweet cherry fruit. *J. Appl. Microbiol.* 100 (3), 508–515.
doi: 10.1111/j.1365-2672.2005.02821.x

Qin, G., Tian, S., and Xu, Y. (2004). Biocontrol of postharvest diseases on sweet
cherries by four antagonistic yeasts in different storage conditions. *Postharvest
Biol. Technol.* 31 (1), 51–58. doi: 10.1016/S0925-5214(03)00130-3

Qin, Q. M., Vallad, G. E., and Subbarao, K. V. (2008). Characterization of
*Verticillium dahliae* and *V. tricorpu*s isolates from lettuce and artichoke.
*Plant Dis*. 92 (1), 69–77 doi: 10.1094/PDIS-92-1-0069.

Qing, F., and Shiping, T. (2000). Postharvest biological control of Rhizopus rot of
nectarine fruits by *Pichia membranefaciens. Plant Dis*. 84 (11), 1212–1216. doi:
10.1094/PDIS.2000.84.11.1212

Rabeendran, N., Jones, E. E., Moot, D. J., and Stewart, A. (2006). Biocontrol of
Sclerotinia lettuce drop by *Coniothyrium minitans* and *Trichoderma hamatum*.
*Biol. Control* 39 (3), 352–362. doi: 10.1016/j.biocontrol.2006.06.004

Rabha, A. J., Naglot, A., Sharma, G. D., Gogoi, H. K., and Veer, V. (2014). In vitro
evaluation of antagonism of endophytic *Colletotrichum gloeosporioides* against
potent fungal pathogens of *Camellia sinensis. Indian J. Microbiol.* 54 (3), 302–
309. doi: 10.1007/s12088-014-0458-8

Raguchander, T., Shanmugam, V., and Samiyappan, R. (2000). Biological control
of Panama wilt disease of banana. Madras Agric. J. 87 (4/6), 320–321 doi: 10.3389/fmicb.2019.00616

Rahman, M. A., Begum, M. F., and Alam, M. F. (2009). Screening of *Trichoderma*isolates as a biological control agent against *Ceratocystis paradoxa* causing
pineapple disease of sugarcane. *Mycobiology* 37 (4), 277–285. doi: 10.4489/
MYCO.2009.37.4.277

Rajakumar, E., Aggarwal, R., and Singh, B. (2005). Fungal antagonists for the
biological control of Ascochyta blight of chickpea. *Acta Phytopathol. Entomol.
Hungarica.* 40 (1-2), 35–42. doi: 10.1556/APhyt.40.2005.1-2.5

Rajesh, M., Anand, T., and Muthamilan, M. (2007). Biological control of cowpea
[*Vigna unguiculata* (L.) Walp.] root-rot caused by *Macrophomina phaseolina*
(Tassi.) Goid. by bacterial and fungal antagonists. *J. Biol. Control* 21 (1), 111–118.

Raziq, F., and Fox, R. T. V. (2004). Antagonistic activities of selected fungal isolates
against *Armillaria mellea*. *Biol. Agric. Horticult*. 22 (1), 41–56. doi: 10.1080/
01448765.2004.9754987

Raziq, F., and Fox, R. T. V. (2006). The integrated control of *Armillaria mellea* 2.
Field experiments. *Biol. Agric. Horticult*. 23 (3), 235–249. doi: 10.1080/
01448765.2006.9755326

Redmond, J. C., Marois, J. J., and MacDonald, J. D. (1987). Biological control of
*Botrytis cinerea* on roses with epiphytic microorganisms. *Plant Dis.* 71 (9),
799–802. doi: 10.1094/PD-71-0799

Reglinski, T., Elmer, P. A. G., Taylor, J. T., Parry, F. J., Marsden, R., and Wood, P.
N. (2005). Suppression of Botrytis bunch rot in Chardonnay grapevines by
induction of host resistance and fungal antagonism. *Australas. Plant Pathol.* 34
(4), 481–488. doi: 10.1071/AP05057

Reglinski, T., Elmer, P. A. G., Taylor, J. T., Wood, P. N., and Hoyte, S. M. (2010).
Inhibition of *Botrytis cinerea* growth and suppression of botrytis bunch rot in
grapes using chitosan. *Plant Pathol*. 59 (5), 882–890. doi: 10.1111/j.1365-
3059.2010.02312.x

Regliński, T., Rodenburg, N., Taylor, J. T., Northcott, G. L., Ah Chee, A., Spiers, T.
M., et al. (2012). *Trichoderma atroviride* promotes growth and enhances
systemic resistance to *Diplodia pinea* in radiata pine (*Pinus radiata*)
seedlings. *For. Pathol.* 42 (1), 75–78. doi.org/10.1111/j.1439-0329.2010.00710.x

Reyes, M. E. Q., Rohrbach, K. G., and Paull, R. E. (2004). Microbial antagonists
control postharvest black rot of pineapple fruit. *Postharvest Biol. Technol*. 33
(2), 193–203. doi: 10.1016/j.postharvbio.2004.02.003

Roberti, R., Ghisellini, L., and Innocenti, G. (1993). Biological control of blackleg of beet (*Phoma betae*) by *Metarhizium anisopliae*. *J. Plant Dis. Prot*., 100(2), 203-210.

Roberts, R. G. (1990a). Postharvest biological control of gray mold of apple by *Cryptococcus laurentii*. *Phytopathology* 80, 526–530. doi: 10.1094/Phyto-80-526

Roberts, R. G. (1990b). Biological control of mucor rot of pear by
*Cryptococcus laurentii, C. flavus*, and *C. albidus*. *Phytopathology* 80(6), 526-530 doi:10.1094/Phyto-80-526

Roberts, R. G. (1991). *Characterization of postharvest biological control of deciduous fruit diseases by* *Cryptococcus* spp (ARS-US Department of Agriculture, Agricultural Research Service (USA), Sheperdstown, West Virginia.

Rocha, A. C., Garcia, D., Uetanabaro, A. P., Carneiro, R. T., Araújo, I. S., Mattos, C.
R., et al. (2011). Foliar endophytic fungi from *Hevea brasiliensis* and their
antagonism on *Microcyclus ulei*. *Fungal Divers.* 47 (1), 75–84. doi: 10.1007/
s13225-010-0044-2

Roiger, D. J., and Jeffers, S. N. (1991). Evaluation of *Trichoderma* spp. for
biological control of Phytophthora crown and root rot of apple seedlings.
*Phytopathology* 81 (8), 910–917. doi: 10.1094/Phyto-81-910

Ronquist, F., and Huelsenbeck, J. P. (2003). MrBayes 3: Bayesian phylogenetic
inference under mixed models. *Bioinformatics* 19, 1572–1574. doi: 10.1093/
bioinformatics/btg18

Rosa, M. M., Tauk-Tornisielo, S. M., Rampazzo, P. E., and Ceccato-Antonini, S. R.
(2010). Evaluation of the biological control by the yeast *Torulaspora globosa*
against *Colletotrichum sublineolum* in sorghum. *World J. Microbiol. Biotechnol*.
26 (8), 1491–1502. doi: 10.1007/s11274-010-0324-8

Roy, G., Bussieres, G., Laflamme, G., and Dessureault, M. (2001). In vitro
inhibition of *Heterobasidion annosum* by *Phaeotheca dimorphospora*. *For.
Pathol.* 31 (6), 395–404. doi: 10.1046/j.1439-0329.2001.00259.x

Roy, G., Laflamme, G., Bussières, G., and Dessureault, M. (2003). Field tests on
biological control of *Heterobasidion annosum* by *Phaeotheca dimorphospora* in
comparison with *Phlebiopsis gigantea*. *For. Pathol*. 33 (2), 127–140. doi:
10.1046/j.1439-0329.2003.00319.x

Rubini, M. R., Silva-Ribeiro, R. T., Pomella, A. W., Maki, C. S., Araújo, W. L., Dos Santos, D. R., et al. (2005). Diversity of endophytic fungal community of cacao (*Theobroma cacao* L.) and biological control of *Crinipellis perniciosa*, causal agent of Witches’ Broom Disease. *Int. J. Biol. Sci*. 1 (1), 24-33. doi: 10.7150/ijbs.1.24

Rungjindamai, N., Xu, X. M., and Jeffries, P. (2013). Identification and
characterisation of new microbial antagonists for biocontrol of *Monilinia
laxa,* the causal agent of brown rot on stone fruit. *Agronomy* 3 (4), 685–703.
doi: 10.3390/agronomy3040685

Saber, M. M., Abdou, Y. A., El-Gantiry, S. M., and Ahmed, S. S. (2003). Biocontrol
of anthracnose disease of soybean caused by *Colletotrichum dematium. Egypt.
J. Phytopathol.* 31 (1/2), 17–29.

Sabuquillo, P., De Cal, A., and Melgarejo, P. (2005). Dispersal improvement of a
powder formulation of *Penicillium oxalicum*, a biocontrol agent of tomato wilt.
*Plant Dis*. 89 (12), 1317–1323. doi: 10.1094/PD-89-1317

Sahi, I. Y., and Khalid, A. N. (2007). In vitro biological control of *Fusarium
oxysporum* causing wilt in *Capsicum annuum. Mycopathology*  5, 85–88.

Saksirirat, W., and Hoppe, H. H. (1990). *Verticillium psalliotae*, an effective mycoparasite of the soybean rust fungus *Phakopsora pachyrhizi* Syd./*Verticillium psallisotae*, ein wirkungsvoller Mycoparasit des Sojabohnenrostes Phakopsora pachyrhizi Syd. *Z. für Pflanzenkrankheiten und Pflanzenschutz/Journal Plant Dis. Prot*., 97(3), 622-633.

Saldajeno, M. G. B., and Hyakumachi, M. (2011). The plant growth-promoting
fungus *Fusarium equiseti* and the arbuscular mycorrhizal fungus *Glomus
mosseae* stimulate plant growth and reduce severity of anthracnose and
damping-off diseases in cucumber (*Cucumis sativus*) seedlings. *Ann. Appl.
Biol.* 159 (1), 28–40. doi: 10.1111/j.1744-7348.2011.00471.x

Samuels, G. J., Pardo-schultheiss, R., Hebbar, K. P., Lumsden, R. D., Bastos, C. N.,
Costa, J. C., et al. (2000). *Trichoderma stromaticum* sp. nov., a parasite of the
cacao witches broom pathogen. *Mycol. Res*. 104 (6), 760–764.

Sangeetha, G., Usharani, S., and Muthukumar, A. (2009). Biocontrol with *Trichoderma* species for the management of postharvest crown rot of banana. *Phytopathol. Mediterr*. 48 (2), 214–225. doi: 10.14601/Phytopathol_Mediterr-2741

Sanogo, S., Pomella, A., Hebbar, P. K., Bailey, B., Costa, J. C. B., Samuels, G. J., et al.
(2002). Production and germination of conidia of *Trichoderma stromaticum*, a
mycoparasite of *Crinipellis perniciosa* on cacao. *Phytopathology* 92 (10), 1032–
1037. doi: 10.1094/PHYTO.2002.92.10.1032

Santamarıa, O., Smith, D. R., and Stanosz, G. R. (2012). Interaction between ́
*Diplodia pinea* or *Diplodia scrobiculata* and fungal endophytes isolated from
pine shoots. *Can. J. For. Res*. 42 (10), 1819–1826. doi: 10.1139/x2012-132

Schena, L., Ippolito, A., Zahavi, T., Cohen, L., Nigro, F., and Droby, S. (1999).
Genetic diversity and biocontrol activity of *Aureobasidium pullulans* isolates
against postharvest rots. *Postharvest Biol. Technol.* 17 (3), 189–199. doi:
10.1016/S0925-5214(99)00036-8

Schena, L., Nigro, F., Pentimone, I., Ligorio, A., and Ippolito, A. (2003). Control of
postharvest rots of sweet cherries and table grapes with endophytic isolates of
*Aureobasidium pullulans. Postharvest Biol. Technol.* 30 (3), 209–220. doi:
10.1016/S0925-5214(03)00111-X

Schisler, D. A., Khan, N. I., Boehm, M. J., and Slininger, P. J. (2002). Greenhouse andfield evaluation of biological control of Fusarium head blight on durum wheat. *Plant Dis*. 86 (12), 1350–1356. doi: 10.1094/PDIS.2002.86.12.1350

Schnatho, W. C., and Mathre, D. E. (1966). Cross-protection in cotton with strains
of *Verticillium albo-atrum. Phytopathology* 56 (10), 1204-1209.

Segarra, G., Avilés, M., Casanova, E., Borrero, C., and Trillas, I. (2013).
Effectiveness of biological control of *Phytophthora capsici* in pepper by
*Trichoderma asperellum* strain T34. *Phytopathol. Mediterranea*. 52 (1), 77–83 doi: 10.14601/Phytopathol_Mediterr-11242

Sennoi, R., Singkham, N., Jogloy, S., Boonlue, S., Saksirirat, W., Kesmala, T., et al.
(2013). Biological control of southern stem rot caused by *Sclerotium rolfsii*
using *Trichoderma harzianum* and arbuscular mycorrhizal fungi on Jerusalem
artichoke (*Helianthus tuberosus* L.). *Crop Protect.* 54, 148–153. doi: 10.1016/
j.cropro.2013.08.011

Shahid, S., and Khan, M. R. (2016). Biological control of root-rot on mung bean
plants incited by *Macrophomina phaseolina* through microbial antagonists.
*Plant Pathol. J*. 15 (2), 27–39

Shaikh, F. T., and Nasreen, S. (2013). Biocontrol efficacy of *Trichoderma koningii*
against some plant pathogenic fungi. *Indian J. Res.* 2 (3), 9–10.

Sharma, B. K., and Basandrai, A. K. (2000). Effectiveness of some fungicides and
biocontrol agents for the management of Karnal bunt of wheat. *J. Mycol. Plant
Pathol.* 30 (1), 76–78 .

Shittu, H. O., Castroverde, D. C., Nazar, R. N., and Robb, J. (2009). Plantendophyte interplay protects tomato against a virulent *Verticillium*. *Planta* 229
(2), 415–426. doi: 10.1007/s00425-008-0840-z

Shovan, L. R., Bhuiyan, M. K. A., Begum, J. A., and Pervez, Z. (2008). In vitro control of *Colletotrichum dematium* causing anthracnose of soybean by fungicides, plant
extracts and *Trichoderma harzianum. Int. J. Sustain. Crop Prod.* 3 (3), 10–17.

Shukla, A. N., and Anil, R. (1996). Inhibition of *Ganoderma lucidum* (Leyss) Karst
by *Polyporus sanguineus* Klotzsch in vitro. *Indian J. For*. 19 (1), 26–30.

Singh, D. (2004). Effect of *Debaryomyces hanseniiand* calcium salt on fruit rot of
peach (*Rhizopus macrosporus*). Ann. Plant Prot. Sci. 12 (2), 310–313.

Siri-udom, S., Suwannarach, N., and Lumyong, S. (2016). Existence of *Muscodor
vitigenus, M. equiseti* and *M. heveae* sp. nov. in leaves of the rubber tree (*Hevea
brasiliensis* Müll. Arg.), and their biocontrol potential. *Ann. Microbiol*. 66 (1),
437–448. doi: 10.1007/s13213-015-1126-x

Sivakumar, D., Wijeratnam, R. W., Wijesundera, R. L. C., Marikar, F. M. T., and
Abeyesekere, M. (2000). Antagonistic effect of *Trichoderma harzianum* on
postharvest pathogens of rambutan (*Nephelium lappaceum*). *Phytoparasitica*
28 (3), 240. doi: 10.1007/BF02981802

Sivan, A., and Chet, I. (1993). Integrated control of Fusarium crown and root rot of
tomato with *Trichoderma harzianum* in combination with methyl bromide or soil
solarization. *Crop Prot.* 12 (5), 380–386. doi: 10.1016/0261-2194(93)90082-T

Sivan, A., Ucko, O., and Chef, I. (1987). Biological control of fusarium crown rot of
tomato by *Trichoderrna harzianum* under field conditions. *Plant Dis*. 71, 587–
592. doi: 10.1094/PD-71-0587

Ślusarski, C., and Pietr, S. J. (2009). Combined application of dazomet and
*Trichoderma asperellum* as an efficient alternative to methyl bromide in
controlling the soil-borne disease complex of bell pepper. *Crop Protect.* 28
(8), 668–674. doi: 10.1016/j.cropro.2009.03.016

Solarska, E., Fravel, D., and Pietr, S. (2000). “Antagonistic action of *Talaromyces flavus*
and *Trichoderma viride* against *Verticillium albo-atrum* on hops,” in Advances in
Verticillium research and Disease Management. Eds. E. C. Tjamos, R. C. Rowe, J. B. Heale and D. R. Fravel (St. Paul, Mn, USA: APS press), Pages 237–239.

Somani, A. K., and Arora, R. K. (2010). Field efficacy of *Trichoderma viride,
Bacillus subtilis* and *Bacillus cereus* in consortium for control of *Rhizoctonia
solani* causing black scurf disease of potato. *Indian Phytopathol*. 63 (1), 63: 23-25.

Soytong, K., and Kaewchai, S. (2014). Biological control of white root of rubber
trees using *Chaetomium cupreum. J. Agric. Tech*. 10 (1), 93–103.

Soytong, K., Pongak, W., and Kasiolarn, H. (2005). Biological control of
Thielaviopsis bud rot of *Hyophorbe lagenicaulis* in the field. *J. Agric. Technol*.
1 (2), 235–245.

Spadaro, D., Vola, R., Piano, S., and Gullino, M. L. (2002). Mechanisms of action and efficacy of four isolates of the yeast *Metschnikowia pulcherrima* active against postharvest pathogens on apples. *Postharvest Biol. Technol*. 24 (2), 123–134. doi: 10.1016/S0925-5214(01)00172-7

Spadaro, D., Garibaldi, A., and Gullino, M. L. (2004). Control of *Penicillium expansum*
and *Botrytis cinerea* on apple combining a biocontrol agent with hot water dipping and acibenzolar-S-methyl, baking soda, or ethanol application. *Postharvest Biol*. *Technol.* 33 (2), 141–151. doi: 10.1016/j.postharvbio.2004.02.002

Spotts, R. A., Cervantes, L. A., Facteau, T. J., and Chand-Goyal, T. (1998). Control of brown rot and blue mold of sweet cherry with preharvest iprodione, postharvest Cryptococcus infirmo-miniatus, and modified atmosphere packaging. *Plant Dis*. 82 (10), 1158–1160. doi: 10.1094/PDIS.1998.82.10.1158

Sriram, S., and Poornadchanddra, S. R. (2013). Biological control of postharvest mango fruit rot caused by *Colletotrichum gloeosporioides* and *Diplodia natalensis* with *Candida tropicalis* and *Alcaligenes feacalis*. *Indian Phytopathol*. 66 (4), 375–380.

Srivastava, A. K., Défago, G., and Kern, H. (1985). Hyperparasitism of *Puccinia
horiana* and other microcyclic rusts. *J. Phytopathol.* 114 (1), 73–78. doi:
10.1111/j.1439-0434.1985.tb04338.x

Stadler, M., and von Tiedemann, A. (2014). Biocontrol potential of
*Microsphaeropsis ochracea* on microsclerotia of *Verticillium longisporum* in
environments differing in microbial complexity. *Biocontrol* 59 (4), 449–460.
doi: 10.1007/s10526-014-9586-7

Stinson, A. M., Zidack, N. K., Strobel, G. A., and Jacobsen, B. J. (2003).
Mycofumigation with *Muscodor albus* and *Muscodor roseus* for control of
seedling diseases of sugar beet and Verticillium wilt of eggplant. *Plant Dis.* 87
(11), 1349–1354. doi: 10.1094/PDIS.2003.87.11.1349

Stirling, M., and Stirling, G. (1997). “Disease Management: Biological Control,” in Plant Pathogens and Plant Diseases. *Eds. J. Brown and H. Ogle*, *Rockvale Publications, Armidale* 427–439.

Sudirman, L. I., Housseini, A. I., Le Febvre, G., Kiffer, E., and Botton, B. (1992).
Screening of some basidiomycetes for biocontrol of *Rigidoporus lignosus*, a
parasite of the rubber tree *Hevea brasiliensis. Mycol. Res.* 96 (8), 621–625. doi:
10.1016/S0953-7562(09)80486-3

Suresh, N., and Nelson, R. (2016). Isolation of antagonistic fungi and evaluation of
antifungal activity of the separated metabolite against the red rot of sugarcane
pathogen. *Eur. J. Exp. Biol.* 6, 15–21

Suriyagamon, S., Phonkerd, N., Bunyatratchata, W., Riddech, N., and
Mongkolthanaruk, W. (2018). Compost Seed of *Trichoderma harzianum*
UD12-102 in Controlling Collar and Stem Rot of Tomato Caused by
*Sclerotium rolfsii*. *Environ. Natural Resour. J*. 16 (2), 20–28. doi:10.14456/ennrj.2018.12

Susanto, A., Sudharto, P. S., and Purba, R. Y. (2005). Enhancing biological control
of basal stem rot disease (*Ganoderma boninense*) in oil palm plantations.
*Mycopathologia* 159 (1), 153–157. doi: 10.1007/s11046-004-4438-0

Sztejnberg, A., Galper, S., Mazar, S., and Lisker, N. (1989). *Ampelomyces quisqualis*
for biological and integrated control of powdery mildews in Israel. *J.
Phytopathol*. 124 (4), 285–295. doi: 10.1111/j.1439-0434.1989.tb04925.x

Talubnak, C., and Soytong, K. (2010). Biological control of vanilla anthracnose
using *Emericella nidulans. J. Agric. Technol*. 6 (1), 47–55.

Talukder, M. I., Begum, F., and Azad, M. M. K. (2007). Management of pineapple disease of sugarcane through biological means. *J. Agric. Rural Dev*., 5(1 & 2), 79-83. doi.org/10.3329/jard.v5i1.1462

Thakur, S., and Harsh, N. S. K. (2014). Phylloplane fungi as biocontrol agent
against Alternaria leaf spot disease of (Akarkara) Spilanthesoleracea. *Biosci.
Discov*. 5 (2), 139–144.

Thangavelu, R., Palaniswami, A., and Velazhahan, R. (2004). Mass production of
*Trichoderma harzianum* for managing fusarium wilt of banana. *Agric. Ecosyst.
Environ.* 103 (1), 259–263. doi: 10.1016/j.agee.2003.09.026

Tian, S., Fan, Q., Xu, Y., and Liu, H. (2002a). Biocontrol efficacy of antagonist
yeasts to gray mold and blue mold on apples and pears in controlled
atmospheres. *Plant Dis*. 86 (8), 848–853. doi: 10.1094/PDIS.2002.86.8.848

Tian, S., Qin, G., and Xu, Y. (2005). Synergistic effects of combining biocontrol agents with silicon against postharvest diseases of jujube fruit. *J. Food Protect*. 68 (3), 544–550. doi: 10.4315/0362-028X-68.3.544

Torres, R., Teixido, N., Vinas, I., Mari, M., Casalini, L., Giraud, M., et al. (2006).
Efficacy of Candida sake CPA-1 formulation for controlling *Penicillium
expansum* decay on pome fruit from different Mediterranean regions. *J. Food
Protect.* 69 (11), 2703–2711. doi: 10.4315/0362-028X-69.11.2703

Tyvaert, L., França, S. C., Debode, J., and Höfte, M. (2014). The endophyte *Verticillium* V t305 protects cauliflower against Verticillium wilt. *J. Appl.
Microbiol.* 116 (6), 1563–1571. doi: 10.1111/jam.12481

Upadhyay, J. P., and Mukhopadhyay, A. N. (1986). Biological control of
*Sclerotium rolfsii* by *Trichoderma harzianum* in sugarbeet. *Int. J. Pest
Manage*. 32 (3), 215–220 doi.org/10.1080/09670878609371066

Urquhart, E. J., Menzies, J. G., and Punja, Z. K. (1994). Growth and biological
control activity of *Tilletiopsis* species against powdery mildew (*Sphaerotheca
fuliginea*) on greenhouse cucumber. *Phytopathology* 84 (4), 341–351. doi:
10.1094/Phyto-84-341

Usall, J., Teixido, N., Torres, R., de Eribe, X. O., and Viñas, I. (2001). Pilot tests of
Candida sake (CPA-1) applications to control postharvest blue mold on apple
fruit. *Postharvest Biol. Technol.* 21 (2), 147–156. doi: 10.1016/S0925-5214(00)
00131-9

Vagelas, I., and Leontopoulos, S. (2015). Cross-protection of cotton against Verticillium wilt by *Verticillium nigrescens*. Emirates *J. Food Agric*., 27(9): 687-691. doi: 10.9755/ejfa.2015-04-047

Vandermeer, J., Perfecto, I., and Liere, H. (2009). Evidence for hyperparasitism of
coffee rust (*Hemileia vastatrix*) by the entomogenous fungus, *Lecanicillium
lecanii*, through a complex ecological web. *Plant Pathol.* 58 (4), 636–641. doi:
10.1111/j.1365-3059.2009.02067.x

Vannacci, G., and Harman, G. E. (1987). Biocontrol of seed-borne *Alternaria raphani
and A. brassicicola. Can. J. Microbiol*. 33 (10), 850–856. doi: 10.1139/m87-149

Varo, A., Raya-Ortega, M. C., and Trapero, A. (2016). Selection and evaluation of micro-organisms for biocontrol of *Verticillium dahlia* in olive. *J. Appl. Microbiol*. 121 (3), 767–777. doi: 10.1111/jam.13199

Vasanthakumari, M. M., and Shivanna, M. B. (2013). Biological control of
anthracnose of chilli with rhizosphere and rhizoplane fungal isolates from
grasses. *Arch. Phytopathol. Plant Prot.* 46 (14), 1641–1666. doi: 10.1080/
03235408.2013.771901

Veloso, J., and Dıaz, J. (2012). ́ *Fusarium oxysporum* Fo47 confers protection to
pepper plants against *Verticillium dahliae* and *Phytophthora capsici*, and
induces the expression of defence genes. *Plant Pathol*. 61 (2), 281–288. doi:
10.1111/j.1365-3059.2011.02516.x

Verhaar, M. A., and Hijwegen, T. (1993). Efficient production of phialoconidia of
*Verticillium lecanii* for biocontrol of cucumber powdery mildew, *Sphaerotheca
fuliginea. Eur. J. Plant Pathol*. 99 (2), 101–103. doi: 10.1007/BF01998478

Verhaar, M. A., Kerssies, A., and Hijwegen, T. (1999). Effect of relative humidity
on mycoparasitism of rose powdery mildew with and without treatments with
mycoparasites/Der Einfluss von relativer Luftfeuchtigkeit auf die Bekämpfung
von Echtem Mehltau an Rosen mit und ohne Behandlungen mit
Mykoparasiten. *Z. für Pflanzenkrankheiten und Pflanzenschutz/Journal Plant
Dis. Prot*. 106 (2), 158–165.

Verma, N., MacDonald, L., and Punja, Z. K. (2006). Inoculum prevalence, host
infection and biological control of *Colletotrichum acutatum*: causal agent of
blueberry anthracnose in British Columbia. *Plant Pathol.* 55 (3), 442–450. doi:
10.1111/j.1365-3059.2006.01401.x

Vigo, C., Norman, J. R., and Hooker, J. E. (2000). Biocontrol of the pathogen
*Phytophthora parasitica* by arbuscular mycorrhizal fungi is a consequence of
effects on infection loci. *Plant Pathol.* 49 (4), 509–514. doi: 10.1046/j.1365-
3059.2000.00473.x

Vinas, I., Usall, J., Teixidó, N., and Sanchis, V. (1998). Biological control of major
postharvest pathogens on apple with Candida sake. *Int. J. Food Microbiol.* 40
(1-2), 9–16. doi: 10.1016/S0168-1605(98)00009-9

Wang, Y., Bao, Y., Shen, D., Feng, W., Yu, T., Zhang, J., et al. (2008). Biocontrol of
*Alternaria alternata* on cherry tomato fruit by use of marine yeast
*Rhodosporidium paludigenum* Fell & Tallman. *Int. J. Food Microbiol.* 123
(3), 234–239. doi: 10.1016/j.ijfoodmicro.2008.02.002

Wang, Y., Wang, P., Xia, J., Yu, T., Lou, B., Wang, J., et al. (2010). Effect of water
activity on stress tolerance and biocontrol activity in antagonistic yeast
*Rhodosporidium paludigenum. Int. J. Food Microbiol.* 143 (3), 103–108. doi:
10.1016/j.ijfoodmicro.2010.07.035

Ward, N. A., Robertson, C. L., Chanda, A. K., and Schneider, R. W. (2012). Effects
of *Simplicillium lanosoniveum* on *Phakopsora pachyrhizi*, the soybean rust
pathogen, and its use as a biological control agent. *Phytopathology* 102 (8),
749–760. doi: 10.1094/PHYTO-01-11-0031

Wijesinghe, C. J., Wijeratnam, R. W., Samarasekara, J. K. R. R., and Wijesundera,
R. L. C. (2010). Biological control of *Thielaviopsis paradoxa* on pineapple by an
isolate of *Trichoderma asperellum. Biol. Control.* 53 (3), 285–290. doi: 10.1016/
j.biocontrol.2010.02.009

Wilson, C. L., and Chalutz, E. (1989). Postharvest biological control of Penicillium
rots of citrus with antagonistic yeasts and bacteria. *Sci. Horticult.* 40 (2), 105–
112. doi: 10.1016/0304-4238(89)90092-7

Wilson, C. L., Wisniewski, M. E., Biles, C. L., McLaughlin, R., Chalutz, E., and Droby, S. (1991). Biological control of post-harvest diseases of fruits and vegetables: alternatives to synthetic fungicides. *Crop Protect*. 10 (3), 172–177. doi: 10.1016/0261-2194(91)90039-T

Wittig, H. P. P., Johnson, K. B., and Pscheidt, J. W. (1997). Effect of epiphytic fungi
on brown rot blossom blight and latent infections in sweet cherry. *Plant Dis.* 81
(4), 383–387. doi: 10.1094/PDIS.1997.81.4.383

Wisniewski, M., Wilson, C., Chalutz, E., and Hershberger, W. (1988).“Biological control of postharvest diseases of fruit: inhibition of Botrytis rot on apple by an antagonistic yeast,” in *Proceedings annual meeting, Electron Microscopy Society of America*, San Francisco Press, United States.

Wokocha, R. C., Ebenebe, A. C., and Erinle, I. D. (1986). Biological control of the
basal stem rot disease of tomato caused by *Corticium rolfsii* (Sacc.) Curzi in
Northern Nigeria. *Int. J. Pest Manage*. 32 (1), 35–39

Wu, C., Hsiang, T., Yang, L., and Liu, L. X. (1998). Efficacy of *Typhula phacorrhiza*
as a biocontrol agent of grey snow mould of creeping bentgrass. *Can. J. Bot*. 76
(7), 1276–1281. doi.org/10.1139/b98-114

Xi, L., and Tian, S. (2005). Control of postharvest diseases of tomato fruit by
combining antagonistic yeast with sodium bicarbonate. *Zhongguo nongye
kexue*. 38 (5), 950–955. doi: 10.3923/ppj.2017.62.72

Xu, B., Zhang, H., Chen, K., Xu, Q., Yao, Y., and Gao, H. (2013). Biocontrol of
postharvest Rhizopus decay of peaches with *Pichia caribbica. Curr. Microbiol*.
67 (2), 255–261. doi: 10.1007/s00284-013-0359-9

Xue, A., Chen, Y., Voldeng, H., Savard, M., and Tian, X. (2008). Biological control
of Fusarium head blight of wheat with *Clonostachys rosea* strain ACM941.
*Cereal Res. Commun.* 36(Supplement 6), 695–699. doi: 10.1556/
CRC.36.2008.Suppl.B.62

Yang, D., Plante, F., Bernier, L., Piché, Y., Dessureault, M., Laflamme, G., et al.
(1993). Evaluation of a fungal antagonist, *Phaeotheca dimorphospora*, for
biological control of tree diseases. *Can. J. Bot.* 71 (3), 426–433. doi.org/10.1139/b93-047

Yang, D., Bernier, L., and Dessureault, M. (1994). 2062301. Biological control of
Septoria leaf spot of poplar by *Phaeotheca dimorphospora*. *Plant Dis.* 78 (8),
821–825. doi: 10.1094/PD-78-0821

Yang, D., Laflamme, G., Bernier, L., and Dessureault, M. (1995). *Phaeotheca
dimorphospora* as a potential biocontrol agent for shoot blight caused by
*Gremmeniella abietina. Can. J. Plant Pathol*. 17 (1), 7–12. doi: 10.1080/
07060669509500713

Yao, H. J., and Tian, S. P. (2005). Effects of a biocontrol agent and methyl
jasmonate on postharvest diseases of peach fruit and the possible mechanisms
involved. *J. Appl. Microbiol.* 98 (4), 941–950. doi: 10.1111/j.1365-
2672.2004.02531.x

Yates, I. E., Meredith, F., Smart, W., Bacon, C. W., and Jaworski, A. J. (1999).
*Trichoderma viride* suppresses fumonisin B1 production by *Fusarium
moniliforme.* *J. Food Prot.* 62 (11), 1326–1332. doi: 10.4315/0362-028X-
62.11.1326

Yuan, Y., Feng, H., Wang, L., Li, Z., Shi, Y., Zhao, L., et al. (2017). Potential of
endophytic fungi isolated from cotton roots for biological control against verticillium wilt disease. *PloS One* 12 (1), p.e0170557. doi: 10.1371/
journal.pone.0170557

Zegeye, E. D., Santhanam, A., Gorfu, D., Tessera, M., and Kassa, B. (2011).
Biocontrol activity of *Trichoderma viride* and *Pseudomonas fluorescens* against
Phytophthora infestans under greenhouse conditions. *J. Agric. Technol*. 7 (6),
1589–1602

Zhang, H., Zheng, X., Fu, C., and Xi, Y. (2005a). Postharvest biological control of
gray mold rot of pear with *Cryptococcus laurentii*. *Postharvest Biol. Technol*. 35
(1), 79–86. doi: 10.1016/j.postharvbio.2004.03.011

Zhang, H. Y., Zheng, X. D., and Xi, Y. F. (2005b). Biological control of postharvest
blue mold of oranges by *Cryptococcus laurentii* (Kufferath) Skinner. *Biol. Control* 50 (2), 331–342. doi: 10.1007/s10526-004-0452-x

Zhang, H., Zheng, X., and Yu, T. (2007a). Biological control of postharvest diseases
of peach with *Cryptococcus laurentii*. *Food Control* 18 (4), 287–291. doi:
10.1016/j.foodcont.2005.10.007

Zhang, H., Zheng, X., Wang, L., Li, S., and Liu, R. (2007b). Effect of yeast
antagonist in combination with hot water dips on postharvest Rhizopus rot of
strawberries. *J. Food Eng*. 78 (1), 281–287. doi: 10.1016/j.jfoodeng.2005.09.027

Zhang, H., Wang, L., Ma, L., Dong, Y., Jiang, S., Xu, B., et al. (2009). Biocontrol of
major postharvest pathogens on apple using *Rhodotorula glutinis* and its effects
on postharvest quality parameters. *Biol. Control* 48 (1), 79–83. doi: 10.1016/
j.biocontrol.2008.09.004

Zhang, Q., Zhang, J., Yang, L., Zhang, L., Jiang, D., Chen, W., et al. (2014). Diversity and biocontrol potential of endophytic fungi in *Brassica napus*. *Biol. Control*. 72, 98–108. doi: 10.1016/j.biocontrol.2014.02.018

Zhao, Y., Tu, K., Shao, X., Jing, W., and Su, Z. (2008). Effects of the yeast *Pichia
guilliermondii* against *Rhizopus nigricans* on tomato fruit. *Postharvest Biol.
Technol.* 49 (1), 113–120. doi: 10.1016/j.postharvbio.2008.01.001

Zheng, X., Zhang, H., and Xi, Y. (2004). Effects of *Cryptococcus laurentii* (Kufferath) Skinner on biocontrol of postharvest decay of arbutus berries. *Bot. Bull. Acad. Sin*. 45: 55-60.

Zheng, Y., Xue, Q. Y., Xu, L. L., Xu, Q., Lu, S., Gu, C., et al. (2011). A screening strategy of fungal biocontrol agents towards Verticillium wilt of cotton. *Biol. Contro l*56 (3), 209–216. doi: 10.1016/j.biocontrol.2010.11.010

Zhimo, V. Y., Bhutia, D. D., and Saha, J. (2016). Biological control of post harvest
fruit diseases using antagonistic yeasts in India. *J. Plant Pathol*. 98 (2), 275–283 doi: 10.4454/JPP.V98I2.026

Zhou, T. I. N. G., and Reeleder, R. D. (1989). Application of *Epicoccum
purpurascens* spores to control white mold of snap bean. *Plant Dis.* 73 (8),
639–642. doi: 10.1094/PD-73-0639

Zhou, T., Schneider, K. E., and Li, X. Z. (2008). Development of biocontrol agents
from food microbial isolates for controlling post-harvest peach brown rot
caused by *Monilinia fructicola*. *Int. J. Food Microbiol*. 126 (1-2), 180–185. doi:
10.1016/j.ijfoodmicro.2008.05.020

Zhu, H. Q., Feng, Z. L., Li, Z. F., Shi, Y. Q., Zhao, L. H., and Yang, J. R. (2013). Characterization of two fungal isolates from cotton and evaluation of their potential for biocontrol of Verticillium wilt of cotton.J. *Phytopathol*. 161 (2), 70–77. doi: 10.1111/jph.12027

Živković, S., Stojanović, S., Ivanović, Ž., Gavrilović, V., Popović, T., and Balaž, J. (2010). Screening of antagonistic activity of microorganisms against *Colletotrichum acutatum* and *Colletotrichum gloeosporioides*. *Arch. Biol. Sci.* 62 (3), 611–623. doi:10.2298/ABS1003611Z
