# Supplementary material for: Fungi vs. Fungi in Biocontrol: An Overview of Fungal Antagonists Applied Against Fungal Plant Pathogens
Source: Front Cell Infect Microbiol. 2020 Nov 30;10:604923. doi: 10.3389/fcimb.2020.604923 (PMC7734056; doi:10.3389/fcimb.2020.604923)
Supplement: Supplementary file 2 [file Table_2.doc]

**Supplementary Table 2.** GenBank and culture collection accession numbers of species treated in the phylogeny. Type strains are given in bold typeface.

| **Species name** | **Strain/Isolate number** | **GenBank accession number (LSU)** |
| --- | --- | --- |
| ***Absidia cylindrospora*** | **CBS 153.63** | **JN206594** |
| *Akanthomyces attenuatus* | CBS 402.78 | AF339565 |
| ***Akanthomyces dipterigenus*** | **CBS 126.27** | **NG_058105** |
| *Akanthomyces lecanii* | CCFC 226713 | AY283556 |
| ***Albifimbria verrucaria*** | **CBS 328.52** | **MH868597** |
| ***Alternaria alternata*** | **CBS 916.96** | **DQ678082** |
| ***Alternaria atra*** | **CBS 195.67** | **MH870634** |
| *Alternaria brassicae* | CBS 116528 | KC584258 |
| ***Alternaria oudemansii*** | **CBS 114.07** | **MH866103** |
| *Alternaria porri* | CBS 116698 | KC584297 |
| *Alternaria solani* | CBS 116651 | KC584306 |
| *Alternaria tenuissima* | CBS 918.96 | KC584311 |
| ***Ampelomyces quisqualis*** | **CBS 129.79** | **MH872955** |
| ***Anthracocystis flocculosa*** | **CBS 167.88** | **NG_058538** |
| *Aphanocladium album* | CBS 411.34 | MH867096 |
| *Arcopilus cupreus* | CBS 320.67 | MH877794 |
| *Aspergillus aculeatus* | CBS 186.67 | MH870630 |
| ***Aspergillus caespitosus*** | **CBS 103.45** | **MH867639** |
| ***Aspergillus carneus*** | **CBS 494.65** | **MH870323** |
| ***Aspergillus cervinus*** | **CBS 196.64** | **MH870042** |
| *Aspergillus flavipes* | CBS 128797 | MH876587 |
| ***Aspergillus flavus*** | **ATCC 16883** | **NG_055742** |
| *Aspergillus flavus* | CBS 128202 | MH878005 |
| ***Aspergillus fumigatus*** | **ATCC 1022** | **AY660917** |
| *Aspergillus fumigatus* | CBS 500.61 | MH878549 |
| ***Aspergillus nidulans*** | **CBS 589.65** | **MH870364** |
| ***Aspergillus niger*** | **CBS 554.65** | **MH870353** |
| ***Aspergillus ochraceus*** | **CBS 108.08** | **MH866108** |
| *Aspergillus oryzae* | CBS 126859 | MH875717 |
| *Aspergillus terreus* | CBS 132948 | MH878509 |
| ***Aspergillus versicolor*** | **ATCC 9577** | **NG_055743** |
| *Athelia bombacina* | ATCC 20629 | AF279377 |
| ***Aureobasidium pullulan*** | **CBS 584.75** | **DQ470956** |
| *Aureobasidium pullulan* | MFLUCC 14-0288 | KM461701 |
| *Bionectria ochroleuca* | CCFC 226708 | AY283558 |
| ***Bipolaris oryzae*** | **MFLUCC 10-0715** | **JX256384** |
| *Botrytis cinerea* | CBS 125.58 | MH877779 |
| ***Buckleyzyma aurantiaca*** | **CBS 317** | **NG_058618** |
| *Byssochlamys spectabilis* | CBS 339.51 | MH868409 |
| ***Candida maritima*** | **CBS 5107** | **KY106562** |
| *Candida membranifaciens* | CBS 4430 | KY106568 |
| ***Candida oleophila*** | **CBS 2219** | **KY106621** |
| ***Candida quercitrusa*** | **CBS 4412** | **KY106713** |
| ***Candida saitoana*** | **CBS 940** | **KY106730** |
| ***Candida sake*** | **CBS 159** | **KY106745** |
| ***Candida tropicalis*** | **CBS 94** | **KY106838** |
| ***Chaetomium cochliodes*** | **CBS 155.52** | **MH868494** |
| ***Chaetomium globosum*** | **CBS 160.62** | **MH869713** |
| *Chrysocorona lucknowensis* | CBS 243.84 | MH877835 |
| ***Citeromyces matritensis*** | **CBS 2764** | **KY106924** |
| *Cladophialophora chaetospira* | CBS 755.68 | MH878422 |
| ***Cladosporium cladosporioides*** | **CBS 112388** | **KX286982** |
| ***Cladosporium colocasia*** | **ATCC 200944** | **AY342121** |
| ***Cladosporium herbarum*** | **CBS 121621** | **MH874676** |
| *Cladosporium uredinicola* | CBS 306.84 | DQ008147 |
| ***Clavispora fructus*** | **CBS 6380** | **KY106454** |
| ***Clonostachys byssicola*** | **CBS 364.78** | **MH872912** |
| ***Clonostachys rosea*** | **CBS 710.86** | **MH873700** |
| *Collariella bostrychodes* | CBS 586.83 | MH873374 |
| *Colletotrichum gloeosporioides* | CGMCC:LC0555 | JN940412 |
| *Coniochaeta ligniaria* | CBS 619.69 | MH871158 |
| ***Curvularia australiensis*** | **CBS 126973** | **NG_064236** |
| ***Curvularia lunata*** | **CBS 730.96** | **HF934900** |
| ***Curvularia pallescens*** | **CBS 156.35** | **MH867124** |
| ***Cystofilobasidium infirmominiatum*** | **CBS 323** | **KY107467** |
| ***Debaryomyces hansenii*** | **CBS 767** | **KY107531** |
| *Didymella pomorum* | CBS 539.66 | GU238028 |
| ***Diutina catenulata*** | **CBS 565** | **NG_059158** |
| ***Epicoccum nigrum*** | **CBS 173.73** | **GU237975** |
| ***Eutypa lata*** | **CBS 208.87** | **MH873755** |
| ***Exophiala jeanselmei*** | **CBS 507.90** | **MH873915** |
| *Funneliformis mosseae* | AFTOL-ID 139 | DQ273793 |
| *Fusarium chlamydosporum* | CBS 119843 | MH874619 |
| *Fusarium culmorum* | CBS 129.73 | MH872347 |
| ***Fusarium equiseti*** | **CBS 307.94** | **MH874117** |
| *Fusarium fujikuroi* | CBS 183.29 | MH866503 |
| *Fusarium graminearum* | CBS 131265 | MH877360 |
| *Fusarium heterosporum* | CBS 391.68 | MH870883 |
| *Fusarium incarnatum* | CBS 132.73 | MH878482 |
| *Fusarium lateritium* | CBS 127047 | MH875837 |
| *Fusarium oxysporum* | CBS 130301 | MH877318 |
| *Fusarium tricinctum* | CBS 253.50 | MH868113 |
| ***Fusarium verticillioides*** | **CBS 576.78** | **MH872939** |
| *Ganoderma lucidum* | CBS 270.81 | MH873099 |
| *Gibellulopsis nigrescens* | CBS 470.64 | MH870121 |
| ***Gjaerumia minor*** | **CBS 543.50** | **MH868274** |
| ***Gliomastix roseogrisea*** | **CBS 134.56** | **MH869081** |
| *Globisporangium ultimum* | CBS 398.51 | HQ665227 |
| *Haematonectria haematococca* | CBS 126407 | MH875565 |
| ***Hanseniaspora opuntiae*** | **CBS 8733** | **NG_055312** |
| ***Hanseniaspora uvarum*** | **CBS 314** | **KY107844** |
| *Hyphochytrium catenoides* | PL AUS 045 | EF594059 |
| *Hypomyces rosellus* | CBS 817.69 | MH871216 |
| *Induratia alba* | 9-6 | HM034865 |
| ***Laetisaria arvalis*** | **CBS 131.82** | **MH873229** |
| ***Lecanicillium psalliotae*** | **CBS 505.48** | **MH867993** |
| *Lentinus squarrosulus* | FRIM4180 | KP283517 |
| *Leptosphaeria biglobosa* | CBS 475.81 | MH873120 |
| ***Lipomyces tetrasporus*** | **CBS 5910** | **JN940885** |
| ***Metarhizium anisopliae*** | **ARSEF 7487** | **MH604974** |
| ***Metschnikowia lunata*** | **CBS 5946** | **KY108486** |
| ***Metschnikowia pulcherrima*** | **CBS 5833** | **KY108497** |
| ***Meyerozyma guilliermondii*** | **CBS 2030** | **KY108542** |
| *Microdochium bolleyi* | CBS 172.63 | MH869857 |
| *Microsphaeropsis arundinis* | CBS 100243 | JX496123 |
| *Minimedusa polyspora* | CBS 113.16 | MH866167 |
| ***Moesziomyces aphidis*** | **CBS 517.83** | **MH873352** |
| ***Moesziomyces rugulosus*** | **JCM 10323** | **JN940523** |
| ***Naganishia albida*** | **CBS 142** | **KY106955** |
| ***Nakazawaea ernobii*** | **CBS 1737** | **KY108637** |
| *Neocamarosporium betae* | CBS 109410 | EU754178 |
| *Neocosmospora solani* | CBS 132189 | MH877443 |
| *Nigrospora oryzae* | CBS 384.69 | MH871071 |
| ***Ogataea methanolica*** | **CBS 6515** | **KY108680** |
| ***Papiliotrema flavescens*** | **CBS 942** | **AB035042** |
| ***Papiliotrema laurentii*** | **CBS 139** | **KY108739** |
| *Paraboeremia putaminum* | CBS 130.69 | GU238138 |
| ***Paramyrothecium roridum*** | **CBS 357.89** | **KU846330** |
| *Paraphaeosphaeria minitans* | CBS 122788 | EU754173 |
| *Paraphaeosphaeria minitans* | CBS 122786 | EU754174 |
| ***Parasarocladium breve*** | **CBS 150.62** | **NG_056979** |
| ***Penicillium chrysogenum*** | **CBS 306.48** | **MH867907** |
| ***Penicillium citreonigrum*** | **CBS 258.29** | **MH866526** |
| ***Penicillium citrinum*** | **CBS 139.45** | **MH867647** |
| *Penicillium copticola* | CBS 127356 | MH875975 |
| ***Penicillium expansum*** | **CBS 325.48** | **MH867916** |
| ***Penicillium glabrum*** | **CBS 105.11** | **MH866126** |
| ***Penicillium herquei*** | **CBS 336.48** | **MH867921** |
| ***Penicillium olsonii*** | **CBS 232.60** | **MH869516** |
| ***Penicillium oxalicum*** | **CBS 219.30** | **MH866571** |
| ***Penicillium roqueforti*** | **CBS 221.30** | **MH866573** |
| ***Penicillium simplicissimum*** | **CBS 372.48** | **MH867949** |
| ***Penicillium spinulosum*** | **CBS 374.48** | **MH867951** |
| *Penicillium striatisporum* | CBS 706.68 | MH870927 |
| ***Penicillium sublateritium*** | **CBS 267.29** | **MH866531** |
| *Penicillium sumatraense* | CBS 130377 | MH877218 |
| ***Penicillium viridicatum*** | **CBS 390.48** | **MH867960** |
| *Pestalotiopsis neglecta* | CBS 357.71 | MH871930 |
| *Phanerochaete velutina* | CBS 288.73 | MH878379 |
| ***Phialocephala fortini*** | **CBS 443.86** | **AB671466** |
| *Phlebiopsis gigantea* | CBS 935.70 | MH871798 |
| ***Pichia fermentans*** | **CBS 187** | **KY108804** |
| ***Pichia kluyveri*** | **CBS 188** | **KY108824** |
| ***Pichia kudriavzevii*** | **CBS 5147** | **KY108833** |
| ***Pichia membranifaciens*** | **CBS 107** | **KY108878** |
| ***Pichia terricola*** | **CBS 2617** | **KY108920** |
| *Plectosphaerella cucumerina* | CBS 137.37 | MH867359 |
| ***Purpureocillium lilacinum* (*Paecilomyces lilacinus*)** | **CBS 284.36** | **NG_056277** |
| *Rhizoctonia solani* | CBS 124593 | MH874912 |
| *Rhizophagus intraradices* | AFTOL-ID 845 | DQ273828 |
| *Rhizopus stolonifer* | CBS 382.52 | MH868626 |
| ***Rhodotorula glutinis*** | **CBS 20** | **KY109041** |
| ***Rhodotorula mucilaginosa*** | **CBS 316** | **KY109056** |
| ***Rhodotorula paludigena*** | **CBS 6566** | **KY109142** |
| ***Robbauera albescens*** | **CBS 608.83** | **MH873380** |
| ***Robillarda sessilis*** | **CBS 114312** | **KR873284** |
| ***Saccharomyces bayanus*** | **CBS 380** | **KY109232** |
| ***Saccharomyces cerevisiae*** | **NRRL Y-12632** | **JQ689017** |
| ***Saitozyma flava*** | **CBS 331** | **NG_057649** |
| ***Sarocladium implicatum*** | **CBS 959.72** | **MH878470** |
| ***Sarocladium strictum*** | **CBS 346.70** | **NG_055736** |
| *Schizophyllum commune* | CBS 199.27 | MH877686 |
| *Serendipita indica* | DSM 11827 | NG_059912 |
| ***Simplicillium lamellicola*** | **CBS 116.25** | **MH866307** |
| ***Simplicillium lanosoniveum*** | **CBS 123.42** | **MH867593** |
| ***Simplicillium obclavatum*** | **CBS 311.74** | **NG_042535** |
| *Sordaria fimicola* | CBS 508.50 | AY681160 |
| ***Stachybotrys chartarum*** | **CBS 182.80** | **NG_055746** |
| *Stemphylium solani* | CBS 408.54 | MH868918 |
| *Stilbella aciculosa* | CBS 201.73 | MH872363 |
| ***Talaromyces aculeatus*** | **CBS 289.48** | **MH867897** |
| *Talaromyces flavus* | CBS 387.48 | MH867958 |
| ***Talaromyces funiculosus*** | **CBS 272.86** | **MH873645** |
| *Talaromyces islandicus* | CBS 394.50 | MH868194 |
| *Talaromyces pinophilus* | CBS 170.60 | MH869489 |
| ***Talaromyces purpureogenus*** | **CBS 286.36** | **KY635863** |
| ***Talaromyces ruber*** | **CBS 132704** | **KY635864** |
| ***Tausonia pullulans*** | **CBS 2532** | **NG_042352** |
| ***Tilletiopsis pallescens*** | **CBS 606.83** | **MH873378** |
| ***Tilletiopsis washingtonensis*** | **CBS 544.50** | **MH868275** |
| ***Torulaspora delbrueckii*** | **CBS 1146** | **KY109850** |
| ***Torulaspora globosa*** | **CBS 764** | **KY109865** |
| *Trichoderma asperellum* | CBS 125571 | MH877858 |
| *Trichoderma atroviride* | CBS 185.69 | MH871023 |
| *Trichoderma aureoviride* | CBS 525.63 | MH869965 |
| *Trichoderma deliquescens* | CBS 128260 | MH876310 |
| ***Trichoderma ghanense*** | **CBS 259.85** | **MH873565** |
| ***Trichoderma hamatum*** | **DAOM 167057** | **HM466686** |
| ***Trichoderma harzianum*** | **CBS 226.95** | **HM466680** |
| *Trichoderma koningii* | CBS 850.68 | MH878411 |
| *Trichoderma lixii* | CBS 126411 | MH875739 |
| ***Trichoderma longibrachiatum*** | **CBS 816.68** | **NG_056276** |
| *Trichoderma piluliferum* | CBS 814.68 | AF400739 |
| ***Trichoderma pseudokoningii*** | **CBS 408.91** | **AF400740** |
| ***Trichoderma reesei*** | **CBS 383.78** | **MH872915** |
| ***Trichoderma spirale*** | **CBS 346.93** | **MH874070** |
| ***Trichoderma strictipile*** | **CBS 347.93** | **NG_057688** |
| *Trichoderma stromaticum* | CBS 126600 | MH877899 |
| ***Trichoderma virens*** | **CBS 249.59** | **MH869393** |
| *Trichoderma viride* | CBS 127113 | MH875860 |
| *Trichoderma viridescens* | CBS 433.34 | MH867105 |
| *Trichothecium roseum* | CBS 567.50 | MH868279 |
| *Typhula phacorrhiza* | EL43_99 | AY586724 |
| *Verticillium albo-atrum* | CBS 452.51 | MH868462 |
| *Verticillium biguttatum* | CBS 848.70 | MH871772 |
| *Verticillium dahliae* | CBS 204.26 | MH866383 |
| *Verticillium tricorpus* | CBS 238.75 | MH872647 |
| ***Wickerhamomyces anomalus*** | **CBS 5759** | **KY110078** |
| *Xylaria hypoxylon* | CBS 120.16 | MH866173 |
| ***Zygosaccharomyces bailii*** | **CBS 680** | **KY110234** |
